# In vivo oxygen measurement in cerebrospinal fluid of pigs to determine physiologic and pathophysiologic oxygen values during CNS infections

**DOI:** 10.1186/s12868-021-00648-x

**Published:** 2021-06-28

**Authors:** Nicole de Buhr, Alexander Martens, Marita Meurer, Marta C. Bonilla, Franz Söbbeler, Lara Twele, Stephan Neudeck, Michael Wendt, Andreas Beineke, Sabine Kästner, Maren von Köckritz-Blickwede

**Affiliations:** 1 Department of Biochemistry, University of Veterinary Medicine Hannover, Hannover, Germany; 2 Research Center for Emerging Infections and Zoonoses (RIZ), University of Veterinary Medicine Hannover, Hannover, Germany; 3 Small Animal Clinic, University of Veterinary Medicine Hannover, Hannover, Germany; 4 Clinic for Horses, University of Veterinary Medicine Hannover, Hannover, Germany; 5 Clinic for Swine and Small Ruminants, Forensic Medicine and Ambulatory Service, University of Veterinary Medicine Hannover, Hannover, Germany; 6 Department of Pathology, University of Veterinary Medicine Hannover, Hannover, Germany

**Keywords:** Oxygen, Meningitis, Cerebrospinal fluid, Hypoxia, Physioxia, Normoxia

## Abstract

During infection and inflammation, a reduced oxygen level clearly affects cellular functions. Oxygen levels during CNS infections are unknown. Here we established and evaluated an in vivo measurement system to characterize the oxygen level in parallel with bacterial numbers (CFU/mL), the cell number and pH level inside the CSF of healthy compared to *Streptococcus suis*-infected pigs. The animals were anesthetized over a seven-hour period with isoflurane in air/oxygen at physiologic arterial partial pressure of oxygen. Oxygen levels in CSF of anesthetized pigs were compared to euthanized pigs. The detected partial pressure of oxygen in the CSF remained constant in a range of 47–63 mmHg, independent of the infection status (bacterial or cell number). In contrast, the pH value showed a slight drop during infection, which correlated with cell and bacterial number in CSF. We present physiologic oxygen and pH values in CSF during the onset of bacterial meningitis.

**Supplementary Information:**

The online version contains supplementary material available at 10.1186/s12868-021-00648-x.

## Introduction

Oxygen is a key element in all metabolic processes of living beings and is needed in all cells. The oxygen supply has species-independent significant effects on many cellular processes. A decrease in oxygen supply in case of infection and inflammation can influence the host–pathogen interaction as well as pathogenesis of various diseases [1–3]. The influence of oxygen on the function of immune cells such as neutrophils and mast cells was already well characterized [3–8]. To investigate functions of immune cells and pathogen behavior under proper hypoxic conditions, it is necessary to characterize the exact oxygen level at physiologic and pathophysiologic conditions in vivo. Data of physiologic oxygen values (oxygen partial pressure = pO_2_) already exist for numerous human tissues and demonstrate a high variability in oxygen levels in different tissues [9, 10]. Whereas in the kidney, pO_2_ values of 72 ± 20 mmHg [9, 11] were found, inside the lung, the value is 42.8 mmHg [12] and inside the brain values of up to 33.8 ± 2.6 mmHg occur [9, 13]. Knowledge concerning pathophysiologic in vivo oxygen conditions is limited. Inside tumors, the oxygen level ranges between 2–32 mmHg [14]. Measurements taken inside the lung from cystic fibrosis patients infected with *Pseudomonas aeruginosa* had a mean pO_2_ value of 2.5 mmHg [15]. However, it is unclear whether these low values are related to the infection. Thus, oxygen values in infected host compartments with infiltrating immune cells are still unclear. Regarding central nervous system (CNS) infections, a better molecular understanding is needed to study the complex interaction of defense cells and pathogens after entry into the cerebrospinal fluid (CSF) compartment to search for new therapeutic strategies. Especially during bacterial meningitis, a high amount of neutrophils infiltrate into the CSF to counteract against bacteria [16]. Whereas data on oxygen in brain tissue of healthy rats are available [17], no data on dissolved physiological relevant oxygen in CSF during infection can be found in the literature.

Nevertheless, these values are needed to optimize in vitro systems, such as blood–brain-barrier models [18–24], to better reflect the in vivo situation.

Oxygen highly influences host cells and the host–pathogen interaction [25]. The respective interaction of pO_2_ and the needs or adaptability of the tissue type and thus the host–pathogen interaction must therefore be considered in a much more differentiated way than before. Thus, in future, molecular studies on the cellular host–pathogen interaction, considering physiologic and pathophysiologic oxygen conditions, can be used to characterize adequately new therapeutic target structures against infectious diseases in humans and animals. Since there is insufficient knowledge about physiologic and pathophysiologic oxygen levels in humans and animals, in vivo characterization is necessary to optimize conditions for in vitro studies and therefore finally to reduce the number of animal experiments.

The aim of the present study was to establish an in vivo oxygen measurement in CSF of pigs for detection of physiologic relevant oxygen values in CSF in healthy animals and during the onset of a bacterial infection of the CNS. The Gram-positive bacterium *Streptococcus* (*S.*) *suis* was used as a zoonotic model pathogen that can cause meningitis besides a wide range of symptoms in humans and pigs[26–28]. A luminescence-based method measuring oxygen levels in CSF using oxygen-sensitive sensors in infected and non-infected animals was applied. For this purpose, we adapted a methodology, which had been well characterized during several in vitro studies [1, 4–6, 29] to enable a combined in vivo measurement system. This system allowed to determine pO_2_, pH, cell number/mL and colony-forming units (CFU)/mL CSF in real-time without surgical intervention.

## Results

### Key facts for establishing the measurement system in CSF

**Fig. 1:**
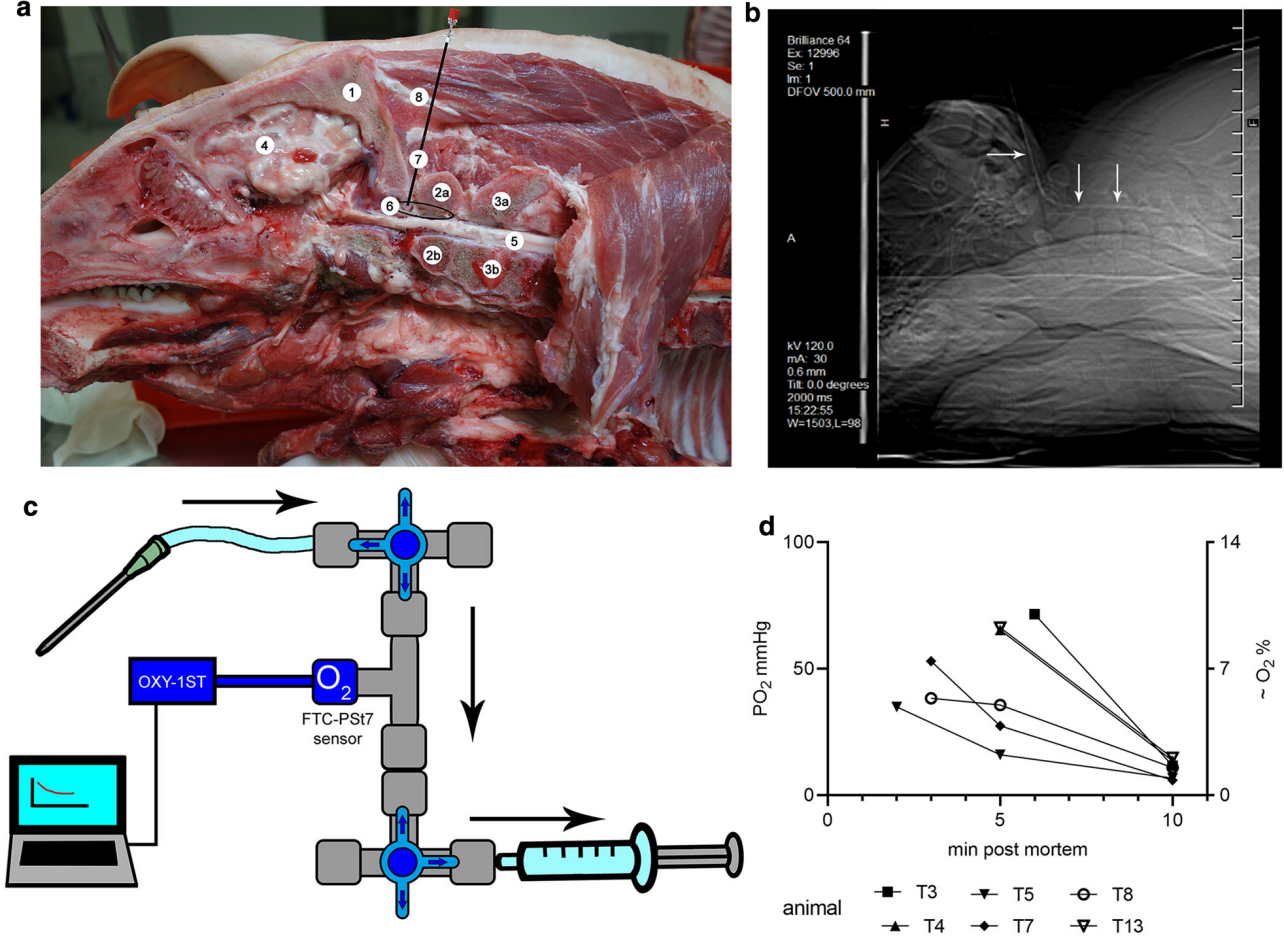
Oxygen values in the CSF of pigs decrease rapidly over time after death.** a** The position of the needle inside the CSF compartment in the region of the *Articulatio atlantooccipitalis* is demonstrated with a halved carcass of a pig. 1 = *Os occipitale*, 2a = *Atlas* (*Arcus dorsalis*), 2b = *Atlas* (*Arcus ventralis*), 3a = *Axis* (*Processus spinosus*), 3b = *Axis* (*Corpus*), 4 = *cerebrum* (*brain*), 5 = *Medulla spinalis* (spinal cord), 6 = *Cisterna cerebellomedullaris*, 7 = *Spatium atlantooccipitale*, 8 = approximate position of the needle for CSF aspiration. **b** Computer tomography image of a catheter inserted through the *Spatium atlantooccipitale* into the *Cisterna cerebellomedullaris* and the subarachnoid space. White arrows mark the catheter. **c** Oxygen measurement system: Connected with a flexible tube to the needle located inside the CSF. Black arrows show CSF flow into the syringe. The dissolved oxygen is measured with the oxygen (O_2_) Flow-Through-Cell (FTC)-PSt7-sensor connected to the OXY-1 ST device. **d** Each symbol represents a freshly drawn CSF sample of one pig. Values of one pig are connected by a line, not reflecting constant measurement. The values depict a continuous decrease in oxygen over time after death with high variability

To harvest CSF from pigs without surgical intervention, two sites are accessible. The first position is the spinal tap at the same position as the lumbar puncture in humans, which reflects a safe puncture point of the *Cisterna lumbalis* at the lumbosacral space. The second access to CSF in pigs is possible by puncturing the *Cisterna cerebellomedullaris* at the *atlanto-occipital* space, which is a possible access point in humans as well. As the *Cisterna cerebellomedullaris* is closer to the brain, this study focused on establishing the oxygen measurement system at this localization. As artificially infiltrated blood components in CSF can lead to false results, we made an effort to gain pure CSF with diminished blood contamination. Therefore, the access procedure was standardized and trained with pig carcasses. An optimal needle position with access to the *Cisterna cerebellomedullaris* was attainable through the *Spatium atlantooccipitale*. The position of the needle is described for the sagittal cut carcass of a pig (Fig. 1a). The correct position was verified by computer tomography (CT) of an epidural catheter inserted into the subarachnoid space (Fig. 1b and Additional file 1: Video S1). As the epidural catheter does not allow withdrawal of CSF, we used a Tuohy epidural needle (Spinocan, 18 G 3 ½).

Direct air contact of the CSF could increase oxygen in CSF, as oxygen is absorbed into liquid by surface-liquid agitation and direct diffusion. To avoid false results for oxygen diffusion from air contact, a closed measurement system with three-way valves was connected directly to the needle and allowed CSF to be withdrawn using an FTC-PSt7 oxygen sensor to determine dissolved oxygen inside the CSF. The Tuohy needle was inserted into the CSF compartment. Clear CSF occurring at the needle hup was used as an indicator for of the correct needle positioning, and the needle was immediately connected to the described closed measurement system (Fig. 1c).

Since the temperature of the liquid highly influence oxygen determination (see Additional file 2: Figure S1), the rectal body temperature was measured and used for automatic correction of the measurement results by the oxygen measurement device.

### After circulatory failure (death) of pigs, oxygen values in the CSF rapidly decrease over time

Based on the recommendations made by the manufacturer of the oxygen measurement device (PreSens Precision Sensing GmbH Regensburg, Germany), we decided to include a one-minute equilibration period for oxygen measurements after the CSF had come into contact with the sensor. As a first step, we aimed to see how fast the oxygen partial pressure dropped after having euthanized the animals to see if for ethical reasons the measurement could be done immediately after euthanizing the animals. High variability between the animals was seen during this measurement. However, a clear time-dependent drop in the oxygen level was visible in all animals immediately after death (Fig. 1d; all raw data can be found in Additional file 3: Table S1). After ten minutes *post mortem*, in all pigs, the oxygen partial pressure dropped to a mean of 10.45 mmHg (≜1.5% O_2_). Thus, we concluded that oxygen measurements in euthanized animals was not possible due to high variability and immediate oxygen drop based on cardiac arrest.

### Optimization of the system for a real-time determination of physiologic and pathophysiologic parameters in the CSF of infected and uninfected pigs during long-term anesthesia in vivo

**Fig. 2:**
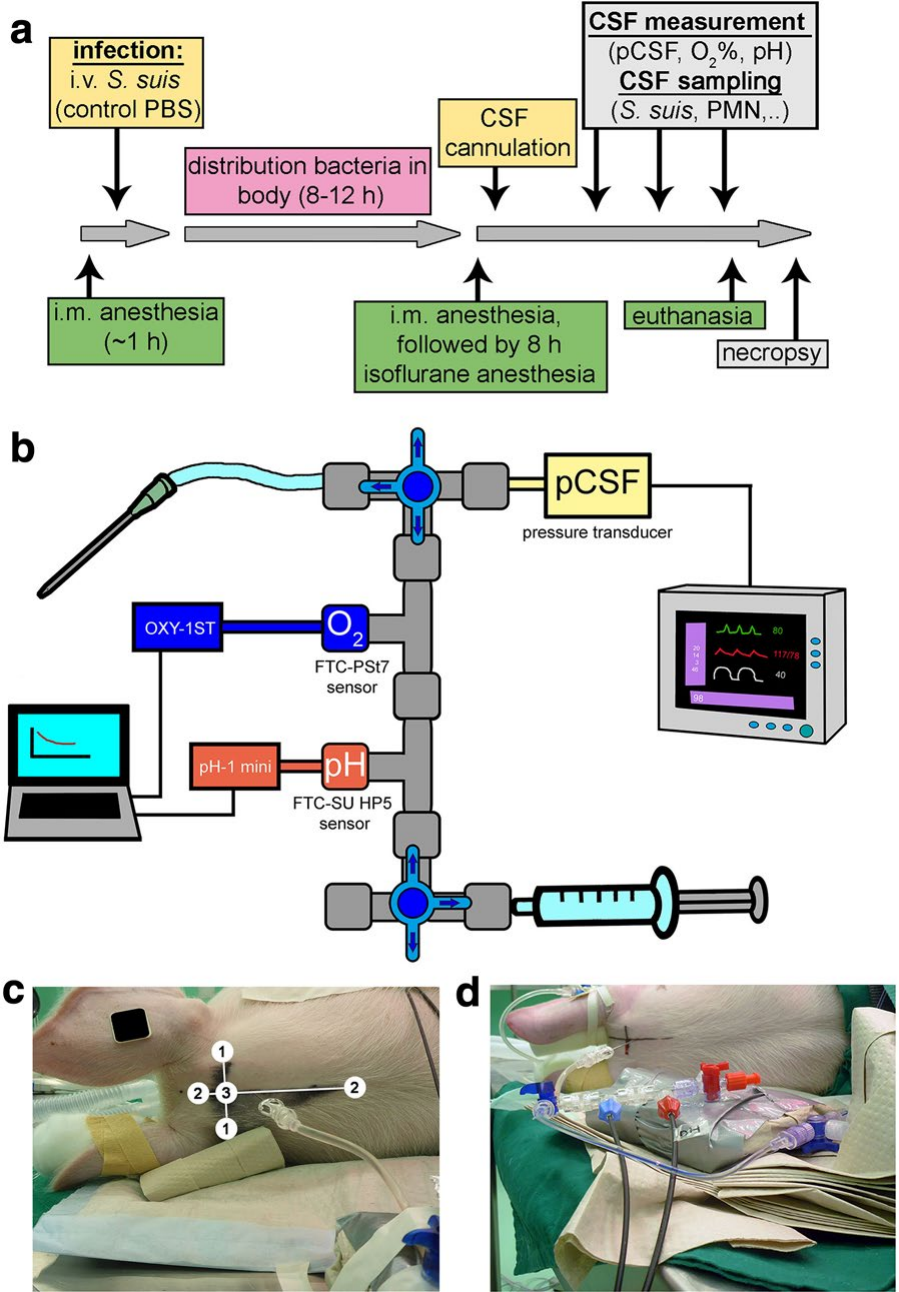
Overview of experimental setup and measurement system to determine physiologic and pathophysiologic parameters in CSF. **a** Experimental set-up of infection to cause meningitis in pigs. Pigs were infected intravenously with *S. suis* or mock infected with PBS. After a fixed time-frame of eight-12 h post infection, all pigs were anesthetized with isoflurane for eight h under controlled conditions. A needle was inserted into the CSF compartment and connected to the measurement system shown in b. One, four and seven hours after begin of the anesthesia the measurement and sampling were conducted. Finally, pigs were euthanized, and a necropsy was performed. **b** The measurement system was connected to the needle. Oxygen and pH sensors were connected to specific read-out systems (OXY-1 ST device and pH-1 mini) and a laptop to collect data. A pressure transducer was connected to the anesthesia multiparameter monitor. Three-way valves allowed directing the flow of the CSF to the sensors, into sampling tubes and to the pressure transducer. Furthermore, a cleaning procedure of the system between the measurement points was possible without contaminating the CSF. Detailed information can be found in Additional file. **c** During the time of isoflurane anesthesia, the pig was fixed in a lateral position with maximal bent head to the sternum. The intersection point (3) was identified by two lines between points 1 and 2. The position of the needle (1 cm caudal of the intersection point) was never changed during the experiment. **d** The measurement system connected to the needle inside the subarachnoid space of a pig under anesthesia is shown

Since the values of dissolved oxygen varied considerably and decreased rapidly after death, the method was further developed to determine physiologic and pathophysiologic parameters in CSF of pigs under controlled isoflurane air/oxygen anesthesia. After intravenous *S. suis* infection, the pigs were anesthetized at a predetermined time-point or as soon as clinical signs of CNS symptoms or lameness were visible. The pigs were anesthetized at the latest 12 h post infection or due to animal welfare reasons between eight-12 h post infection (Fig. 2a). An extended measurement system including measurement of CSF pressure (pCSF in mmHg) as well as pH was used (Fig. 2b and Additional file 2: Figure S2), allowing the calculation of oxygen in relation to the ambient barometric and additional CSF pressure (Additional file 2: Figure S3). Furthermore, the extension with the FTC-SU HP5 sensor enabled the pH measurement.

Upon successful needle placement in the *Cisterna cerebellomedullaris* as described above, the measurement system as shown in Fig. 2b was immediately connected. The spinal needle was left in place in the CSF compartment during the entire experiment as shown in Fig. 2c. The measurement set-up was protected against light with aluminium foil as presented in Fig. 2d.

As the measurement system remained unaltered during the ongoing experiment in one piglet, a cleaning process of the whole system was always conducted to avoid bacterial growth inside the system. This cleaning set up (Additional file 2: Figure S4) was tested in vitro (Additional file 2: Figure S5) and showed an efficient cleaning procedure with no bacterial growth over time inside the measurement system. After cleaning, the sensor was equilibrated with CSF five minutes before the subsequent measurement time-point. To avoid a temperature drop inside the measurement system, the CSF was replaced again with freshly drawn CSF, and the following one-minute cut-off value was used as a final read-out for the statistical analysis (see above). Different options for cut-off values are presented in Additional file 4: Table S2 and Additional file 5: Table S3. Nonetheless, these did not show significant differences (Additional file 6: Table S4). Overall, this measurement was defined as two-step equilibration.

**Fig. 3:**
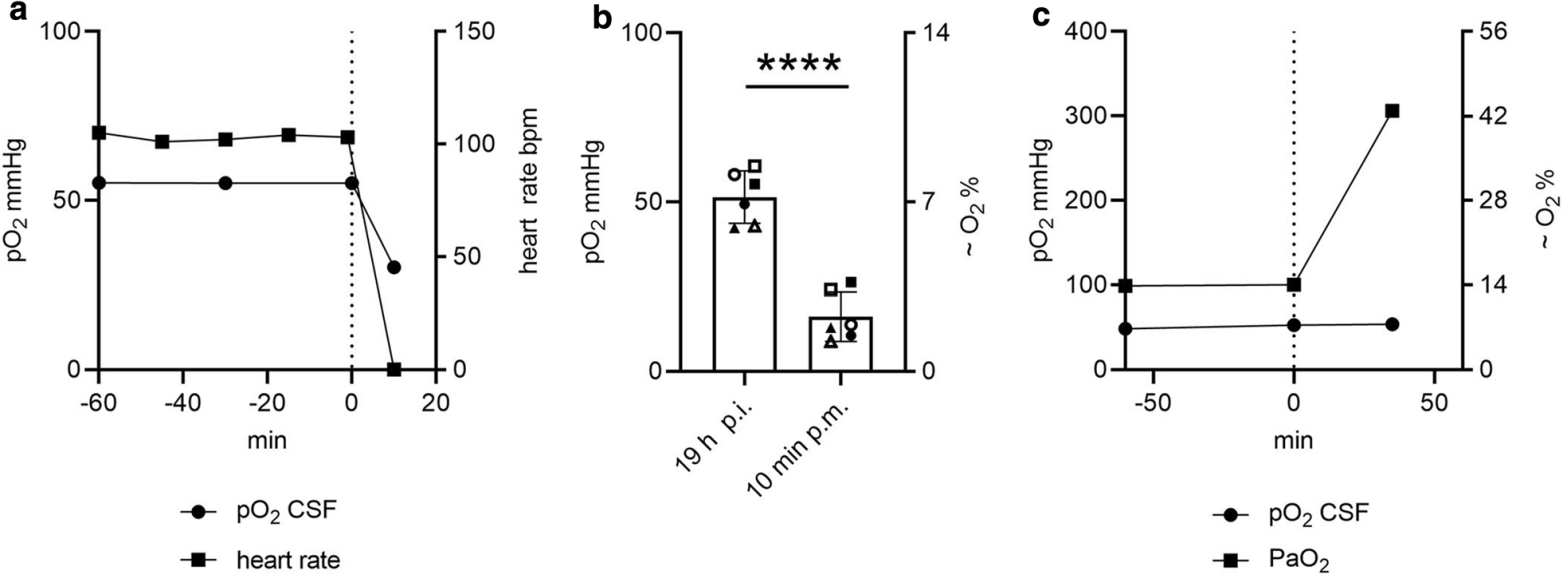
Dissolved oxygen in CSF is immediately influenced by the death of the pig. All data presented in this graph were collected in pigs under isoflurane anesthesia. The needle for measuring oxygen inside the CSF was permanently fixed as described in Fig. 2. All data present the oxygen value after a two-step equilibration. The oxygen mmHg was calculated based on the rectal temperature and the current air pressure and pressure inside the CSF compartment. **a** The heart rate of the pig was continuously measured. The oxygen inside the CSF compartment was measured at each dot with freshly drawn CSF. After the pig had been euthanized (0 min), the oxygen inside the CSF rapidly decreased (n = 1). **b** The oxygen inside the CSF significantly decreased 10 min post mortem compared to a value determined some minutes before the pig was euthanized (19 h post infection (p.i.)). Each symbol represents one pig. Filled symbols represent infected pigs, unfilled symbols represent uninfected pigs. Error bars are presented with ± SD (n = 6, paired, one-tailed Student’s t-Test, *P* < 0.0001). **c** A hyper-oxygenation of the pig over a 30-min period did not increase the oxygen level inside the CSF, whereas inside the blood, an increase was detectable (n = 1)

To finalize optimization of real-time in vivo measurements as a control experiment, the influence of hypoxemia or the cessation of blood flow by cardiac arrest was tested. Similar to the phenomenon of oxygen drop in euthanized animals (Fig. 1d), cardiac arrest and therefore transport of oxygenated blood immediately influenced the pO_2_ in CSF (Fig. 3a). The CSF pO_2_ was measured *pre* and *post mortem* with the two-step equilibration to overcome measurement variances (Fig. 3b). The value decreased from *pre mortem* 51.47 mmHg (≜ 7.35 O_2_%) to 16.11 mmHg (≜ 2.31 O_2_%) 10 min *post mortem.* However, artificial hyperoxygenation and hypoxemia over a 30 min period did not change the pO_2_ in CSF in living pig (Fig. 3c). Although blood oxygenation highly increased, oxygen values in CSF were unchanged.

### Data output: Real-time determination of physiologic and pathophysiologic parameters in the CSF of infected and uninfected pigs during long-time anesthesia in vivo

**Fig. 4:**
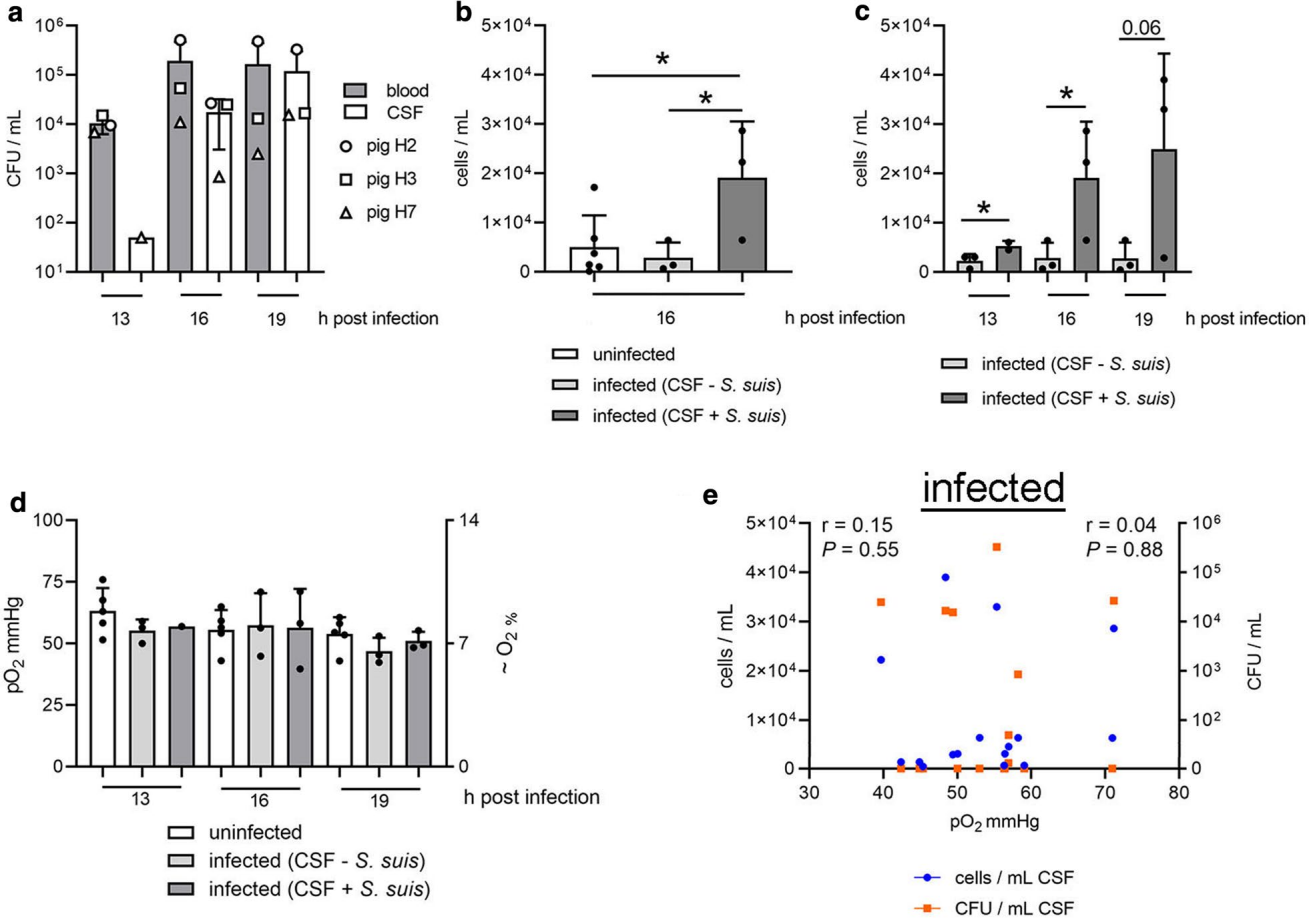
Oxygen in CSF is not decreased during early phase of *S. suis* meningitis in pigs. **a** In three of six infected pigs, bacteria were detectable inside the CSF and blood, reflecting the onset of meningitis. The CFU/mL increased over time inside the CSF. b-c) By flow cytometry, cells/mL inside the CSF were measured and granulocyte-specific cells are presented. **b** Sixteen h post infection, significantly more granulocytes were detected inside the CSF of infected pigs with bacteria inside the CSF compared to uninfected pigs (unpaired, one-tailed Student’s *t*-Test, n_uninfected_ = 6, n_infected_ = 3 in each group). **c** During all analyzed time-points, significant or remarkably higher amounts of granulocytes were detected inside the CSF of infected pigs, with bacteria inside the CSF compared to infected pigs without bacteria inside the CSF (unpaired, one-tailed Student’s *t*-Test, n = 3 in each group). **d** The amount of oxygen measured inside the CSF-compartment showed no significant differences between the three groups at all time-points (ANOVA table was calculated for each time point). **e** During the onset of meningitis, no correlation between oxygen and granulocytes (*r* and *P* values left side) or bacteria (*r* and *P* values right side) was identified in CSF of infected animals. Calculation is based on all parameters from all animals and time-points with a two-tailed nonparametric Spearman r correlation calculation (n = 16). In all graphs, error bars are presented with ± SD. In **b**–**d**, each dot reflects one animal (*P < 0.05)

To confirm successful infection, we determined the colony-forming units (CFU/mL) of *S. suis* in the blood and CSF as well as the number of granulocytes (cells/mL) in CSF. In 50% of the infected pigs, *S. suis* were detectable in the blood and CSF (Fig. 4a) and a significantly higher number of granulocytes was detected in CSF of pigs infected with *S. suis* in CSF (Fig. 4b, c).

After confirming successful infection, including quantification of CFU/mL and cells/mL in CSF, we determined the physiologic and pathophysiologic pO_2_ values inside the CSF. All data presented in the figures are based on two-step equilibration of oxygen and pH sensors. All raw data are presented in Additional file 4: Table S2 and Additional file 5: Table S3. The comparison of the pO_2_ detected inside the CSF of all infection groups showed no significant difference (Fig. 4d). The mean oxygen values at the time-point 13 h p.i. in uninfected pigs, in infected pigs without bacteria in the CSF and in infected pigs with bacteria in the CSF were 63.31 mmHg (≜ 9.04 O_2_%), 55.17 mmHg (≜ 7.87 O_2_%) and 56.94 mmHg (≜ 8.13 O_2_%), respectively. No significant change was detectable over time or between the groups. When comparing the data with CFU/mL or cells/mL in CSF, neither a correlation between pO_2_ values and the number of granulocytes inside CSF nor between pO_2_ values and bacteria inside CSF was detectable (Fig. 4e). However, there was an expected significant positive correlation between bacteria and granulocytes inside the CSF (Spearman r = 0.75; *P* = 0.0006) confirming the typical host reaction during the early phase of *S. suis* meningitis [18, 30]. In summary, our data showed that dissolved oxygen in CSF was not decreased during the onset of *S. suis* meningitis in pigs under the chosen conditions.

In addition, no significant differences in CSF pH were detectable over time or between infection groups (Additional file 2: Figure S6). However, the mean pH level (all time-points, all animals) in the uninfected group (pH = 7.3) and in the infected group with no bacteria in the CSF (pH = 7.4) was higher compared to the infected group with bacteria in the CSF (pH = 6.96). When considering the correlation between the pH level and bacteria or cells inside the CSF, a significant negative correlation was detectable. This means that the more bacteria or cells were present in the CSF, the further the pH decreased.

### Influence of ventilation and bacteria on anesthetic management and histological detectable changes

The status of all animals was during the anesthesia frequently checked for abnormalities. Only minor adjustments in ventilatory settings and fraction of inspired O_2_ (F_i_O_2_) were necessary to maintain an arterial partial pressure of oxygen (paO_2_) between 80 to 110 mmHg (Additional file 7: Table S5 shows for each time point the individual pO_2_ mmHg in CSF and blood). None of the pigs developed signs of acute respiratory distress syndrome during the trial. Overall, 5/12 pigs (3 uninfected, 2 infected (1 CSF + *S. suis*, 1 CSF- *S. suis*)) required dopamine hydrochloride at a mean (SD) dose of 2 (2.7), 6.0 (2.2) and 5 (0.0) µg/kg/min at 13 h, 16 h, and 19 h after infection, respectively. Values for arterial partial pressure of CO_2_ (paCO_2_), end tidal CO_2_ (Pe´CO_2_), paO_2_, F_i_O_2_, paO_2_/F_i_O_2_, tidal volume and respiratory rate (*f*r) for uninfected, infected (CSF—*S. suis*) and infected (CSF + *S. suis*) are displayed in Table 1. Only one pig [infected (CSF + *S. suis*)] showed signs of sepsis with hyperthermia [up to 41 °C (reference 38.5–39.5 °C)] starting 5.5 h after induction, tachycardia up to 180 beats per minute prior to euthanasia, hypotension (mean arterial blood pressure of 54 mmHg), requiring vasopressor support with dopamine hydrochloride (5 µg/kg/min) from the beginning of anaesthesia and additional noradrenaline (0.2 µg/kg/min) for the last 4 h of anaesthesia. The paO_2_/F_i_O_2_ values decreased slightly for this pig, with values one hour after induction, 4 h after induction and 7 h after induction of 402, 309 and 317 mmHg, respectively.

**Table 1:** Mean ± standard deviation of respiratory rate (*f*r), fraction of inspired oxygen (F_i_O_2_), arterial partial pressure of oxygen paO_2_/F_i_O_2_ quotient (Horoviz quotient), tidal volume (VT), arterial partial pressure of CO_2_ (paCO_2_) and end tidal carbon dioxide (Pe´CO_2_) of uninfected, infected (CSF−*S. suis*) and infected (CSF + *S. suis*) pigs (n_uninfected_ = 6, n_infected_ = 3 in each group) 13, 16 and 19 h post infection

	fr (breaths minute^−1^)			F_i_O_2_			paO_2_/F_i_O_2_ (mmHg)		
Post infection	13 h	16 h	19 h	13 h	16 h	19 h	13 h	16 h	19 h
Uninfected	18.3 ± 2.3	18 ± 2.7	17.6 ± 3.6	0.34 ± 0.14	0.36 ± 0.06	0.33 ± 0.1	330 ± 40	275 ± 71	293 ± 89
Infected (CSF—*S. Suis*)	17.3 ± 3.2	18 ± 3	18 ± 3	0.25 ± 0.03	0.28 ± 0.04	0.28 ± 0.04	401 ± 64	359 ± 44	346 ± 44
Infected (CSF + *S. Suis*)	19 ± 1	19.7 ± 1.5	20.3 ± 1.2	0.28 ± 0.05	0.28 ± 0.01	0.29 ± 0.01	378 ± 52	342 ± 29	379 ± 75

	VT (ml kg^−1^)			paCO_2_ (mmHg)			Pe´CO_2_ (mmHg)		
Post infection	13 h	16 h	19 h	13 h	16 h	19 h	13 h	16 h	19 h
Uninfected	13.2 ± 2	14.3 ± 2.1	13.8 ± 2	42.8 ± 7.3	41 ± 3.6	41.6 ± 2.4	41.5 ± 6.3	40.5 ± 3.4	39.8 ± 3.1
Infected (CSF—*S. Suis*)	11.7 ± 2	12.2 ± 2.4	12.2 ± 2.4	38.1 ± 2.2	42.2 ± 4.7	45.2 ± 4.9	39.3 ± 0.6	40.7 ± 1.5	41.7 ± 3.1
Infected (CSF + *S. Suis*)	12.3 ± 2	12.3 ± 2.3	12.6 ± 0.4	47.7 ± 1.5	49.3 ± 3	45.3 ± 5.8	48.3 ± 0.6	48.7 ± 0.6	43.7 ± 3.5

In four of six infected pigs the infection strain of *S. suis* was reisolated from an inner organ or from serosa or from joint fluid, indicating an undergoing infection (Additional file 8: Table S6). However, by histological examination of different organs and serosa no hint for damage by insufficient oxygenation was detectable including the above described animal with clinical signs of sepsis. In some animals typical *S. suis* induced neutrophil infiltrations were observed (Table 2).

**Table 2:** Scoring of histological findings of pigs uninfected and infected with *S. suis* serotype 2

Age of infected pigs (wk)	Total no. of pigs	Sex (f/m)	*S. suis* strain	No. of pigs/Total no. of pigs												ɷ^e^
				Brain			Serosa			Spleen and liver			Lung			
				Meningitis, choroiditis			Pleuritis or peritonitis			Splenitis^a^ or hepatitis			Pneumonia			
				5^b^	3^c^	1^d^	4^b^	2^c^	1^d^	4^b^	2^c^	1^d^	4^b^	2^c^	1^d^	
7–8	6	(3/3)	Uninfected	0/6	0/6	0/6	1/6	0/6	0/6	0/6	0/6	0/6	1/6	0/6	0/6	0.66
7–8	6	(1/5)	10	1/6	1/6*	0/6	0/6	0/6	0/6	0/6	2/6	0/6	1/6	0/6	0/6	1.17

*Minimally focal/multifocal lymphoplasmacellular *Plexus chorioiditis* (not in score)

^a^Neutrophilic accumulation in the splenic red pulp

^b^Scoring of 4 and 5 indicates moderate to severe diffuse or multifocal fibrinosuppurative inflammations

^c^Scoring of 2 and 3 indicates mild focal fibrinosuppurative inflammation

^d^Individual single perivascular neutrophils received a score of 1

^e^ω = ∑score_max_ / *n*_animals_[50]

wk = weeks; f = female; m = male

In summary, we established and evaluated a real-time measurement of pO_2_, pH level and different parameters such as CFU/mL and cells/mL to determine physiologic values inside the CSF of living infected animals under anesthesia.

## Discussion

To reduce the number of animal experiments, in vitro systems need to be optimized by adjusting these to physiologically relevant oxygen levels. One critical parameter is the dissolved oxygen, which varies in different regions of the body and furthermore can be influenced by infections [9]. Two oxygen states can be distinguished in vivo: 1. Physoxia, describing a situation with physiologic oxygen values for specific tissue or body fluid and 2. Hypoxia, describing a situation with pathophysiologic oxygen values in tissue or body fluid [31]. Examples of these conditions are a local infection with presence of oxygen consumers, a reduced blood circulation (e.g., stroke) or a changed blood circulation (e.g., tumor growth). Hyperoxic conditions occur when oxygen is higher compared to the physiologically relevant oxygen conditions. This is what happens in a normal tissue culture incubator, since it is maintained at 5% CO_2_, resulting in oxygen values of ≜18.6% O_2_ [31]. Thus, these hyperoxic oxygen levels in the normal tissue incubator are far away from being physiologically relevant. It was shown in several studies that oxygen values differing from physiologically relevant values not only significantly influence the metabolism of cells, but can also alter growth rate and behavior of cells [6, 32–34] as well as influence the response of the host to infections as well as the infection rate of bacteria [6, 35].

The infection site during meningitis is the CSF, which is produced by the choroid plexus cells, circulating around the brain, inside the ventricles and around the spinal cord. This compartment is highly protected by the brain barriers and the entrance or diffusion of cells, ions and substrates are strictly regulated by these barriers [36]. The diffusion of substrates like drugs can already be mimicked by cell culture systems of the brain barriers [37, 38]. However, knowledge about the transport of dissolved oxygen molecules into the CSF compartment, especially during infections of this compartment is limited. A direct connection and transport of oxygen from the arterial blood into the CSF was experimentally proven already in 1968 by Kazemi and colleagues [39]. In their study, they measured oxygen with electrodes ex vivo in CSF drawn from the cisterna and ventricles in anesthetized dogs, comparing the oxygen values with arterial and venous blood oxygen values. This study was able to verify data from a previous published study in sheep [40]. Therefore, oxygen transport mainly takes place in the ventricles, where the blood cerebrospinal fluid barrier is located [40]. Furthermore, decreased pO_2_ in arterial blood due to chronic lung disease with hypercapnia in humans did not influence the lumbar measured pO_2_ in CSF (with chronic lung disease: 39.0 ± 6.9 mmHg, without lung disease 39.5 ± 5.8 mmHg) [41]. Interestingly, studies about the pre-Bötzinger complex showed that a central chemo-sensor for hypoxia exists inside the CNS [42]. The pre-Bötzinger complex is part of the respiratory center and located in the region of the ventral medulla of the brainstem [43]. The complex is described to generate the respiratory rhythm in mammals [42–44]. Therefore, it can be hypothesized that decreasing oxygen values inside the CSF can be compensated to a certain extent.

Nevertheless, whether or not oxygen is permanently regulated inside this compartment during infections with pathophysiological increased oxygen consumption (e.g., bacteria and infiltrating immune cells) inside the CSF is unknown.

To determine values in CSF of pO_2_, pH, cells/mL and CFU/mL as well as to collect CSF samples for later analysis of deep-frozen CSF samples, we established the described system (Fig. 2). Taking such a measurement in euthanized pigs proves inaccurate as it leads to highly varying results due to the failure of oxygen transport by the bloodstream and the irregular behavior of dying cells (Fig. 1). This system describes a method to determine pathophysiologic pO_2_ and pH values during the onset of meningitis and enables a correlation of all parameters (Fig. 4e and Additional file 2: Fig. S6b). It was previously described that an oxygen gradient exists inside the CSF compartment. In healthy dogs, the highest pO_2_ value was found inside the ventricles (69 mmHg) and a slightly lower value was detected inside the cisterna (58 mmHg), decreasing further inside the sagittal sinus [39]. The pO_2_ values in CSF from lumbar puncture in humans (breathing in room air) vary highly depending on the used methods from 39.0 ± 6.9 mmHg to 77 ± 8.7 mmHg [41, 45]. We decided to use the *cisterna cerebellomedullaris* at the atlantooccipital joint as the puncture localization for the CSF compartment the. This was a compromise for an easily accessible point compared to ventricles and a close localization to the host–pathogen interaction region during meningitis (meninges and blood brain barriers). One limitation of this puncture localization in the pig is that several blood vessels can be injured during the process of gaining access to the CSF compartment. This is a critical point in the whole method, which can be avoided by training. If a blood vessel is affected during puncturing of the CSF compartment, blood cells could contaminate the CSF compartment. In case blood cell contamination occurs at the first measurement point, and the bleeding has stopped, the turnover rate of CSF [46] may result in a “cleaning” of the CSF. Therefore, the permanent measurement system and the established cleaning set-up during the seven-hour measurement (Additional file 2: Figure S4 and S5) may contribute to successful measurements at later time-points.

Finally, the negative correlation of the pH level with cell number and CFU is of interest (Additional file 2: Figure S6). Generally, the pH level of CSF may be influenced by external ventilation [47]. Here, all animals underwent the same ventilation protocol. Thus, changes in pH levels of infected versus uninfected animals cannot be explained by the ventilation process. As we found a positive correlation between the cell number and CFU/mL inside the CSF, the CFU/mL could explain the decrease in the pH level as metabolism products of *S. suis* can induce CSF acidification.

## Final conclusion

Taken together, the presented method allows the combined measurement of four parameters to determine physiologically relevant parameters in the onset of meningitis. Our values show in the early phase of *S. suis* meningitis that the oxygen levels inside the CSF compartment of pigs is not significantly different to healthy pigs under the same conditions. In the present study, we detected range of 47–63 mmHg in all animals. Therefore, it would be useful to employ this oxygen value in cell culture systems with a CSF compartment. We detected infiltrating bacteria and immune cells like neutrophils. However, this oxygen consumption did not decrease the pO_2_ to hypoxic conditions. This seems to be unique for CSF, as this was not the case in the intestine [25]. One explanation could be that the pre-Bötzinger complex inside the brain helps to control and stabilize pO_2_ at least during the onset of meningitis. For ethical reasons to reduce burden of animals, a later phase of meningitis was not studied. Thus, a clear differentiation between physiological values during the onset of disease compared to pathophysiological values during a later severe stage of infections cannot be made.

The described method is usable for similar studies to characterize oxygen level during CNS diseases in animals. In summary, these data will help to adjust in vitro systems to physiological and/or pathophysiological relevant parameters. This would allow to investigate the host–pathogen interaction in an almost in vivo situation.

## Methods

### Animal data

All pigs and pigs used in this study came from a conventional herd kept on a farm as part of our university (Farm for Education and Research Ruthe, University of Veterinary Medicine Hannover) and included females and males. The pigs were bought and the management of the farm agreed to use the animals in animal experiments in this study.

Part A: Initially, measurement trials in cerebrospinal fluid (CSF) taps of healthy euthanized pigs were conducted *post mortem*. The pigs were three to five months old and had a body weight of approximately 70 ± 20 kg.

Part B: For the in vivo measurement in CSF as well as part of the *post mortem* measurement in CSF the pigs were housed in groups. The pigs had an average age of eight weeks and a body weight of 17 ± 2 kg at the day of infection in the case of the in vivo measurement. The health status of the pigs was checked, and the animals fed twice per day. Water was available ad libitum.

It was important to use in this study animals from a specific age, as *S. suis* infection rate depend on the age of the animals. The lowest antibody level against *S. suis* can be observed between weeks 6 and 8, presumably corresponding to a decrease in maternal immunity. A marked increase can be seen at 10 weeks of age, shortly after the onset of clinical signs in the herd during an outbreak [48]. The availability of pigs from the same age between 6 and 10 weeks was therefore set as priority.

The in vivo experiment was divided into three runs with run 1 = 3, run 2 = 4 and run 3 = 5 animals, respectively. In total there were eight females, two males and two castrated male pigs used in the experiment. Six pigs were used as uninfected control and six pigs were infected as described below.

The sample size in part A was not statistically calculated, as it was only used for technical adjustment. The sample size in part B was calculated with 6 animals per group and two animals for replacement. For statistical calculation, the following parameters were used: Error 1st type, α = 5%, error 2nd type, β = 20% (power 80%), relevant difference to be detected: 2% pO_2_. To calculate the number of animals the software GraphPad StatMate 2.00 was used.

The total number of animals used in this study was 20.

If it was impossible to get an excess to CSF, the experiment was stopped and the animal euthanized due to animal well fare reasons (one animal in this study).

### Catheter position and computed tomography scan

In order to establish good puncture results as fast as possible regarding the CSF at the atlantooccipital joint in living animals, the procedure of finding the correct puncture site was trained on half-carcasses at the Institute for Food Quality and Food Safety (University of Veterinary Medicine Hannover, Hannover, Germany) and on dead pigs with a body weight of approximately 13 kg. The position of an inserted epidural catheter inside the subarachnoid space (equal to the position of the needle in the CSF compartment at the atlantooccipital joint) was documented by computed tomography (CT; Philips Brilliance 64) scan. A picture and video were generated with standard settings in the CT software.

### Training of staff in puncture technique

The training of the personnel was carried out in previously published animal trial [18]. At least five animals are required to reliably perform the puncture. In the training, explicit emphasis was placed on the correct positioning of the animal, the safe recognition of the puncture site by bending the head and palpating the *Articulatio atlantooccipitalis* (atlantooccipital joint). Equally important are the correct stitch angle and stitch depth as well as hitting the center of the back. The goal of the exercises was to obtain a sterile, blood-free CSF sample in the shortest possible time.

### Method of euthanasia in experiment: *post mortem* pO2 determination in the CSF of healthy pigs

The pigs were anesthetized with azaperone (2 mg kg^−1^ body weight (BW), Stresnil ad us. vet., Elanco Tiergesundheit AG, Basel, Switzerland) and ketamine-hydrochloride (20 mg kg^−1^ BW, Ursotamin, 100 mg mL^−1^, Serumwerk Bernburg AG, Bernburg, Germany) intramuscularly. The injection was carried out in the *Musculus (M.) biventer cervicis* near the base of the ear with a 21 G cannula (Sterican 0.80 × 40 mm, B. Braun Melsungen AG, Germany). The pig was separated and therefore protected from the other pigs to allow a gentle start of the anesthesia. The depth of anesthesia was proven by observation of a trained veterinarian. Then the pig was transported in a separate room for further examination and euthanasia. The neck area was shaved around the *Articulatio atlantooccipitalis* (atlantooccipital joint). By dorsoventral movement of the neck, the point of most flexibility was identified and marked with a laterolateral transverse line with a permanent marker. Furthermore, a median sagittal line was drawn. With this, an intersection point was found (Fig. 2c). The animal was placed in lateral recumbency and the neck was bent at maximum towards the rib cage and fixed by tension straps. Afterwards, the head was aligned exactly horizontal to the table with ropes. The puncture site was aseptically prepared. The pigs were euthanized intravenously via the auricular vein (*Vena auricularis*) with T 61® (3–4 mL/50 kg BW, Intervet Deutschland GmbH, Unterschleißheim, Germany) during anesthesia. The death of the pig was determined by a trained veterinarian and confirmed by absence of heartbeat and reflexes. The procedure of euthanasia followed the recommendations for euthanasia of experimental animals [49].

### *Post mortem* pO2 determination in the CSF of healthy pigs

After determination of death (absence of heartbeat), a Spinocan® epidural needle (type Tuohy, 1.30 × 88 mm, G 18 × 3 1/2"; B. Braun Melsungen AG) was inserted in a rostral direction approximately 1 cm caudal of the intersection point. After detecting a slight resistance by penetrating the arachnoid membrane, the stylet was removed. Clear CSF dripped out of the needle hub if the subarachnoid cavity was punctured. If no CSF was observed or in case of blood contamination, the needle was moved slightly until clear CSF dripped out. After clear CSF had been observed, the needle was immediately connected to the 10-cm-long tube of a three-way valve (Discofix®-C; B. Braun Melsungen AG). The FTC oxygen sensor (FTC-PSt7; PreSens Precision Sensing GmbH, Regensburg, Germany) was then connected in series to this three-way valve. A second three-way valve with a syringe was connected to this combination. Fresh CSF was drawn into the entire system via suction from the syringe. Fresh CSF aspirates were drawn after five and ten minutes to determine the pO_2_ of the CSF at different time-points *post mortem*. By supplying the CSF sample to the sensors using the three-way valves, the pO_2_ measurements could be performed anaerobically, although the syringe could be removed with the CSF aspirate for further examinations (see Additional file 2: Figure S2 for detailed information).

The measuring sensors were covered with aluminium foil to keep the temperature constant and to protect the sensors from light. The sample was measured at intervals over a time-span of maximum ten minutes *post mortem*. The value one-minute after taking CSF over the sensor was used for statistical analysis to achieve one-step sensor equilibration in the case of the first taken value. After five minutes, fresh CSF was drawn over the sensor and the value one minute later was used for statistical purposes (two-step sensor equilibration). Since the ambient pressure and the temperature of the medium are essential parameters to calculate the oxygen level in liquids, the current ambient pressure of the room was determined (measured with Fisherbrand™ Traceable Digital Barometer; Thermo Fisher Scientific Inc, Waltham, MA, USA) and used to calculate the oxygen level. Since an exact temperature measurement of the CSF proved impossible, the rectal body temperature was measured and taken as the basis for the oxygen calculation.

### *Streptococcus suis* growth conditions

In this study, *Streptococcus (S.) suis* cps type 2 strain 10 (*S. suis*) was used. This strain has been shown to be highly virulent in experimental infections of pigs [50–52]. *S. suis* was grown on Columbia agar plate with 7% sheep blood (Oxoid Deutschland GmbH, Wesel, Germany) and incubated for 20–24 h at 37 °C. To prepare the infection inoculum, 10 mL Tryptic Soy Broth without dextrose (TSB) (Becton, Dickinson and Company, Sparks Glencoe, MD, USA) were filled in T405-Cultubes™ (Simport® Scientific Inc., Belœil, Canada) with air exchange and two freshly grown *S. suis* colonies were added. The culture was incubated for ten hours (37 °C / 5% CO_2_). A 1:100 dilution was created in an Erlenmeyer flask with preheated TSB and incubated for three-four hours (37 °C / 5% CO_2_) to the late exponential growth phase reflected by an OD_600nm_ of 0.3 ± 0.02. Then, 40 mL of the bacterial culture were transferred to a 50 mL Falcon Tube (SARSTEDT AG & Co. KG, Nümbrecht, Germany) and was centrifuged at 4816 g for ten minutes at room temperature. The supernatant was discarded, and the pellet was resuspended in 3 mL sterile phosphate-buffered saline (PBS). From this suspension a 1:10 dilution was made, and 1 mL thereof was intravenously injected into pigs as described below. The exact infection dose for each piglet was determined by plating serious dilutions on blood agar plates and counting colonies after 20-24 h incubation at 37 °C.

### Infection of pigs with long-term anesthesia and control of physiologic parameters

Seven-to nine-week-old German Landrace pigs (weaned at four to five weeks of age) were anesthetized as described above with azaperone and ketamine-hydrochloride intramuscularly and were intravenously (*Vena auricularis*) inoculated with 1 mL PBS containing 2.5–3.7 × 10^8^ CFU of *S. suis* infection inoculum (as described above) or 1 mL PBS in case of the control animals.

After the infection, the animals were housed separately for the next eight-12 h. Using a scoring system, eight hours *post infection* the pigs were checked every hour for severe symptoms. If a score of 25 was reached earlier than 12 h *post infection*, the second anesthesia started immediately due to animal welfare reasons. Without reaching the maximum score, pigs were anaesthetized again after 12 h. Anesthesia was induced with ketamine 20 mg kg^−1^ BW), azaperone (2 mg kg^−1^ BW) and atropine (0.06 mg kg^−1^ BW, Atropinsulfat B.Braun 0.5 mg mL^−1^ injection solution, B. Braun Melsungen AG).

Venous access was established by an indwelling venous catheter (Vasovet Braunüle, 20G/22G; B. Braun Melsungen AG) either in an ear vein, the cephalic or lateral saphenous vein. Propofol (1–2 mg kg^−1^ BW; Narcofol® 10 mg/mL BW, CP Pharma, Handelsgesellschaft mbH, Burgdorf, Germany) was administered to achieve endotracheal intubation. After topical anesthesia with tetracaine spray (Gingicain®D, 754 mg/65 g, Sanofi-Aventis Deutschland GmbH, Frankfurt am Main, Germany), the pigs were endotracheally intubated under visual control with a cuffed endotracheal tube (Portex 100/150/XX ID 5–6; Smiths Medical, Inc., MN, USA).

The endotracheal tube was connected to a circle breathing system (DRAEGER Titus; Drägerwerk AG & Co. KGaA., Lübeck, Germany) and volume controlled mechanical ventilation (DRAEGER Ventilog C; Drägerwerk AG & Co. KGaA., Lübeck, Germany) with a tidal volume of 10–16 mL kg^−1^ and a pressure limitation of 20 mbar without PEPP was immediately started. The respiratory rate was set at 20 breath min^−1^ and adjusted to maintain eucapnia.

Anesthesia was maintained with isoflurane (Isoflurane CP 1 mL/mL, 250 mL, CP-pharma GmbH) in air. To avoid hyperoxia and hypoxia, oxygen was supplemented to adjust FiO_2_ as needed to maintain an arterial partial pressure of oxygen of 80 to 110 mmHg. A constant infusion rate of ketamine (1 mg kg^−1^ h^−1^; Ketamin® 100 mg/mL, CP Pharma GmbH) and dexmedetomidine (2 µg/kg/h; Cepedex® 0.5 mg mL^−1^, CP-Pharma GmbH) was administered for MAC-sparing effects. To facilitate mechanical ventilation, 0.15 mg kg^−1^ levomethadone/fenpipramide (L-Polamivet 2.5/0.125 mg mL^−1^ ®, Intervet GmbH, Unterschleißheim) was injected intravenously immediately after intubation and repeated after four hours of anesthesia. For cardiovascular support, a balanced, lactate buffered electrolyte solution (Sterofundin ® 1/1 E, B. Braun Melsungen AG) was administered at a rate of 5 mL kg^−1^ h^−1^ throughout the procedure. Additional file 9: Table S6 lists all drugs that were used for each individual animal to ensure effective anesthesia.

The depth of anesthesia was monitored by testing muscle relaxation, the eye reflexes, the interdigital withdrawal reflexes and skin stimulation with a Kelly type artery clamp. The ECG, heart rate, arterial blood pressure, peripheral oxygen saturation (SPO_2_) via a transmission pulse oximetry probe placed at the tail or a claw and the rectal temperature were continuously monitored via an anesthesia multiparameter monitor (DATEX Ohmeda Cardiocap 5, General Electric Company, Boston, MA, USA). For invasive blood pressure measurement, access was made to the femoral artery by an arterial catheter (BD Insyte-W, 22G; Becton Dickinson, Franklin Lakes, NJ, USA). The pressure was recorded via a calibrated pressure transducer (BRAUN Combitrans Monitoring set venous; B. Braun Melsungen AG) levelled and zeroed to ambient pressure at the base of the heart. End-tidal CO_2_, inspired and expired isoflurane concentrations as well as the inspired and expired (FeO_2_) fraction of oxygen were monitored via a respiratory gas monitor (Dräger PM 8050, Drägerwerk AG & Co. KGaA., Lübeck, Germany).

Arterial blood gas measurements were performed using the EPOC system (epoc® Blood Analysis System; Siemens Healthcare GmbH, Erlangen, Germany) before each pO_2_ determination in the CSF, and individually as needed to adjust FiO_2_ to maintain physiologic arterial partial pressure of oxygen. Mean arterial blood pressure was maintained above 60 mmHg by fluid infusion and inotropes or vasopressors as required.

The temperature of the animals were maintained by means of red-light heat lamps and adjustable electrical heating mats during the period of anesthesia. The initial temperature was the temperature at the time of inducting anesthesia.

### Method of euthanasia in experiment: Infection of pigs with long-term anesthesia and control of physiologic parameters

After completing the measurements, the animals were euthanized by a trained veterinarian during isoflurane anesthesia, for further examination. Therefore, the pigs were euthanized intravenously via one of the already existing venous access points (described above) via the indwelling venous catheter (Vasovet Braunüle, 20G/22G; B. Braun Melsungen AG) either in an ear vein, the cephalic or lateral saphenous vein with T 61® (3–4 mL/50 kg BW, Intervet Deutschland GmbH, Unterschleißheim, Germany) during isoflurane anesthesia. The death of the pig was determined by a trained veterinarian and confirmed by absence of heartbeat and reflexes. The procedure of euthanasia followed the recommendations for euthanasia of experimental animals [49].

### Oxygen and pH measurements in the CSF and sampling during anesthesia

After endotracheal intubation, the pigs were positioned, and the CSF access carried out as described above. The needle was connected to the 10-cm-long tube of a three-way valve (Discofix®-C; B. Braun Melsungen AG). One side was connected to a pressure transducer (Combitrans ®; B. Braun Melsungen AG) and the other side to the measurement system. Therefore, the FTC oxygen sensor (FTC-PSt7; PreSens Precision Sensing GmbH) and the pH sensor (FTC-SU-HP5-US; PreSens Precision Sensing GmbH, Regensburg, Germany) were then connected in series to the leftover connection. At the open side of the pH sensor, it was possible to collect CSF samples with a syringe by means of a second three-way valve.

After installing the measurement set-up, the three-way valve was opened in such a way that the pressure of the CSF (pCSF) was monitored permanently with the transducer. Before measuring O_2_ and the pH level inside the CSF, a six minute two-step sensor equilibration was conducted. For this purpose, the three-way valves were opened so that 1.8 mL of fresh CSF was drawn into a 2 mL syringe via the oxygen and pH sensors. Thereafter, the three-way valves were reversed so that CSF was trapped over the sensors without any air contact. For the following five minutes, the sensor probes equilibrated to the CSF (first step). The solution in the syringe was disposed of (CSF in the syringe (13 h p.i.) and the CSF- sodium chloride mixture due to rinsing procedures beforehand (16 h p.i. / 19 h p.i.)), respectively. After this first equilibration, and rinsing out the leftover sodium chloride solution, 1 mL fresh CSF was drawn into a sterile syringe as previously described before and the second one-minute equilibration step of the sensors was already performed with measuring values. During a ten-minute period, values were collected every 30 s leading to a results curve. The five-minute adaptation (from sodium chloride to CSF over the sensor) and the following one-minute equilibration (fresh CSF over the sensor for temperature and pressure compensation) were termed as the two-step equilibration. All values of pO_2_ and pH presented in main figures were taken after this two-step equilibration. All other values were analyzed and summarized in Additional file. After these measurements, the system was rinsed with 0.9% sodium chloride solution using method “III” as described in Additional file 2: Figures S4 and S5. The measurement was carried out regularly one hour after starting isoflurane anesthesia (13 h *post infection*), four hours (16 h *post infection*) and seven hours (19 h *post infection*). For every measurement, this two-step equilibration was performed. The sensors were covered with aluminium foil to keep the temperature constant and to protect the sensors from light. After each measurement, the system was rinsed with an outflow of 0.9% sodium chloride solution from the pressure transducer.

For oxygen determination in liquids, the ambient pressure and the temperature of the medium are needed. Here, we combined the prevailing air pressure (p_air_) of the room (~ 21% O_2_) (Fisherbrand™ Traceable Digital Barometer; Thermo Fisher Scientific Inc) with the CSF pressure that had been directly measured before the measurement set-up was opened for the measurement (no pressure drop caused by opening the measuring system). Since the CSF pressure (pCSF) also influences the oxygen calculation, the sum of both pressures (pAir + pCSF) was used as the final value for the pressure compensation. The rectally measured body temperature was taken as the basis for the temperature compensation in the oxygen calculation and pH determination.

At each time point (13–16-19 h p.i.), the CSF sample in the syringe was transported on ice to the laboratory for further examinations.

### Cleaning procedure of measurement system during in vivo experiment

A change in the measuring system during the experiment was not possible. Therefore, a rinsing process to decontaminate the measuring system between the measurement points was established as described in the Additional file.

### Analysis of samples from animal experiment

Blood samples were taken from the *Vena jugularis* or *Vena cava cranialis pre-infection* and from the *Arteria femoralis*, at defined time-points (0, 13, 16 and 19 h *post infection*) in lithium–heparin monovettes (SARSTEDT AG & Co. KG). Serial dilutions were plated on Columbia blood agar to determine the CFU/mL after incubation at 37 °C for 20–24 h.

During the first anesthesia, a tonsillar swab (Amies medium, SARSTEDT AG & Co. KG) was taken for bacterial examination to exclude bacterial colonization of pigs with *S. suis*.

After euthanasia and *post mortem* oxygen measurement in CSF, a necropsy was conducted. Swabs of the brain surface, the mitral valve, the pleura, the pericard and the peritoneum and organ samples of the liver, spleen, lung and tonsils as well as liquid from the carpal and tarsal joints were collected for bacteriologic examination.

All colonies suspected of *S. suis* were analyzed by multiplex PCR as previously described [53] for the specific gene profile of the infection strain.

In the CSF samples, at each time-point, the following parameters were determined: 1. Serial dilutions were plated on Columbia blood agar to determine the CFU/mL after incubation at 37 °C for 20–24 h, 2. Counting of neutrophil numbers in the Neubauer chamber, 3. Counting of neutrophils after fixation (with 4% paraformaldehyde final) analyzed by flow cytometry using the Attune® NxT Acoustic Focusing Flow Cytometer (FACS). The analysis was based on Forward Scatter (FSC; detection of cell size) and Side Scatter (SSC, detection of granularity) and analyzed using FlowJo software version (v)10.

During section swabs of the brain surface, the mitral valve, the pleura, the pericard and the peritoneum were taken for bacteriology. Furthermore, for bacteriology and histology, organ samples of the brain, liver, spleen, tonsils, heart, lung, pleura, pericard and peritoneum were collected. Samples for histology were stored immediately in 10% buffered formalin and after latest 72 h embedded in paraffin and cut into 2–4 µm sections for hematoxylin–eosin (HE) staining and histological examination. The histological screenings were scored as described [50] and furthermore are mentioned in the footnotes of the scoring table. The organ and swab samples for bacteriology examination were analyzed as described previously [18] with slight changes. Instead of a matrix-assisted laser desorption ionization time-of-flight mass spectrometry (MALDI-TOF MS), *S. suis* was identified by colony morphology on and afterwards by multiplex PCR identified as described previously [18, 53].

### Influence of blood hypoxygenation on the oxygen level in CSF

The influence of a possible hypo-oxygenation on the pO_2_ in the CSF was simulated by cardiac arrest by stopping the blood circulation, leading to an undersupply of oxygen to the organism. For this purpose, the oxygen level in the CSF was measured in a living animal directly before cardiac arrest. The animal was then euthanized. Ten minutes after verification of death, the oxygen level in the CSF was measured again.

### Calculation of oxygen pressure

The oxygen O_2_% was calculated based on 38 °C and 1000 hPa (= 750 mmHg) from the measured mmHg values. The values are presented in each figure and in the text with ≜.

### Statistical analysis

Data were analyzed using Excel 2010 and 2016 (Microsoft) and GraphPad Prism 8.1 (GraphPad Software). Normal distribution of data was verified by the Kolmogorov–Smirnov normality test (GraphPad software) prior to statistical analysis. Differences between groups were analyzed as described in the figure legends (*P < 0.05, **P < 0.01, ***P < 0.001, ****P < 0.0001). Detailed information can be found in the figure legend.

## Supplementary Information

**Additional file 1.** CT video of epidural catheter location.

**Additional file 2.** Additional figures.

**Additional file 3. Table S1.** Raw data of Fig. 1d.

**Additional file 4. Table S2.** Raw data of Fig. 3a.

**Additional file 5. Table S3.** Raw data Fig. 4d, e.

**Additional file 6. Table S4.** Analysis of various measurement cut-off's from all data in Fig 4d.

**Additional file 7. Table S5.** Individual pO2 values in CSF and blood and mean values per group.

**Additional file 8. Table S6.** Reisolation of the challenge strain from piglets after intravenous challenge with *S. suis *serotype 2.

**Additional file 9. Table S6.** Overview of drugs used in the animal experiment under isoflurane anesthesia.

## Data Availability

The authors confirm that the data supporting the findings of this study are available within the article or its Additional file. Raw data were generated at University of Veterinary Medicine Hannover, Department of Biochemistry. Derived data supporting the findings of this study are available from the corresponding authors NdB and MvKB on request.
