# Supplementary material for: In vivo oxygen measurement in cerebrospinal fluid of pigs to determine physiologic and pathophysiologic oxygen values during CNS infections
Source: BMC Neurosci. 2021 Jun 28;22:45. doi: 10.1186/s12868-021-00648-x (PMC8240281; doi:10.1186/s12868-021-00648-x)
Supplement: Supplementary file 2 — Additional file 2. Additional figures. [file 12868_2021_648_MOESM2_ESM.docx]

**Supplemental material**

***In vivo* oxygen measurement in cerebrospinal fluid of pigs to determine physiological and pathophysiological oxygen values during CNS infections**

Nicole de Buhr*^1,2^, Alexander Martens^1,2^, Marita Meurer^1,2^, Marta C. Bonilla^1,2^, Franz Söbbeler^3^, Lara Twele^4^, Stephan Neudeck^4^, Michael Wendt^5^, Andreas Beineke^6^, Sabine Kästner^4^, Maren von Köckritz-Blickwede*^1,2^

1 University of Veterinary Medicine Hannover, Department of Biochemistry, Hannover, Germany

2 University of Veterinary Medicine Hannover, Research Center for Emerging Infections and Zoonoses (RIZ), Hannover, Germany

3 University of Veterinary Medicine Hannover, Small Animal Clinic, Hannover, Germany

4 University of Veterinary Medicine Hannover, Clinic for Horses, Hannover, Germany

5 University of Veterinary Medicine Hannover, Clinic for Swine and Small Ruminants, Forensic Medicine and Ambulatory Service, Hannover, Germany

6 University of Veterinary Medicine Hannover, Department of Pathology, Hannover, Germany

*Correspondence: Nicole Buhr (nicole.de.buhr@tiho-hannover.de) or Maren von Köckritz-Blickwede (maren.von.koeckritz-blickwede@tiho-hannover.de)

**Supplemental figures**

**
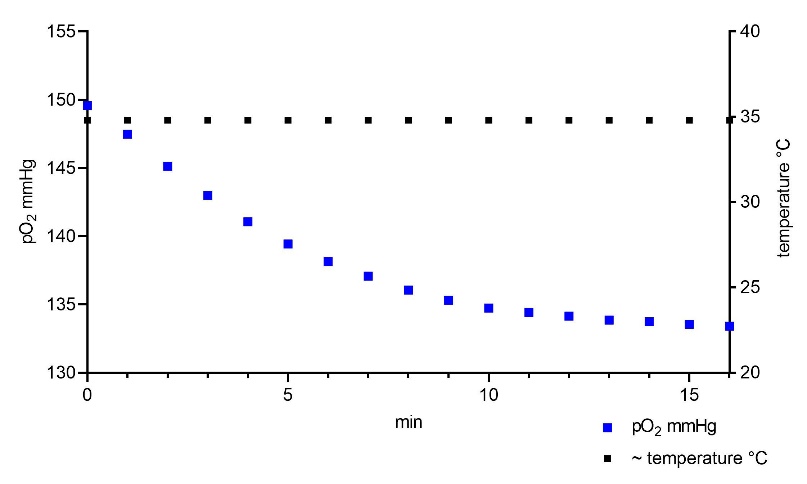
**

**Figure S1 Influence of decreasing medium temperature on the pO_2_ determination.**

The influence of differences between actual and target temperature values were analysed *in vitro*. Therefore, the pO_2_ was determined in a cooling water sample and the influence of different compensation temperatures was examined for the calculation. Temperature in 100 mL water was determined (0 min = 34.8°C). At the start of the experiment, water from the bottle was sucked into the measurement system. The black dots depict the temperature that was entered in the software for calculating pO_2_. The resulting curves of the pO_2_ over time show the influence of increasing differences between the actual temperature of the sample over the sensor (drops over time) and the temperature used for the calculation (remains constant). A temperature adjustment of 10°C brought the constant pO_2_ values back to 150 mmHg, indicating that after 17 minutes the fluid inside the measurement system had adapted to the room temperature of 24.8°C.

**
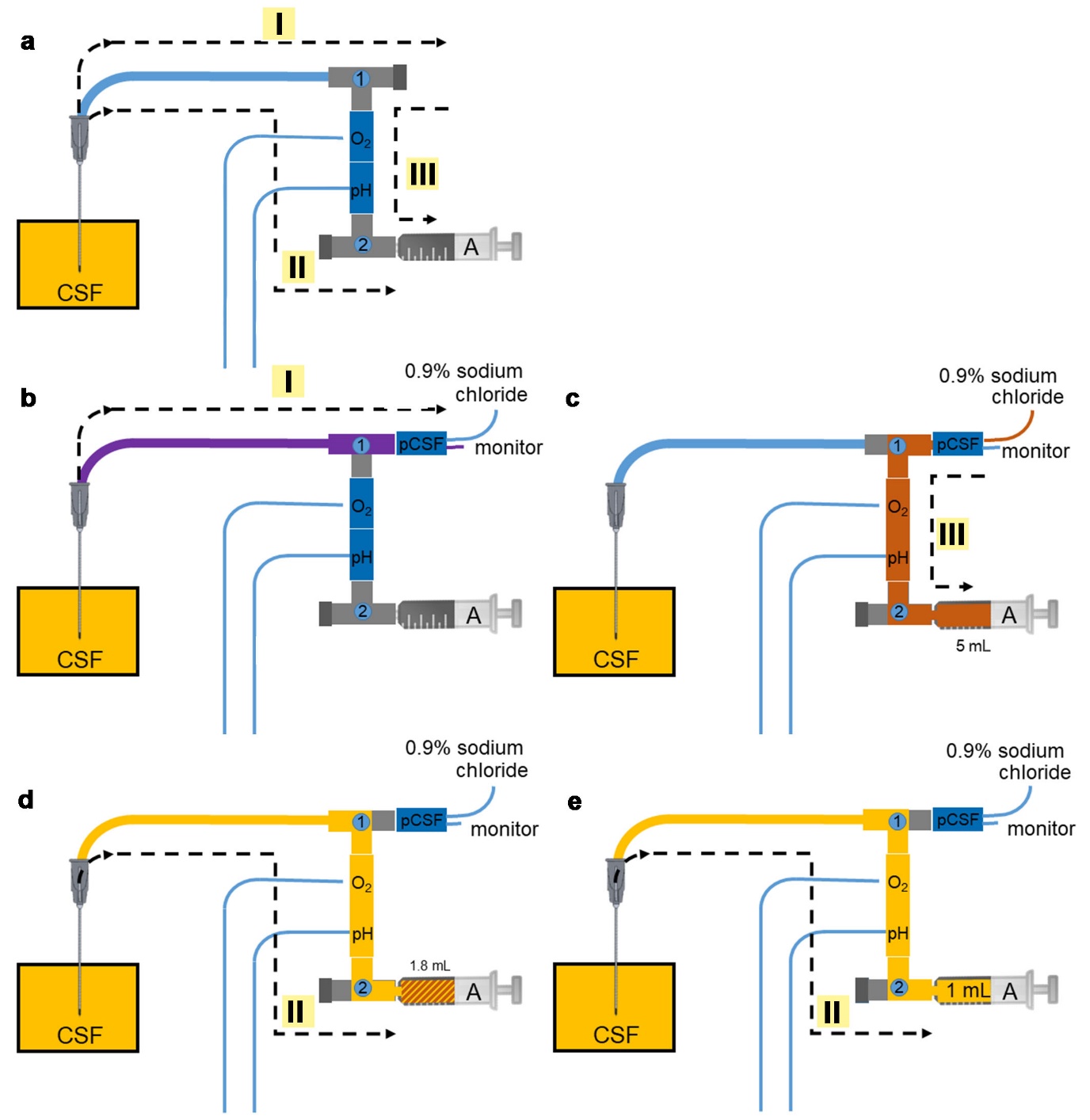
Figure S2 Scheme of the measurement setup and different fluid circuits.**

The scheme of the measurement system is equivalent to Figure 2b. A combination of a flexible tube, three-way valves, the measuring sensors, and a syringe was attached to a spinal needle inserted into the CSF compartment. Three fluid circuits, formed by combining the three-way valves settings, were used during the experiment (I-III). Furthermore, syringe A was exchanged (d and e). a) All possible circuits are presented. For the *in vivo* measurements, the continuous pressure measurement of the CSF was performed via circuit I. Circuit II represents the direct connection between the CSF compartment and the sample syringe A. Circuit III depicts the possibility of rinsing some segments (see Figure S4). By aspirating syringe A, CSF can be drawn into the measuring system without air contact. In addition, syringe A can be used to obtain CSF samples for further examinations. b) pCSF circuit, c) rinsing circuit, d) CSF measurement pre-equilibration step, e) CSF *in vivo* measurement and sampling in clean syringe A.

**
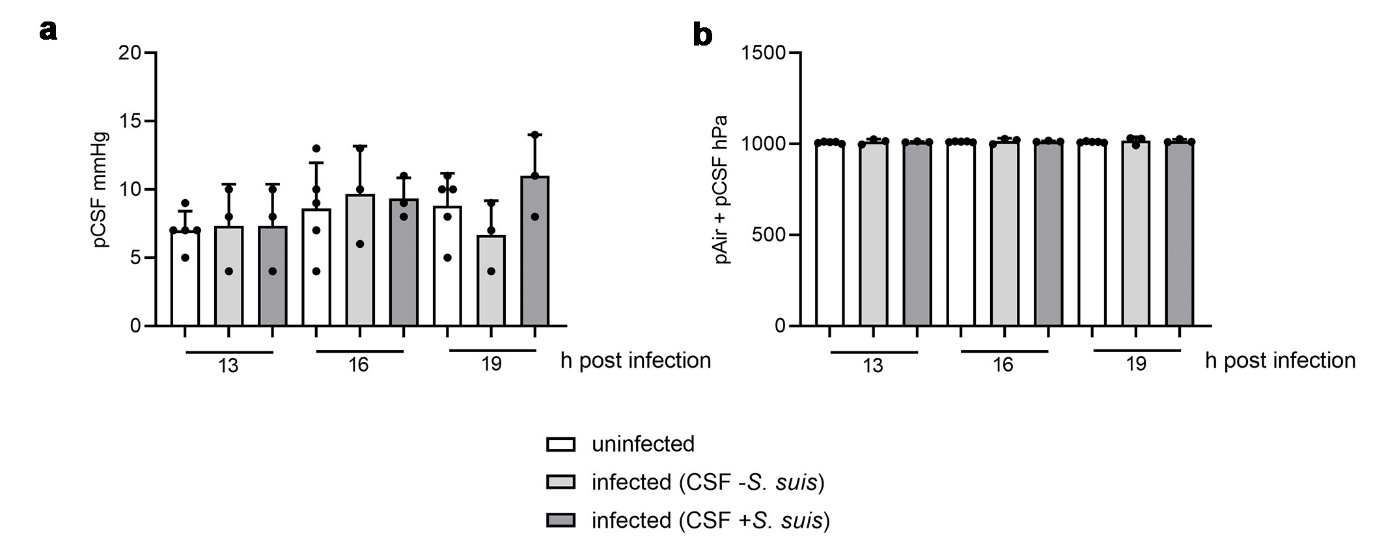
**

**Figure S3 pCSF and total pressure (pAir + pCSF) used for pO_2_ calculation.**

a) The pCSF was measured inside the CSF compartment with the measurement system presented in Figure 2b and S2 with a pressure transducer. No significant differences were detected between all time-points and groups. b) The air pressure was measured with a traceable digital barometer and the total pressure was calculated (pAir + pCSF) and used for the pO_2_ calculation.

In all graphs error bars are presented with ± SD. Each dot represents one animal. At each time-point, a Kruskal-Wallis test was performed in each group and at each time-point.

**
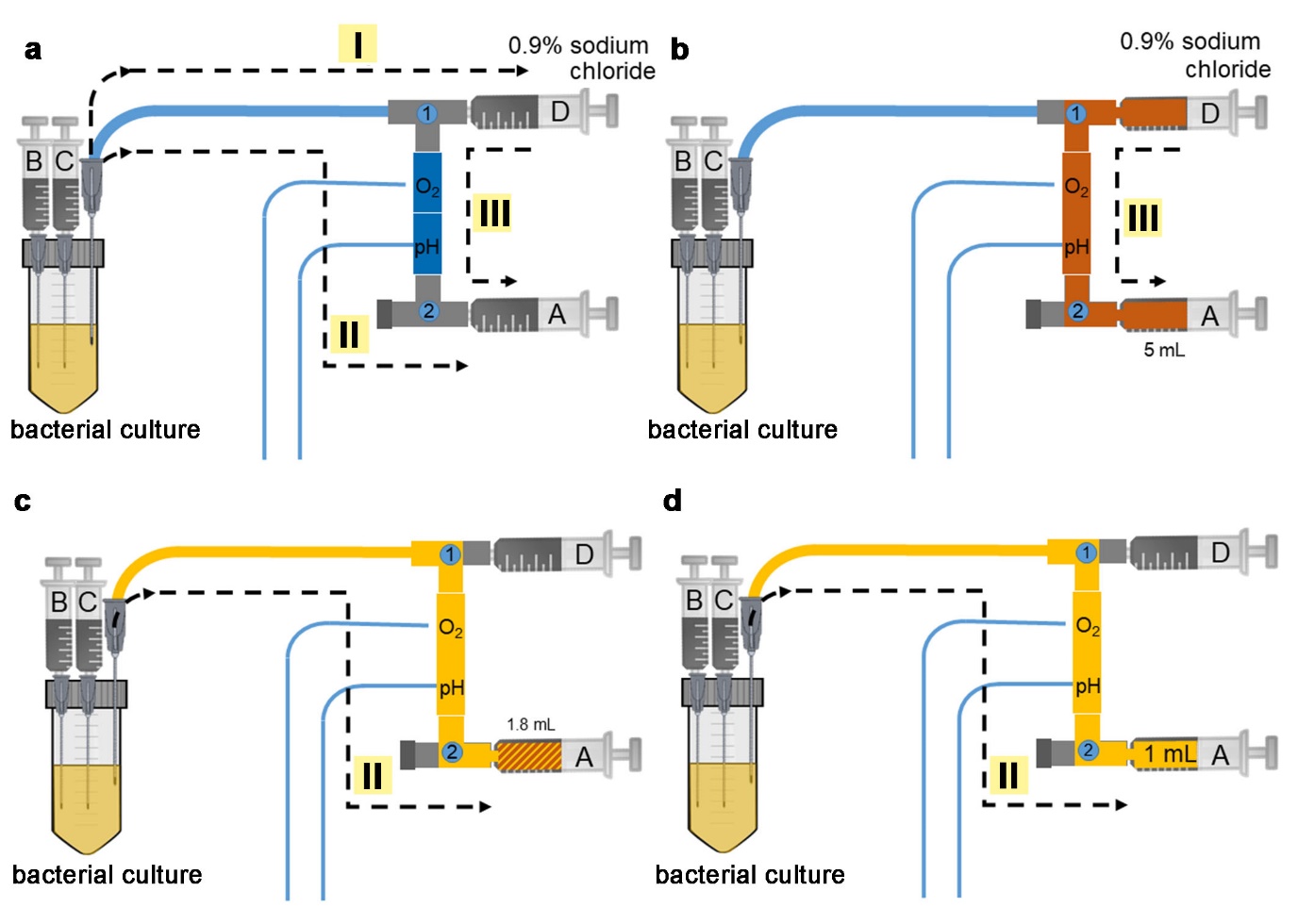
**

**Figure S4: Scheme of the cleaning simulation *in vitro* over a seven-hour period**

A combination of a flexible tube, three-way valves, the measuring sensors and a syringe was connected to a spinal needle inserted into a liquid bacterial culture. Syringe B was used to mix the bacterial culture before a sample was drawn to syringe C as CSF source equivalent. a) This *in vitro* setup simulates the *in vivo* situation and has the same three fluid circuits (I-III) as described in Figure S2. b) This overview shows rinsing with sodium chloride highlighted in orange (circuit III). c) Before taking each measurement, an equilibration of the sensors was conducted with fresh liquid via circuit II. A total volume of 1.8 mL was needed to fill the entire circuit II with fresh liquid. (orange/yellow in syringe A = CSF mixed with sodium chloride) d) This scheme shows the 1 mL CSF sample collection via circuit II in a clean syringe. The cleaning simulation run during the same time frame period as the *in vivo* experiment. At all steps of sample collection (1h, 4h and 7h), samples were taken with syringe A and the CFU/mL was determined to test whether the rinsing procedure was efficient and no bacteria were growing during the experiment *in vivo* inside the system. The sensor-syringe combination was duplicated in the experiment (not shown in the scheme). Data are presented in Figure S5.

**
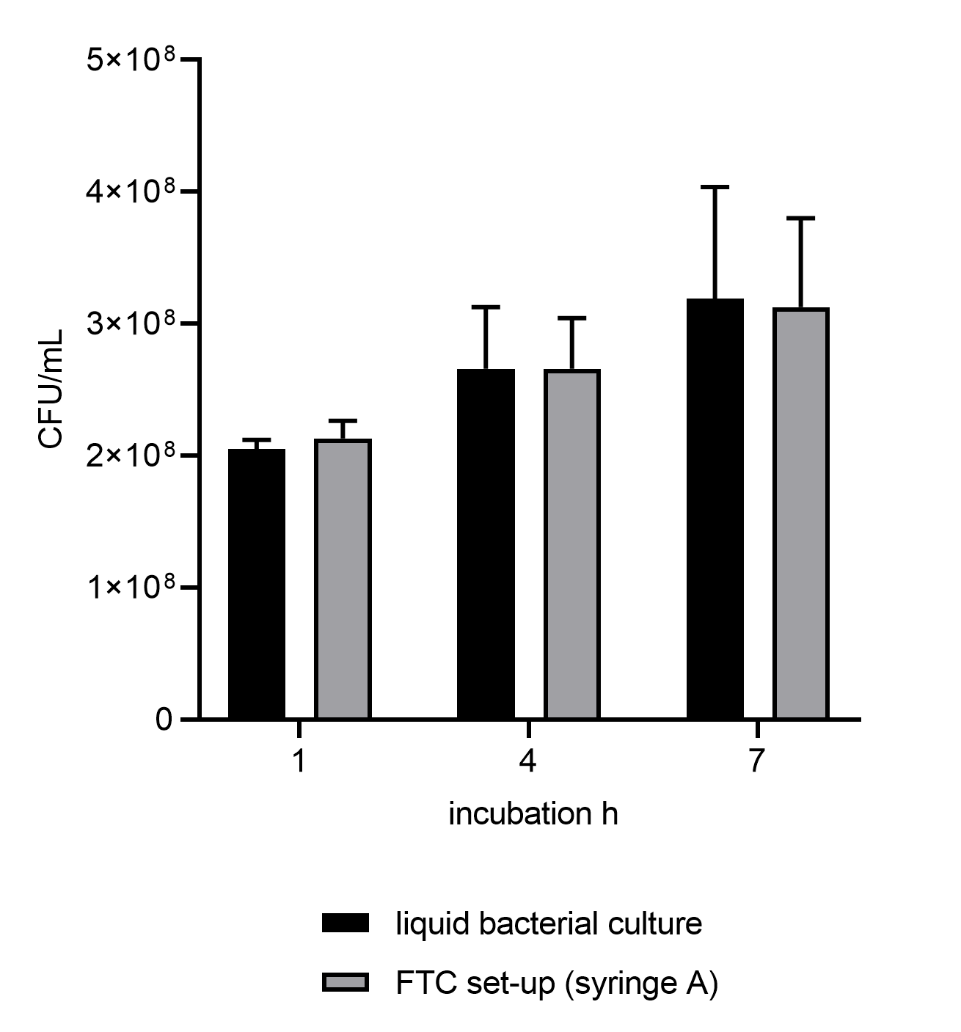
Figure S5: Results of the cleaning simulation *in vitro* over a seven-hour period.**

The CFU/mL was determined by plating the liquid bacterial culture (CSF source equivalent = falcon tube Figure S4) and the sample in syringe A (sample CSF equivalent) that was running through the FTC circuit (circuit II) after the cleaning process as described in Figure S4. The CFU/mL was comparable between the liquid bacterial culture and FTC set-up, and therefore the cleaning procedure of the system by flushing was successful. The mean ± SD is presented (n =2 with in total n= 4 FTC set-up analysis).

**
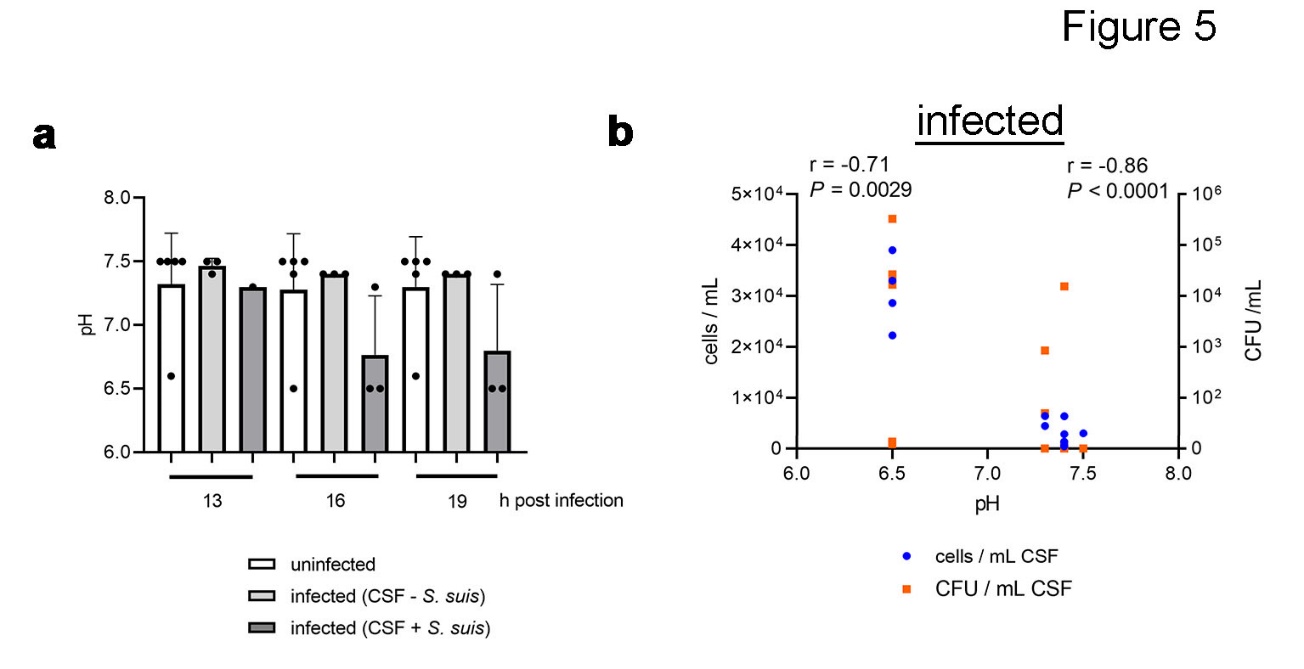
**

**Figure S6 pH level inside the CSF of infected and uninfected pigs.**

All data were collected as described in figure 2. a) The pH inside the CSF compartment was measured at all time-points without significant differences in the three groups (each symbol reflects one animal; the ANOVA table was calculated for each time point). Error bars are presented with ± SD. b). During the onset of meningitis, a significant negative correlation between the pH and cells inside the CSF (*r* and *P* values left side) and between the pH and bacteria inside the CSF (*r* and *P* values right side) was identified. The values were calculated from all parameters from all infected animals and all time-points with a two-tailed nonparametric Spearman r correlation calculation. Each point represents at least one animal. As some values were similar, some points represent more than one animal. In total, n=16 was used for the correlation calculation.

**
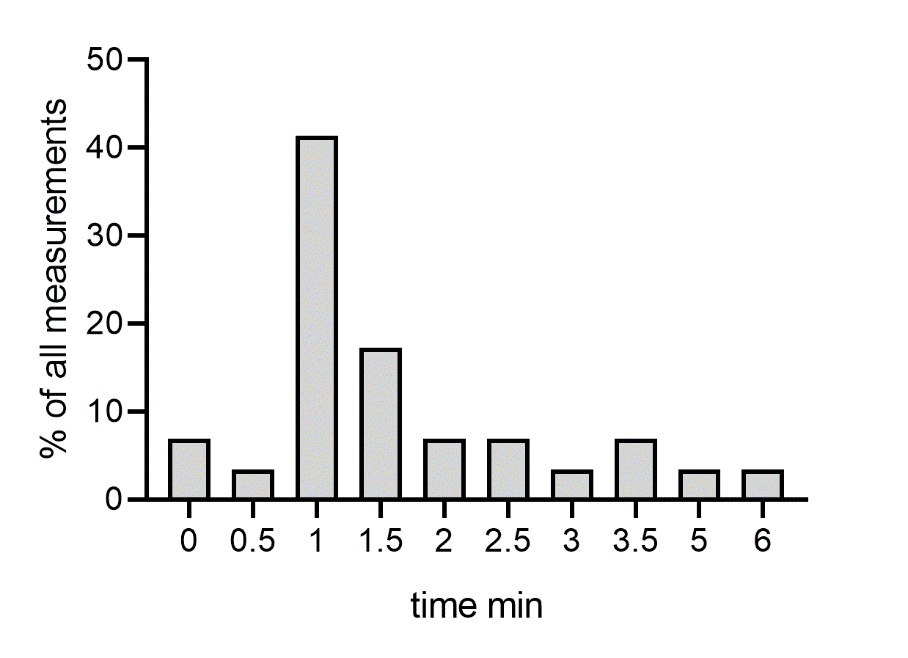
**

**Figure S7: In more than 40 % of all pO_2_ measurements, the slope cut-off value occurred at one minute.**

The slope value is the value of the oxygen curve at which a positive slope (m) of ≤ 0.8 occurred for the first time (see supplemental tables 1-3). Most of the time, this "slope value" manifests itself as a "curve minimum". In 41% of the measured values, the slope value occurred after 1 at minute (all values from figure 4d). No slope value could be given in two measurements because the slope value was always higher than the defined value of 0.8.

**Supplemental Methods**

**Influence of decreasing medium temperature on the pO_2_ determination**

According to the measurement set-up, a pO_2_ determination was performed with water at a certain temperature to investigate the influence of changing medium temperature during measurement procedures.

Therefore, a volume of 80 mL tap water was filled into a bottle (Simax Clear Graduated Lab Bottle, 100 mL) and the water temperature was determined by infrared thermography (Uni-T Pro Professional IR Thermometer UT309C). A certain volume in the bottle was aspirated into the measurement set-up described for CSF measurement in the online methods, and a continuous pO_2_ determination was carried out at 30-second intervals for a total of 17 minutes. The ambient barometric pressure (1021 hPa) was monitored and stayed stable over time. The initial temperature of the water in the bottle was taken as a basis for the pO_2_ determination using the oxygen sensor software (34.8°C). While taking the measurements, the water sample inside the sensors cooled down and the actual temperature increasingly deviated from the temperature specified for the calculation. This led to inaccurate pO_2_ determinations. After 17 minutes had elapsed, the water temperature in the bottle was taken again and these new data were adjusted in the software calculation (room temperature =24.8 °C). This led to changing and more adjusted measurement results for the sensor probes. The exact temperature of the sensor could not be determined. However, it can be assumed that small volumes in the sensor cool down faster than 100 mL inside a glass bottle. This also led to a changed pO_2_ determination.

The last two measurement results at the new temperature were taken, with the entered air pressure being slightly adjusted down to 1019 hPa.

**Cleaning the measurement set-up from bacterial contamination**

To ensure that no contamination occurred during successive sample measurements in the subsequent *in vivo* tests under bacterial load, a corresponding rinsing process with the measurement setup was simulated in the laboratory. An overview of the experimental set-up is presented in Figure S2. A tube with *S. suis* suspension was used and represented the bacterially contaminated CSF compartment *in vivo*. Syringe A simulated the CSF sampling point *in vivo*, whereas syringe D replaced the connection of the pressure transducer with its sodium chloride solution outflow. Syringe B was used to mix the bacteria, syringe C was used to directly aspirate the *S. suis* suspension out of the primary source.

For the simulation, the measurement setup was first moistened by rinsing with 0.9% sodium chloride solution one hour before the start of the examination, and simulated the first one-hour phase *in vivo* after the needle had been successfully inserted into the CSF space (path III).

Five minutes before the following step, a total volume of 1.8 mL (1.3 mL (connected components) + 0.5 mL safety addition) was aspirated into syringe A (path II). This removed the sodium chloride volume and filled the measurement components with *S. suis* solution from the tube. Additionally, the first equilibration step with the sample and the sensor matrix was performed. The aspirate in syringe A, which consisted of a mixture of sodium chloride and bacterial solution was discarded.

In the following step, the first measuring point was simulated where different parameters *in vivo* were determined. A total of 1 mL suspension was aspirated into a new syringe at position A (*in vivo* = start of second step of two-step equilibration, and the sample taken for laboratory analysis) via circuit II and in parallel, 1 mL into syringe C. The bacterial CFU from both syringes were compared. Therefore, serious dilutions were plated on blood agar plates to determine the CFU/mL. A volume of 1 mL of new *S. suis* suspension was aspirated into a new syringe at position D. Both syringes contained comparative samples, which could provide information about possible impurities in the measuring circuit II.

After 10 minutes, the components were rinsed with 5 mL NaCl solution (path III) into a new syringe at position A. The syringe was again discarded. This step simulated the rinsing procedure with sodium chloride from the pressure transducer *in vivo*. The next simulation step followed three hours later.

Five minutes before the following step, again, a total volume of 1.8 mL was aspirated into syringe A (path b). This again removed the sodium chloride volume and filled the measurement components with *S. suis* solution. Here, too, the sensor matrix was additionally equilibrated to the *S. suis* solution. The aspirate in syringe A was discarded.

In the following step, the next measuring point was simulated *in vivo*. For this, 1 mL suspension was again aspirated into a new syringe at position A (path b) and in parallel 1 mL into syringe C. The bacterial CFU from both syringes was compared.

For the last *in vivo* measurement, the rinsing procedure and the following CFU determination from syringes A and C was repeated as previously described.

The *S. suis* solution in the tube was always homogenized by syringe B before being used in the aspirations.

**Determination of pO_2_ values with the Presens software**

The supplemental tables 1-3 present all the data that were entered in the software (temperature, pressure: pAir and pCSF). Furthermore, for each measurement, we showed all measured values (every 30 sec). We calculated different mean values for pO_2_ CSF and calculated a slope value. The slope value is the value of the oxygen curve at which a positive slope (m) of ≤ 0.8 occurred for the first time. For the main experiment (infected pigs compared to uninfected pigs), we performed a statistical analysis of various measurement cut-offs (supplemental table 4). An overview at which time point the slope value was present during the measurement is presented in Figure S7.
