# Supplementary material for: In vivo oxygen measurement in cerebrospinal fluid of pigs to determine physiologic and pathophysiologic oxygen values during CNS infections
Source: BMC Neurosci. 2021 Jun 28;22:45. doi: 10.1186/s12868-021-00648-x (PMC8240281; doi:10.1186/s12868-021-00648-x)

Supplemental table 1: raw data of figure 1d

T3

6 min post mortem

Presens	pO_2 CSF [mmHg]	mean	n	slope	pAir =baro [hPa]	pCSF [mmHg]	temp. [°C]	pH
min								
0,0	88,41			-19,92				
0,5	78,46	83,43	2	-13,72				
1,0	71,59	79,49	3	-8,68				
1,5	67,25			-5,11				
2,0	64,70	74,08	5	-3,19				
2,5	63,11			-1,86				
3,0	62,18			-1,12				
3,5	61,61			-0,63				
4,0	61,30	68,73	9	-0,15				
4,5	61,23			0,13				
5,0	61,29			0,48	1002		38,4	7,4
5,5	61,53			0,42				
6,0	61,74	66,49	13					
6,5								
7,0								
7,5								
8,0								
8,5								
9,0								
9,5								
10,0								

10 min post mortem

Presens	pO_2 CSF [mmHg]	mean	n	slope	pAir =baro [hPa]	pCSF [mmHg]	temp. [°C]	pH
min								
0,0	60,42			-75,18				
0,5	22,84	41,63	2	-22,29				
1,0	11,69	31,65	3	-0,03				
1,5	11,67			1,02				
2,0	12,19	23,76	5	1,35				
2,5	12,86			2,87				
3,0	14,30							
3,5								
4,0								
4,5								
5,0					1002		38,4	7,3
5,5								
6,0								
6,5								
7,0								
7,5								
8,0								
8,5								
9,0								
9,5								
10,0								

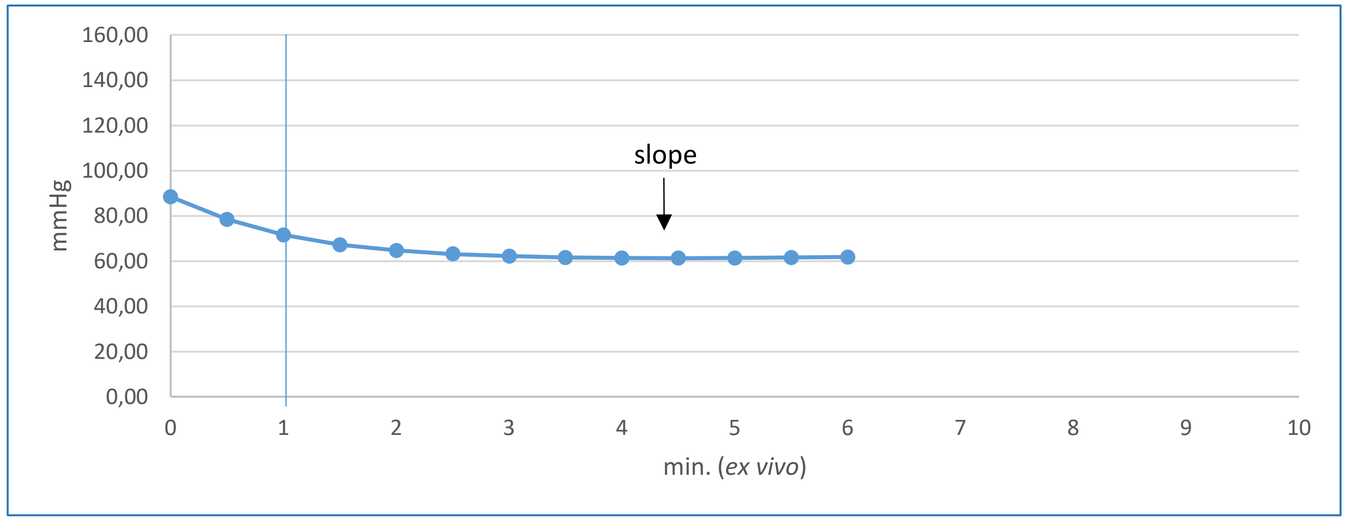

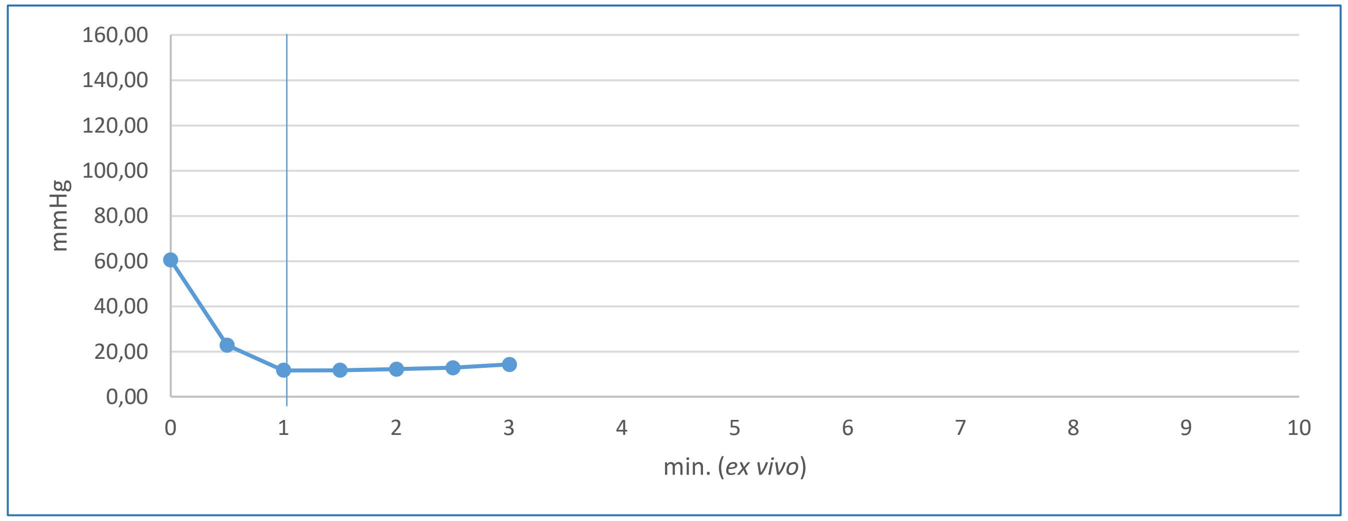

Supplemental table 1: raw data of figure 1d

T4

5 min post mortem

Presens min	pO_2 CSF [mmHg]	mean	n	slope	pAir =baro [hPa]	pCSF [mmHg]	temp. [°C]	pH
0,0	71,79			-8,12				
0,5	67,73	69,76	2	-4,67				
1,0	65,40	68,31	3	-3,04				
1,5	63,88			-1,93				
2,0	62,91	66,34	5	-1,10				
2,5	62,36			-0,53				
3,0	62,10			-0,13				
3,5	62,03			0,33				
4,0	62,20	64,49	9	0,49				
4,5	62,45			0,73				
5,0	62,81			0,78	1002		38,4	
5,5	63,20			0,80				
6,0	63,60	64,04	13	0,85				
6,5	64,02							
7,0								
7,5								
8,0								
8,5								
9,0								
9,5								
10,0								

10 min post mortem

Presens min	pO_2 CSF [mmHg]	mean	n	slope	pAir =baro [hPa]	pCSF [mmHg]	temp. [°C]	pH
0,0	62,55			-51,99				
0,5	36,55	49,55	2	-45,76				
1,0	13,67	37,59	3	-3,82				
1,5	11,76			0,31				
2,0	11,92	27,29	5					
2,5								
3,0								
3,5								
4,0								
4,5								
5,0					1002		38,4	
5,5								
6,0								
6,5								
7,0								
7,5								
8,0								
8,5								
9,0								
9,5								
10,0								

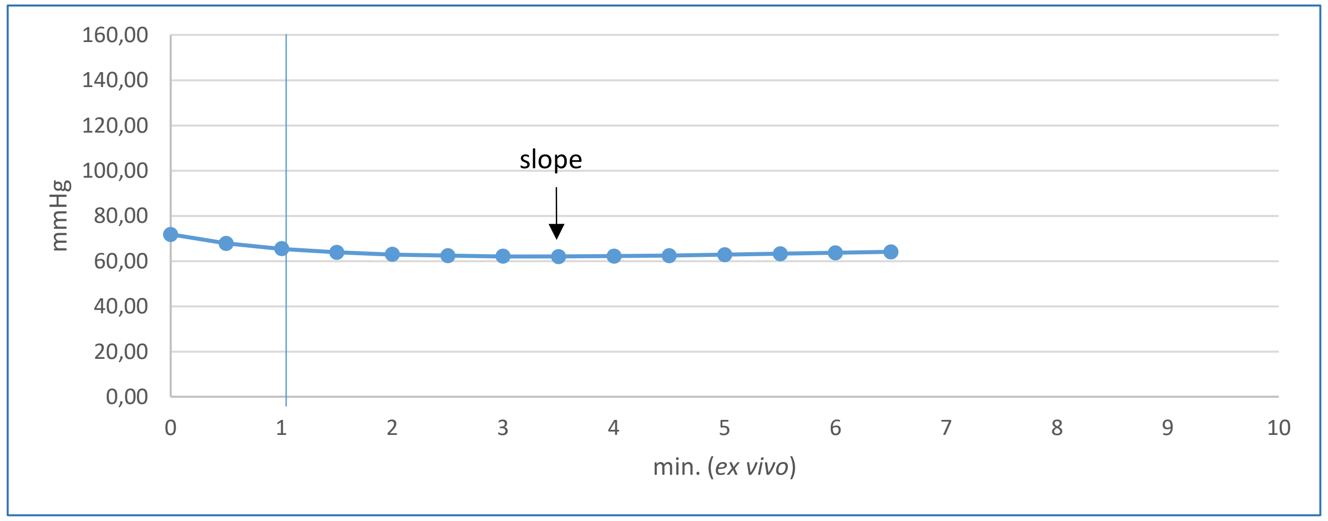

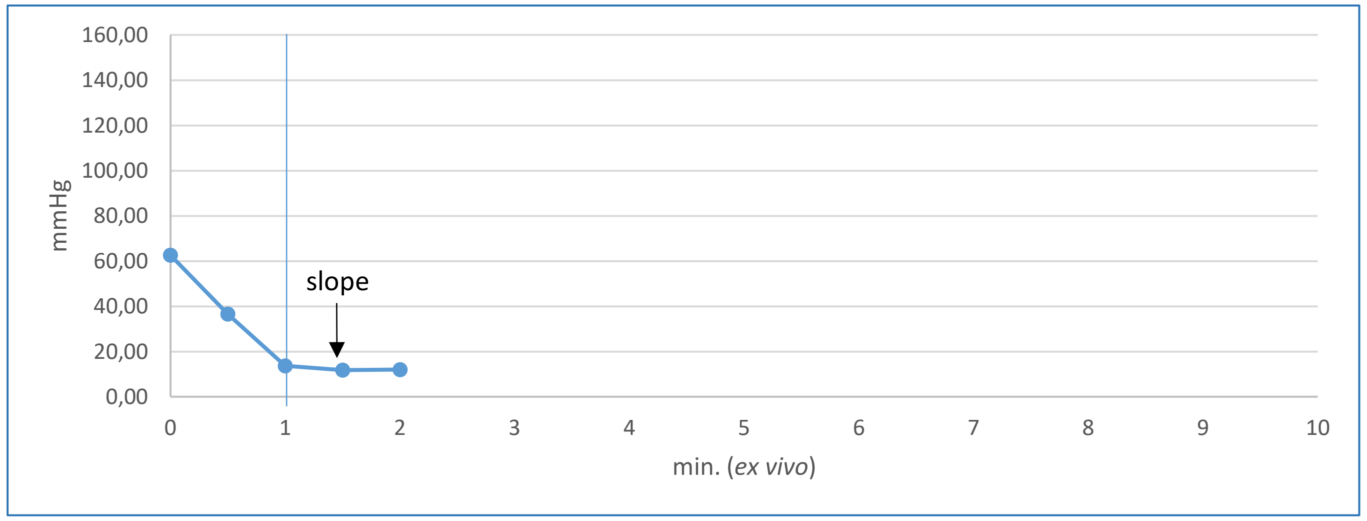

Supplemental table 1: raw data of figure 1d

T5

2 min post mortem

Presens	pO_2 CSF [mmHg]	mean	n	slope	pAir =baro [hPa]	pCSF [mmHg]	temp. [°C]	pH
min								
0,0	37,90			-6,40				
0,5	34,69	36,30	2	0,55				
1,0	34,97	35,85	3	2,23				
1,5	36,08			3,11				
2,0	37,64	36,26	5	2,91				
2,5	39,09							
3,0								
3,5								
4,0								
4,5								
5,0					1002		37,4	7,4
5,5								
6,0								
6,5								
7,0								
7,5								
8,0								
8,5								
9,0								
9,5								
10,0								

5 min post mortem

Presens	pO_2 CSF [mmHg]	mean	n	slope	pAir =baro [hPa]	pCSF [mmHg]	temp. [°C]	pH
min								
0,0	34,92			-35,49				
0,5	17,17	26,05	2	-2,26				
1,0	16,04	22,71	3	0,70				
1,5	16,39			1,86				
2,0	17,32	20,37	5	2,49				
2,5	18,57			2,82				
3,0	19,98			2,89				
3,5	21,42			2,74				
4,0	22,79	20,51	9	2,55				
4,5	24,07							
5,0					1002		37,4	7,3
5,5								
6,0								
6,5								
7,0								
7,5								
8,0								
8,5								
9,0								
9,5								
10,0								

10 min post mortem

Presens	pO_2 CSF [mmHg]	mean	n	slope	pAir =baro [hPa]	pCSF [mmHg]	temp. [°C]	pH
min								
0,0	20,43			-26,77				
0,5	7,04	13,73	2	-0,46				
1,0	6,81	11,43	3	1,10				
1,5	7,36			2,30				
2,0	8,51	10,03	5					
2,5								
3,0								
3,5								
4,0								
4,5								
5,0					1002		37,4	6,9
5,5								
6,0								
6,5								
7,0								
7,5								
8,0								
8,5								
9,0								
9,5								
10,0								

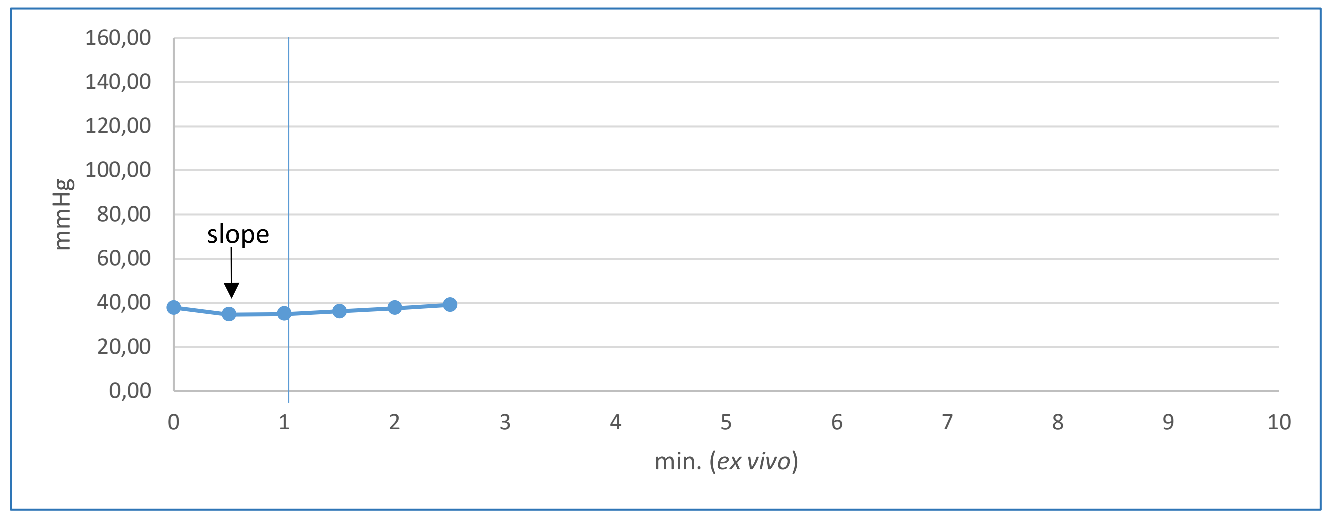

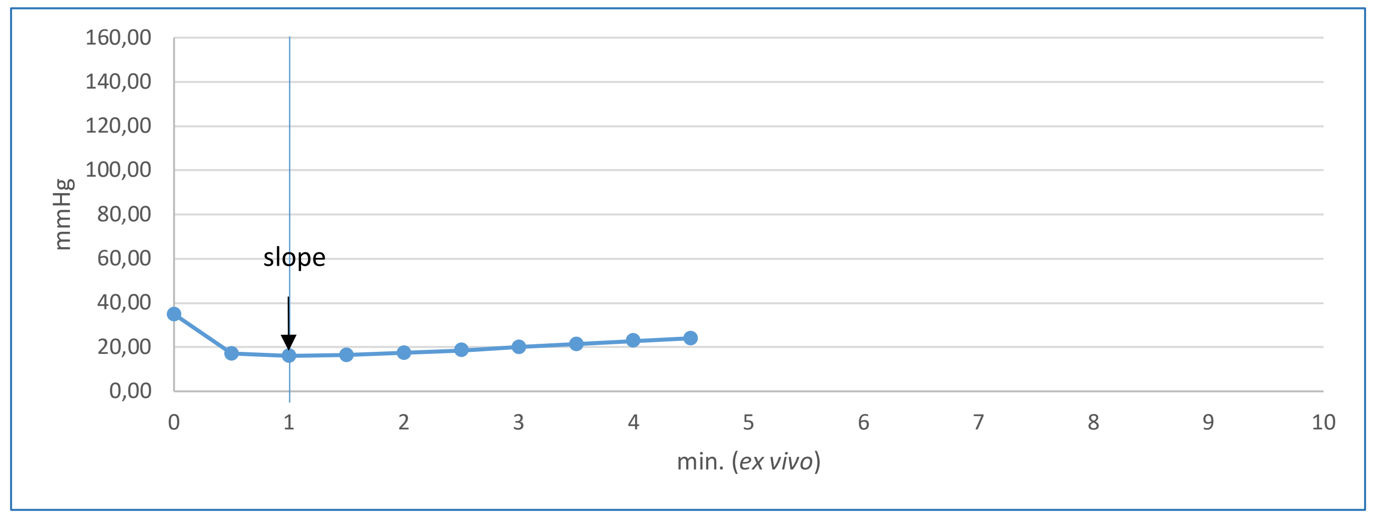

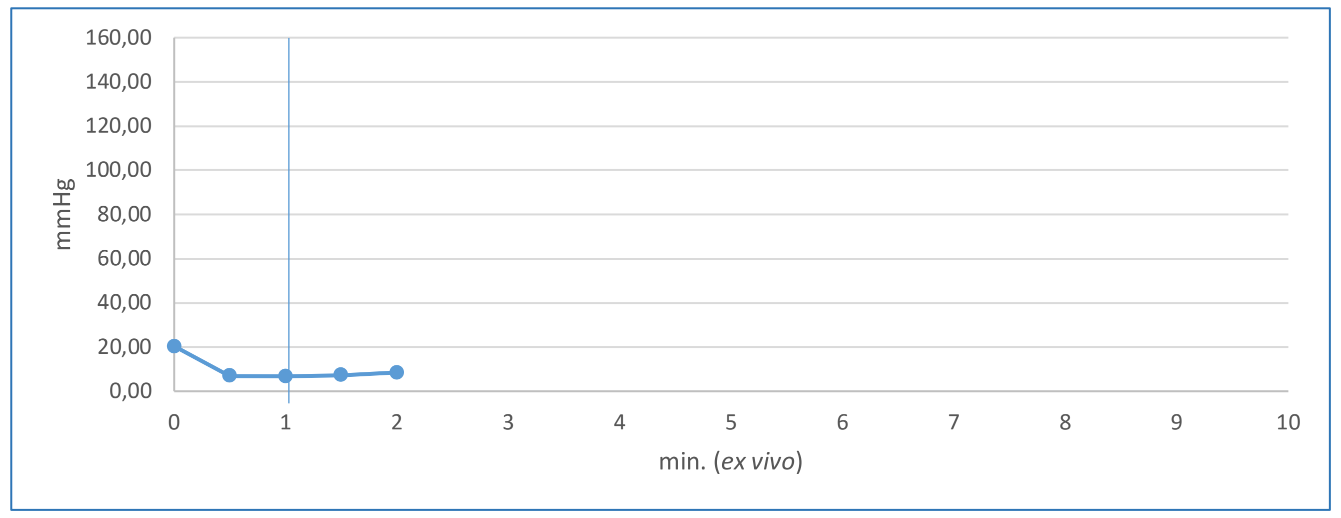

Supplemental table 1: raw data of figure 1d

T7

3 min post mortem

Presens min	pO_2 CSF [mmHg]	mean	n	slope	pAir =baro [hPa]	pCSF [mmHg]	temp. [°C]	pH
0,0	65,79			-18,46				
0,5	56,57	61,18	2	-7,03				
1,0	53,05	58,47	3	-2,54				
1,5	51,78			-0,94				
2,0	51,31	55,70	5	-0,21				
2,5	51,20			-0,06				
3,0	51,17							
3,5								
4,0								
4,5								
5,0					1010		38,5	7,4
5,5								
6,0								
6,5								
7,0								
7,5								
8,0								
8,5								
9,0								
9,5								
10,0								

5 min post mortem

Presens min	pO_2 CSF [mmHg]	mean	n	slope	pAir =baro [hPa]	pCSF [mmHg]	temp. [°C]	pH
0,0	49,59			-38,07				
0,5	30,56	40,08	2	-6,17				
1,0	27,47	35,88	3	-1,44				
1,5	26,76			0,35				
2,0	26,93	32,26	5	1,06				
2,5	27,46			1,43				
3,0	28,18			1,63				
3,5	29,00			1,66				
4,0	29,83	30,64	9					
4,5								
5,0					1010		38,5	7,4
5,5								
6,0								
6,5								
7,0								
7,5								
8,0								
8,5								
9,0								
9,5								
10,0								

10 min post mortem

Presens min	pO_2 CSF [mmHg]	mean	n	slope	pAir =baro [hPa]	pCSF [mmHg]	temp. [°C]	pH
0,0	24,45			-33,53				
0,5	7,68	16,06	2	-3,46				
1,0	5,95	12,69	3	1,43				
1,5	6,66			2,42				
2,0	7,88	10,52	5	2,83				
2,5	9,29							
3,0								
3,5								
4,0								
4,5								
5,0					1010		38,5	7,1
5,5								
6,0								
6,5								
7,0								
7,5								
8,0								
8,5								
9,0								
9,5								
10,0								

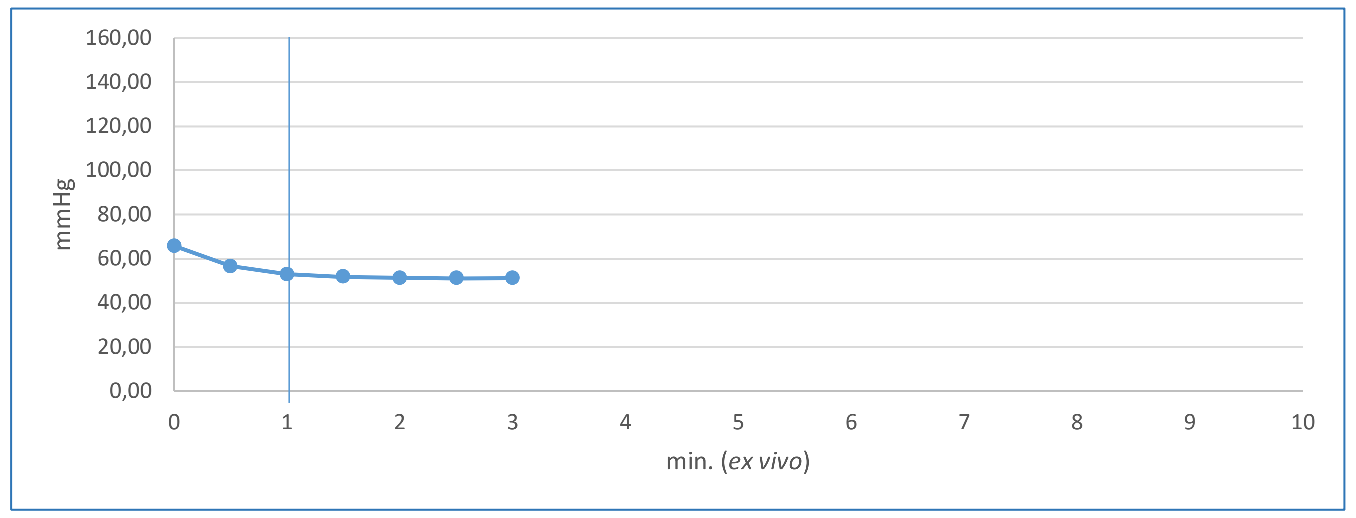

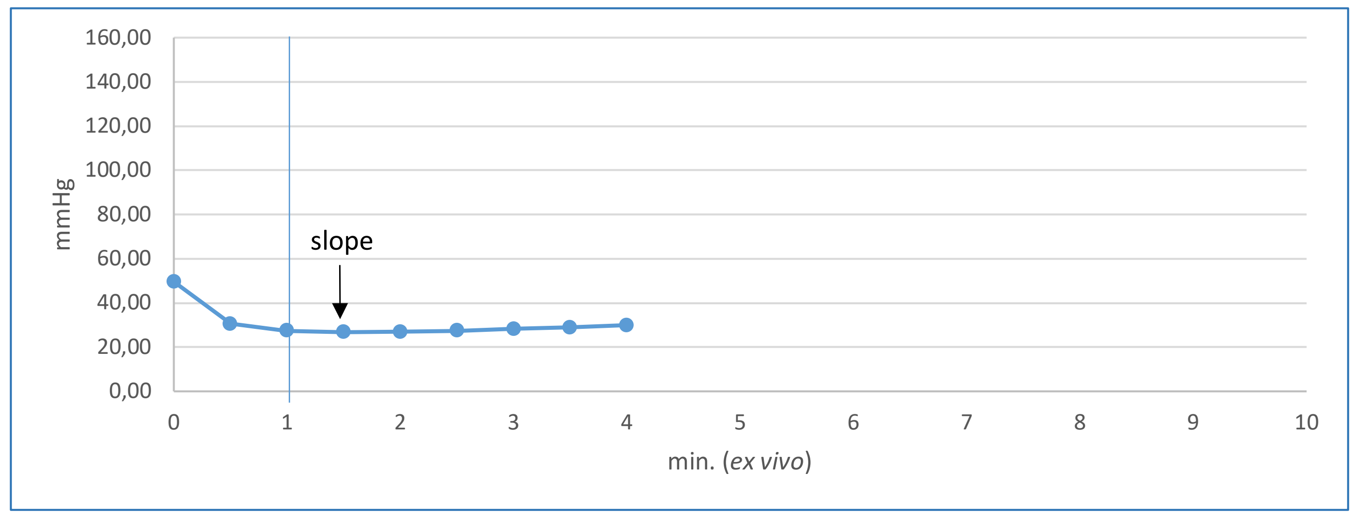

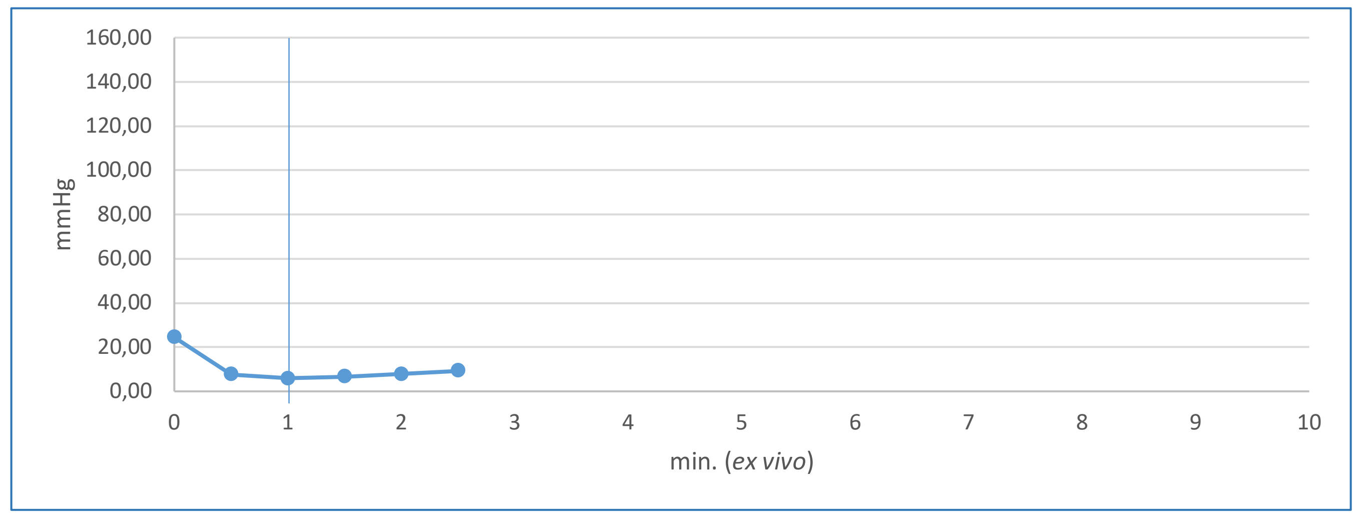

Supplemental table 1: raw data of figure 1d

T8

3 min post mortem

Presens min	pO_2 CSF [mmHg]	mean	n	slope	pAir =baro [hPa]	pCSF [mmHg]	temp. [°C]	pH
0,0	48,14			-13,75				
0,5	41,26	44,70	2	-5,71				
1,0	38,41	42,60	3	-2,65				
1,5	37,08			-1,20				
2,0	36,48	40,27	5	-72,96				
2,5								
3,0								
3,5								
4,0								
4,5								
5,0					1010		38,5	7,3
5,5								
6,0								
6,5								
7,0								
7,5								
8,0								
8,5								
9,0								
9,5								
10,0								

5 min post mortem

Presens min	pO_2 CSF [mmHg]	mean	n	slope	pAir =baro [hPa]	pCSF [mmHg]	temp. [°C]	pH
0,0	68,58			-50,42				
0,5	43,37	55,98	2	-15,31				
1,0	35,72	49,23	3	-7,09				
1,5	32,18			-3,85				
2,0	30,25	42,02	5	-2,17				
2,5	29,16			-1,28				
3,0	28,53			-0,74				
3,5	28,16			-0,44				
4,0	27,94	35,99	9	-55,87				
4,5								
5,0					1010		38,5	7,3
5,5								
6,0								
6,5								
7,0								
7,5								
8,0								
8,5								
9,0								
9,5								
10,0								

10 min post mortem

Presens min	pO_2 CSF [mmHg]	mean	n	slope	pAir =baro [hPa]	pCSF [mmHg]	temp. [°C]	pH
0,0	20,70			-21,50				
0,5	9,95	15,32	2	2,08				
1,0	10,99	13,88	3	4,29				
1,5	13,13							
2,0								
2,5								
3,0								
3,5								
4,0								
4,5								
5,0					1010		38,5	7,0
5,5								
6,0								
6,5								
7,0								
7,5								
8,0								
8,5								
9,0								
9,5								
10,0								

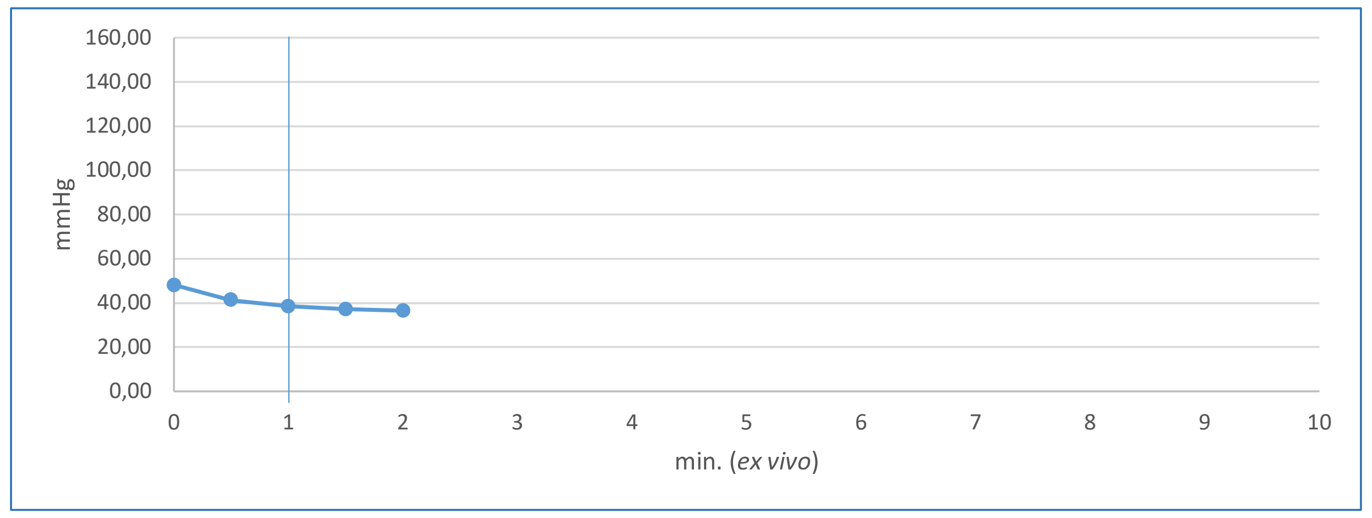

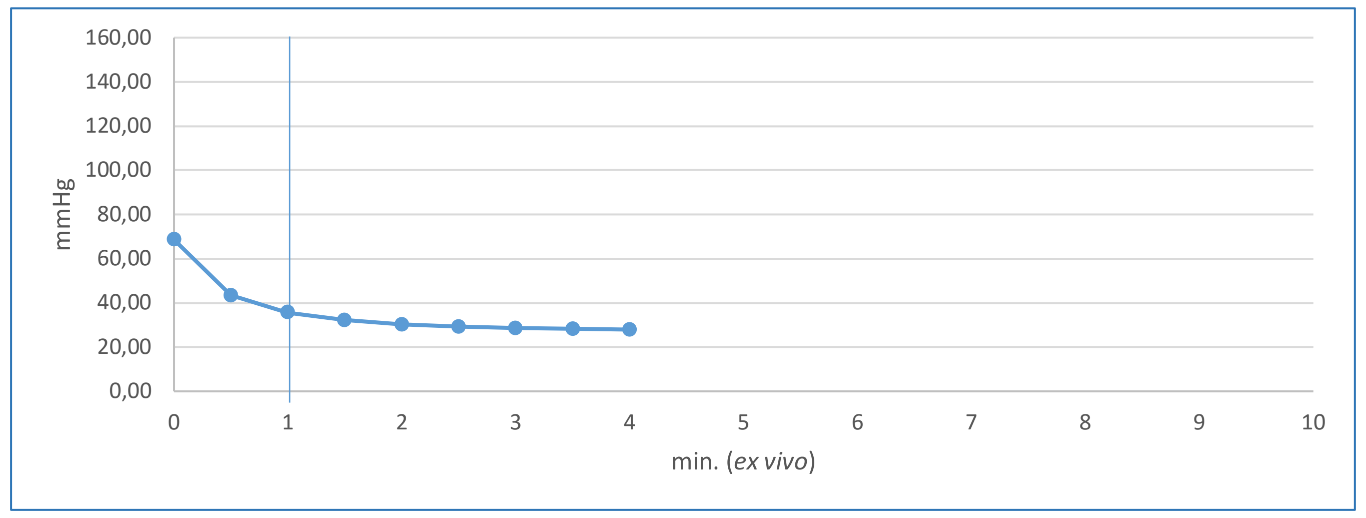

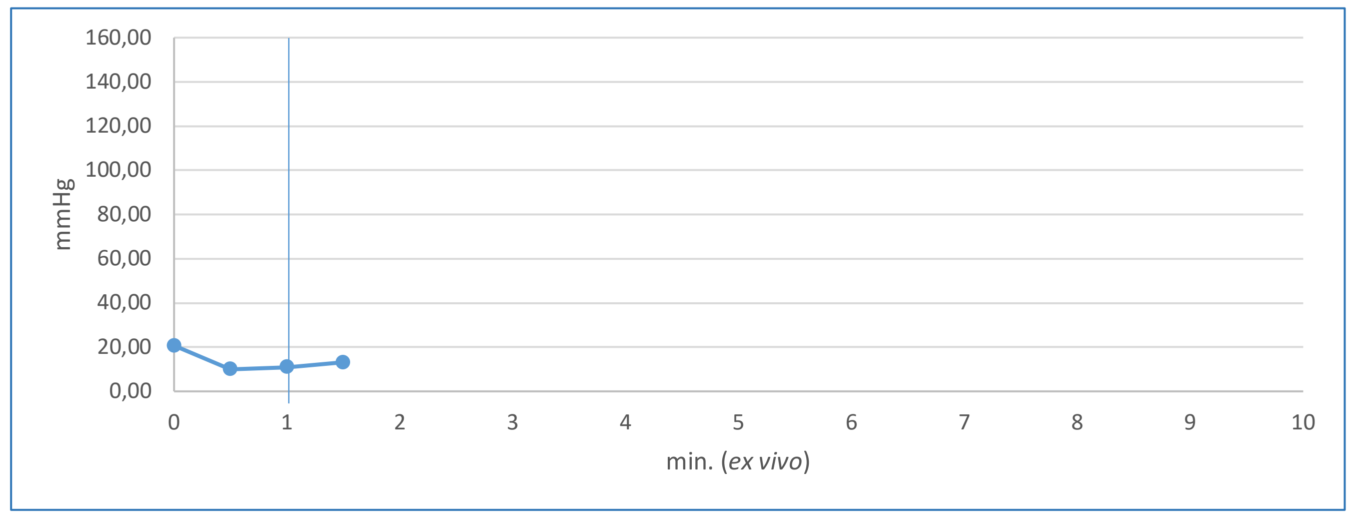

Supplemental table 1: raw data of figure 1d

T13 — 5 min post mortem

Presens min	pO_2 CSF [mmHg]	mean	n	slope	pAir =baro [hPa]	pCSF [mmHg]	temp. [°C]	pH
0,0	79,40			-17,44				
0,5	70,68	75,04	2	-8,83				
1,0	66,27	72,11	3	-5,47				
1,5	63,53			-4,01				
2,0	61,52	68,28	5	-3,25				
2,5	59,90			-2,63				
3,0	58,58			-2,17				
3,5	57,50			-1,82				
4,0	56,59	63,77	9	-1,42				
4,5	55,88			-1,21				
5,0	55,27			-0,92	1008		38,8	7,3
5,5	54,81			-0,64				
6,0	54,49	61,11	13	-0,47				
6,5	54,26			-0,28				
7,0	54,12			-0,13				
7,5	54,05			0,04				
8,0	54,07			0,12				
8,5	54,13			0,21				
9,0	54,24			0,28				
9,5	54,38							
10,0								

10 min post mortem

Presens min	pO_2 CSF [mmHg]	mean	n	slope	pAir =baro [hPa]	pCSF [mmHg]	temp. [°C]	pH
0,0	16,74			-3,02				
0,5	15,24	15,99	2	-0,81				
1,0	14,83	15,60	3	-0,05				
1,5	14,80			0,49				
2,0	15,05	15,33	5	0,83				
2,5	15,46			0,90				
3,0	15,91			0,87				
3,5	16,35			0,80				
4,0	16,74	15,68	9	0,61				
4,5	17,05			0,56				
5,0	17,33				1008		38,8	7,1
5,5								
6,0								
6,5								
7,0								
7,5								
8,0								
8,5								
9,0								
9,5								
10,0								

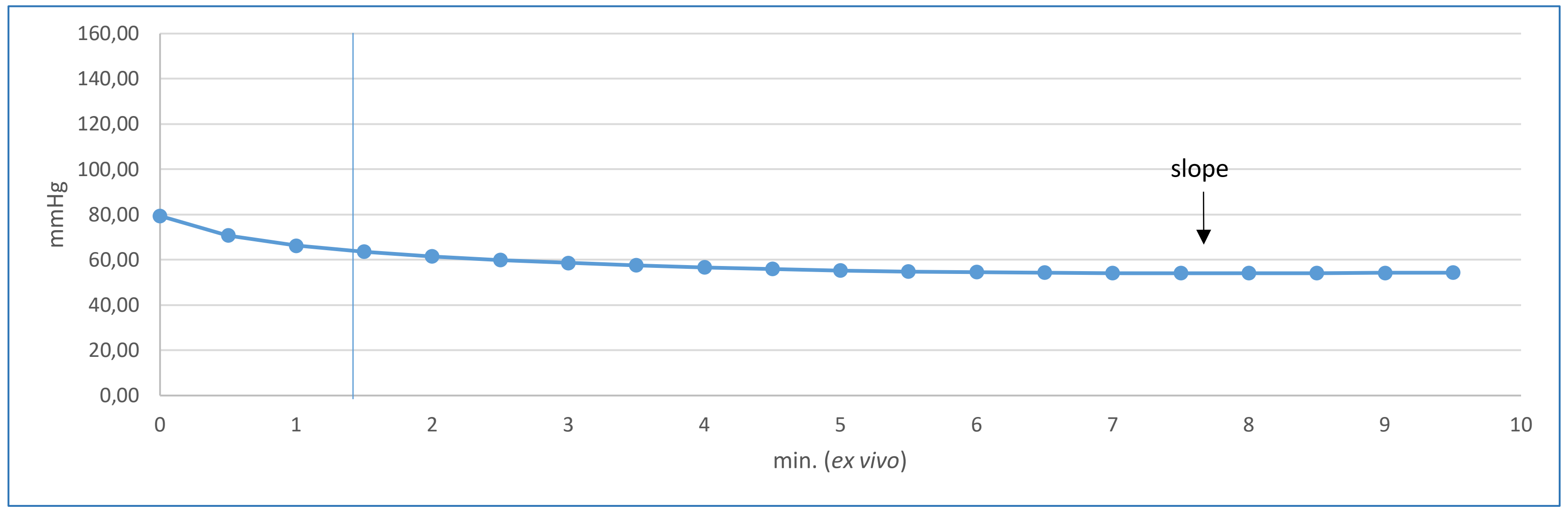

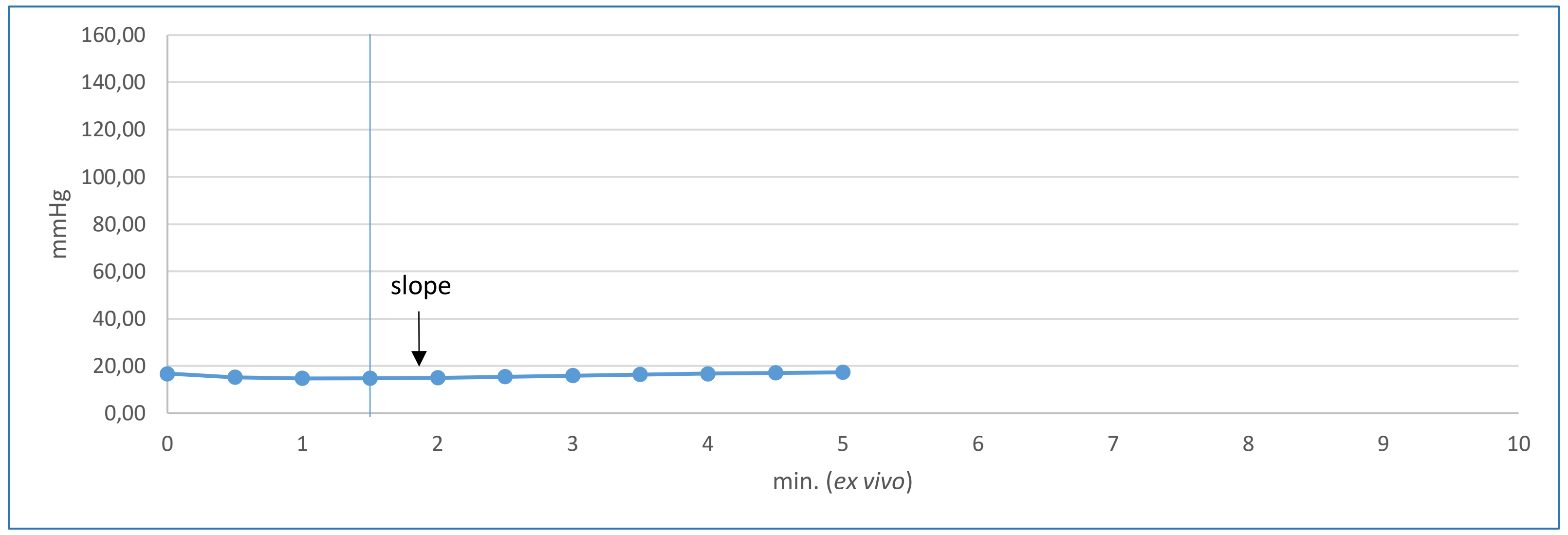

Supplement: Supplementary file 3 — Additional file 3. Table S1. Raw data of Fig. 1d. [file 12868_2021_648_MOESM3_ESM.pdf]
