# Supplementary material for: In vivo oxygen measurement in cerebrospinal fluid of pigs to determine physiologic and pathophysiologic oxygen values during CNS infections
Source: BMC Neurosci. 2021 Jun 28;22:45. doi: 10.1186/s12868-021-00648-x (PMC8240281; doi:10.1186/s12868-021-00648-x)

Supplemental table 2: raw data of figure 3a

V2

-60 min

Presens min	pO_2 CSF [mmHg]	mean	n	slope	pAir =baro [hPa]	pCSF [mmHg]	temp. [°C]	heart rate [bpm]
0,0	59,23			-7,73				
0,5	55,37	57,30	2	-0,26				
1,0	55,24	56,61	3	-0,26				
1,5	55,11			-0,26				
2,0	54,98	55,99	5	-0,26				
2,5	54,85			-0,26	1001	10	40,0	105
3,0	54,72			-0,26				
3,5	54,59							
4,0		55,51	8					
4,5								
5,0								

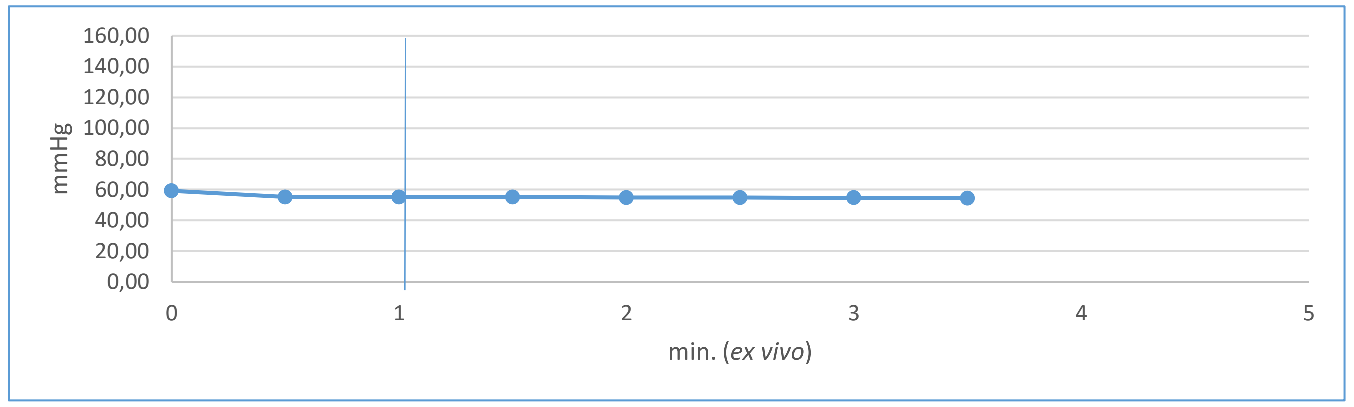

V2

-30 min

Presens min	pO_2 CSF [mmHg]	mean	n	slope	pAir =baro [hPa]	pCSF [mmHg]	temp. [°C]	heart rate [bpm]
0,0	61,23			-10,47				
0,5	56,00	58,62	2	-1,75				
1,0	55,12	57,45	3	-0,05				
1,5	55,10			23,84				
2,0	67,02	58,90	5					
2,5					1001	9	36,5	102
3,0								
3,5								
4,0								
4,5								
5,0								

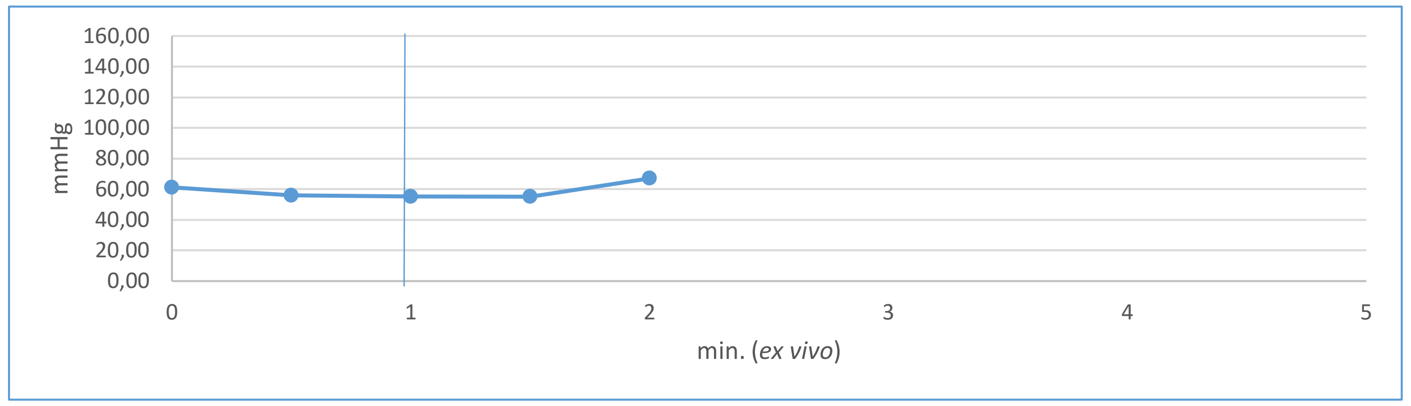

V2

0 min

Presens min	pO_2 CSF [mmHg]	mean	n	slope	pAir =baro [hPa]	pCSF [mmHg]	temp. [°C]	heart rate [bpm]
0,0	56,01			-1,98				
0,5	55,02	55,52	2	0,13				
1,0	55,09	55,37	3	0,00				
1,5	55,09			0,00				
2,0	55,09							
2,5					1001	10	38,1	103
3,0								
3,5								
4,0								
4,5								
5,0								

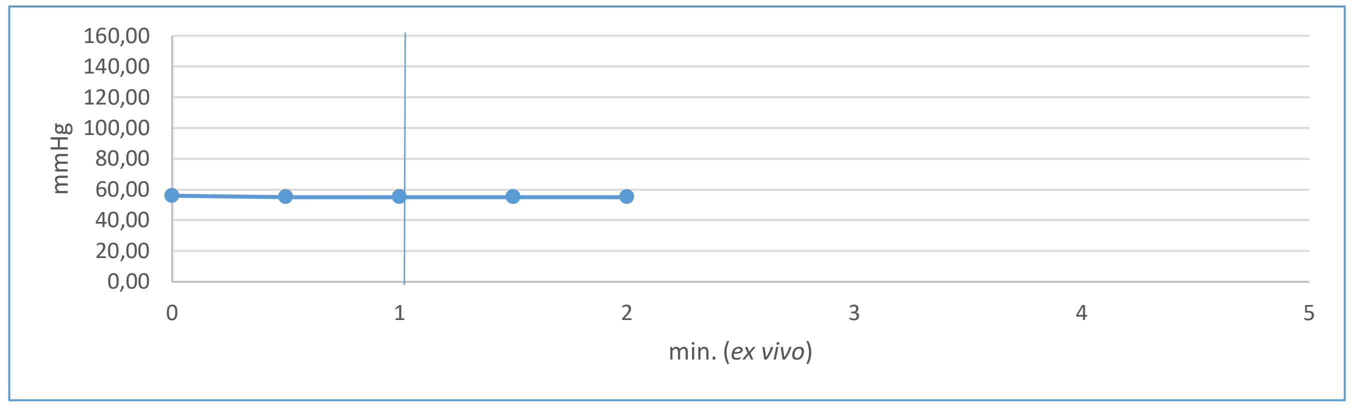

V2

10 min p.m.

Presens min	pO_2 CSF [mmHg]	mean	n	slope	pAir =baro [hPa]	pCSF [mmHg]	temp. [°C]	heart rate [bpm]
0,0	33,60			-8,50				
0,5	29,35	31,48	2	1,68				
1,0	30,19	31,05	3	-6,12				
1,5	27,13			0,91				
2,0	27,59	29,57	5					
2,5	28,27				1001	10	38,1	0
3,0	29,08							
3,5	29,91							
4,0								
4,5								
5,0								

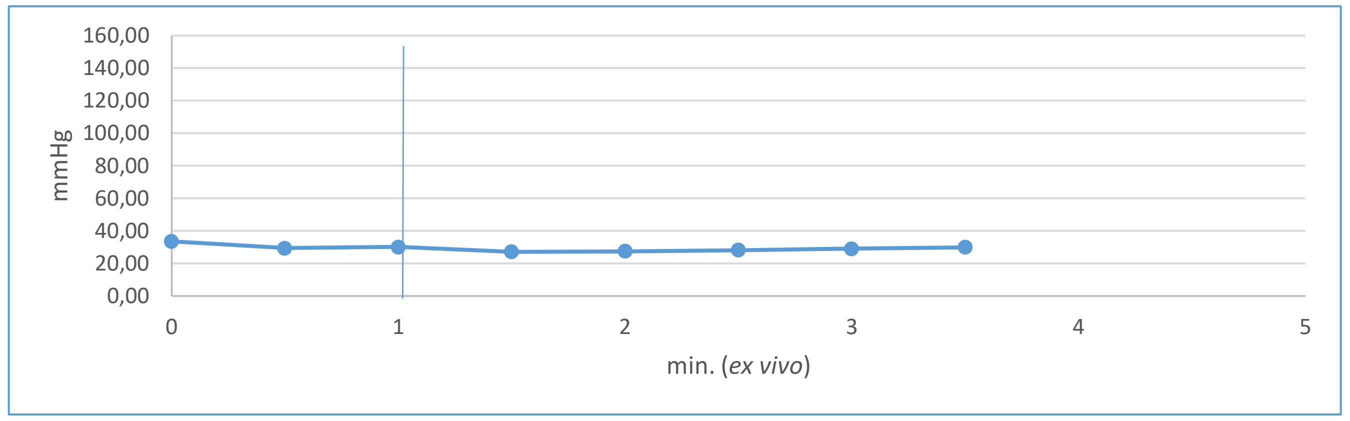

Supplemental table 2: raw data of figure 3b

H2 10 min p.m.

Presens min	pO_2 [mmHg]	mean	n	slope	pAir =baro [hPa]	pCSF [mmHg]	temp. [°C]	pH
0,0	25,51			0,78				
0,5	25,90	25,71	2	0,78				
1,0	26,29	25,90	3	0,78				
1,5	26,68			0,78				
2,0	27,07	26,29	5	0,77				
2,5	27,46			0,78				
3,0	27,85			0,78				
3,5	28,24			0,78				
4,0	28,63	27,07	9	0,78				
4,5	29,02			0,78				
5,0	29,41			0,33	1000	8	39,7	6,2
5,5	29,58							
6,0		27,64	13					
6,5								
7,0								
7,5								
8,0								
8,5								
9,0								
9,5								
10,0								

H4 10 min p.m.

16 h p.i.

Presens min	pO_2 [mmHg]	mean	n	slope	pAir =baro [hPa]	pCSF [mmHg]	temp. [°C]	pH
0,0	12,71			1,04				
0,5	13,22	12,97	2	1,17				
1,0	13,81	13,25	3	0,82				
1,5	14,22			1,76				
2,0	15,10	13,81	5	1,52				
2,5	15,86			1,42				
3,0	16,57			1,34				
3,5	17,24			1,27				
4,0	17,88	15,18	9	1,19				
4,5	18,47							
5,0	19,06				1005	8	38,3	6,8
5,5	19,61							
6,0								
6,5								
7,0								
7,5								
8,0								
8,5								
9,0								
9,5								
10,0								

H5 10 min p.m.

19 h p.i.

Presens min	pO_2 [mmHg]	mean	n	slope	pAir =baro [hPa]	pCSF [mmHg]	temp. [°C]	pH
0,0	24,02			-0,09				
0,5	23,97	24,00	2	0,27				
1,0	24,11	24,03	3	0,41				
1,5	24,31			0,58				
2,0	24,60	24,20	5	0,47				
2,5	24,84			0,47				
3,0	25,07			0,57				
3,5	25,35			0,50				
4,0	25,60	24,65	9	0,51				
4,5	25,86			0,51				
5,0	26,11			0,57	1001	5	37,9	
5,5	26,40			0,69				
6,0	26,74	25,15	13	0,68				
6,5	27,08							
7,0	27,38							
7,5	27,71							
8,0	28,06							
8,5	28,37							
9,0	28,65							
9,5	28,93							
10,0	29,17							

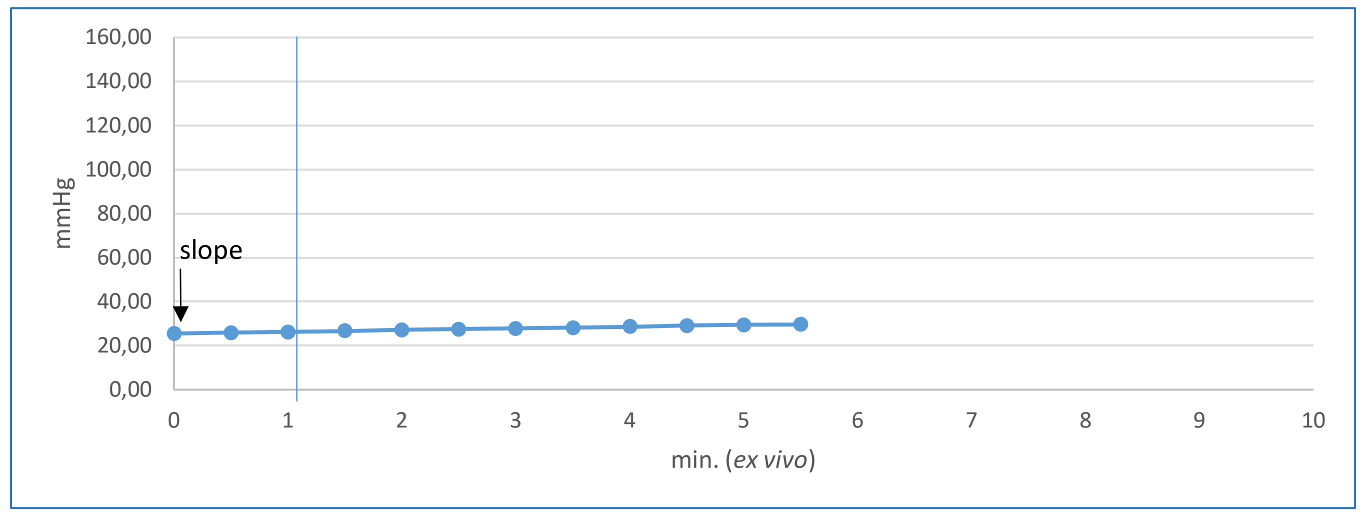

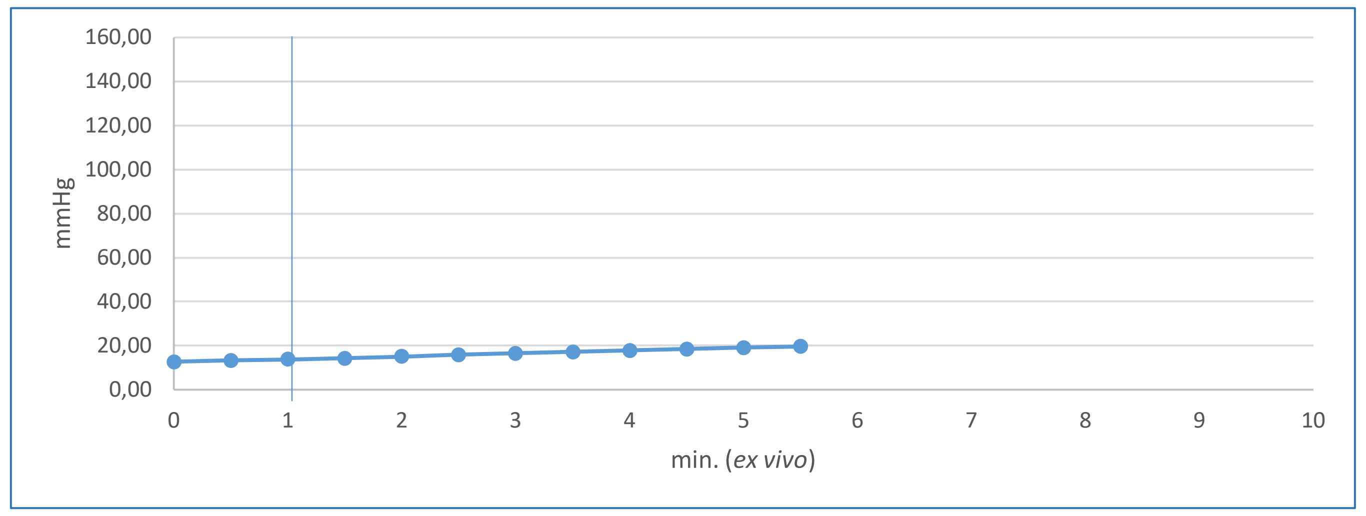

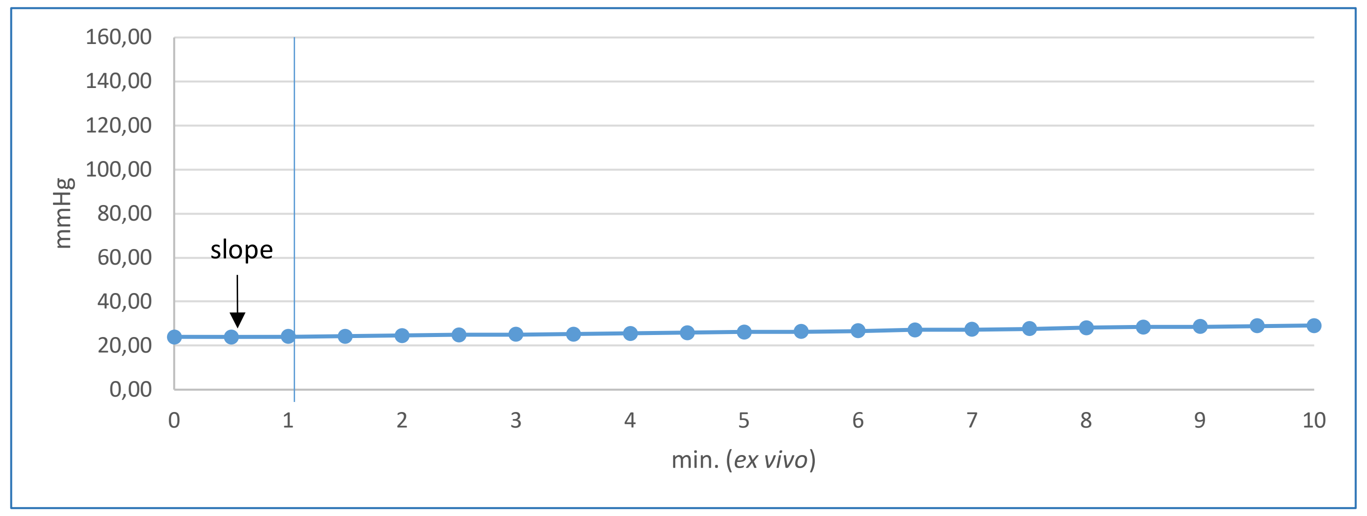

slope

Supplemental table 2: raw data of figure 3b

H7 10 min p.m.

Presens min	pO_2 [mmHg]	mean	n	slope	pAir =baro [hPa]	pCSF [mmHg]	temp. [°C]	pH
0,0	8,90			1,15				
0,5	9,48	9,19	2	2,32				
1,0	10,64	9,67	3	2,68				
1,5	11,98			2,83				
2,0	13,39	10,88	5	3,25				
2,5	15,02			2,42				
3,0	16,22			1,98				
3,5	17,21			1,76				
4,0	18,09	13,44	9	2,60				
4,5	19,39							
5,0					1008	14	40,8	
5,5								
6,0								
6,5								
7,0								
7,5								
8,0								
8,5								
9,0								
9,5								
10,0								

H8 10 min p.m. (16 h p.i.)

Presens min	pO_2 [mmHg]	mean	n	slope	pAir =baro [hPa]	pCSF [mmHg]	temp. [°C]	pH
0,0	13,45			-1,22				
0,5	12,84	13,14	2	0,22				
1,0	12,95	13,08	3	1,11				
1,5	13,50			3,22				
2,0	15,11	13,57	5	2,25				
2,5	16,23							
3,0								
3,5								
4,0								
4,5								
5,0					1012	13	38,7	
5,5								
6,0								
6,5								
7,0								
7,5								
8,0								
8,5								
9,0								
9,5								
10,0								

H12 10 min p.m. (19 h p.i.)

Presens min	pO_2 [mmHg]	mean	n	slope	pAir =baro [hPa]	pCSF [mmHg]	temp. [°C]	pH
0,0	7,06			1,89				
0,5	8,00	7,53	2	1,81				
1,0	8,91	7,99	3					
1,5								
2,0								
2,5								
3,0								
3,5								
4,0								
4,5								
5,0					992	11	38,7	
5,5								
6,0								
6,5								
7,0								
7,5								
8,0								
8,5								
9,0								
9,5								
10,0								

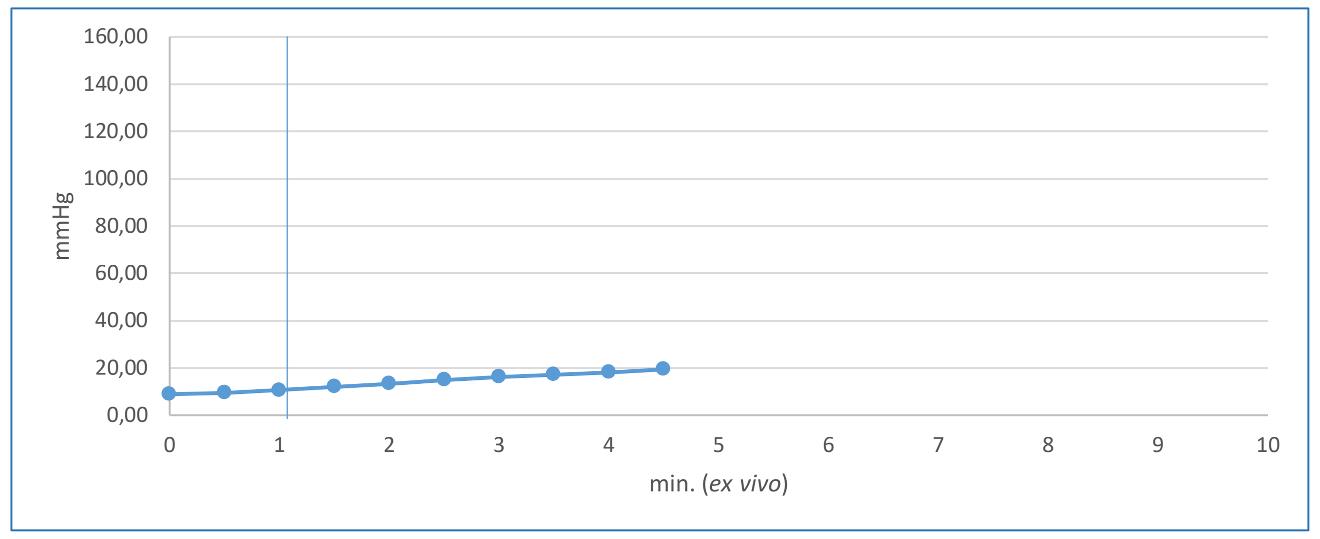

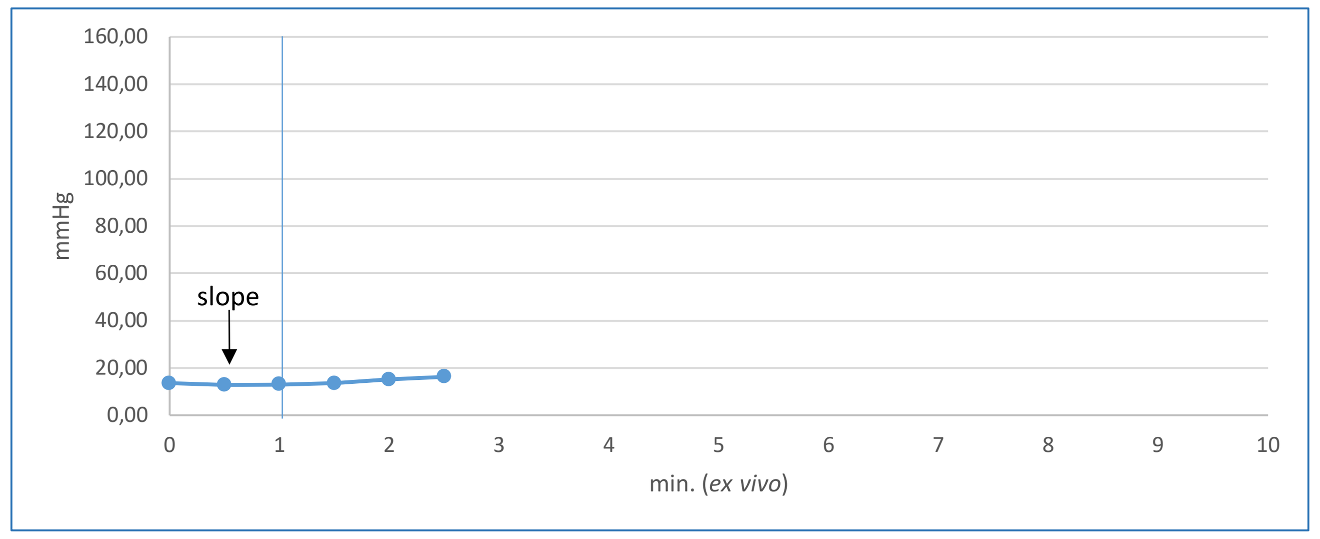

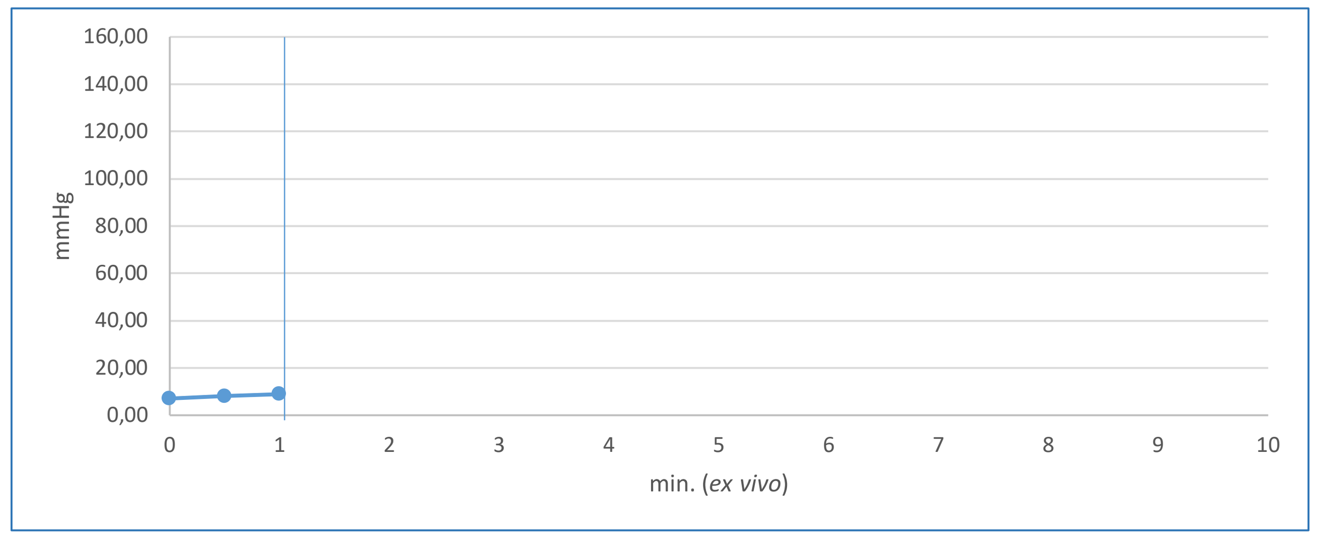

Supplemental table 2: raw data of figure 3c

H3_Hyperoxia_start

Presens min	pO_2 CSF [mmHg]	mean	n	slope	pAir =baro [hPa]	pCSF [mmHg]	temp. [°C]
0,0	62,53			-6,32			
0,5	59,37	60,95	2	-12,92			
1,0	52,91	58,27	3	-3,22			
1,5	51,30			-0,63			
2,0	50,99	55,42	5	-0,89			
2,5	50,54			-0,77			
3,0	50,16			-0,54			
3,5	49,88			-0,51			
4,0	49,63	53,04	9	-0,51			
4,5	49,38			-0,65			
5,0	49,05			-0,30	997	11	39,6
5,5	48,91			-0,31			
6,0	48,75	51,80	13	-0,30			
6,5	48,60			-0,22			
7,0	48,49			-0,29			
7,5	48,34			-0,26			
8,0	48,22			-0,29			
8,5	48,07			-0,29			
9,0	47,92			-0,31			
9,5	47,77						
10,0							

PaO_2 [mmHg] **100,00**

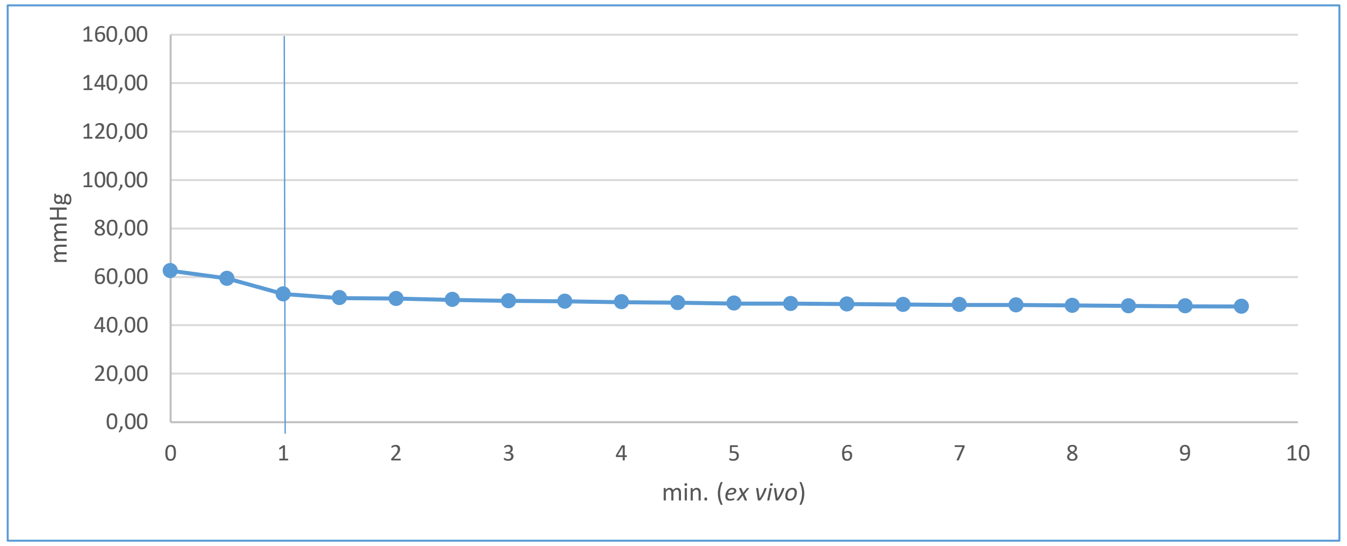

H3_Hyperoxia_after 30 min

Presens min	pO_2 CSF [mmHg]	mean	n	slope	pAir =baro [hPa]	pCSF [mmHg]	temp. [°C]
0,0	63,78			-6,44			
0,5	60,56	62,17	2	-13,18			
1,0	53,97	59,44	3	-3,29			
1,5	52,33			-0,64			
2,0	52,01	56,53	5	-0,91			
2,5	51,55			-0,79			
3,0	51,16			-0,55			
3,5	50,88			-0,52			
4,0	50,62	54,10	9	-0,52			
4,5	50,36			-0,66			
5,0	50,04			-0,30	997	11	39,6
5,5	49,88			-0,11			
6,0	49,83	52,84	13	-0,10			
6,5	49,78			-0,11			
7,0	49,72			-0,10			
7,5	49,67			-0,10			
8,0	49,62			-0,10			
8,5	49,57			-0,04			
9,0	49,55			-0,07			
9,5	49,51			-0,11			
10,0	49,46						

PaO_2 [mmHg] **306,00**

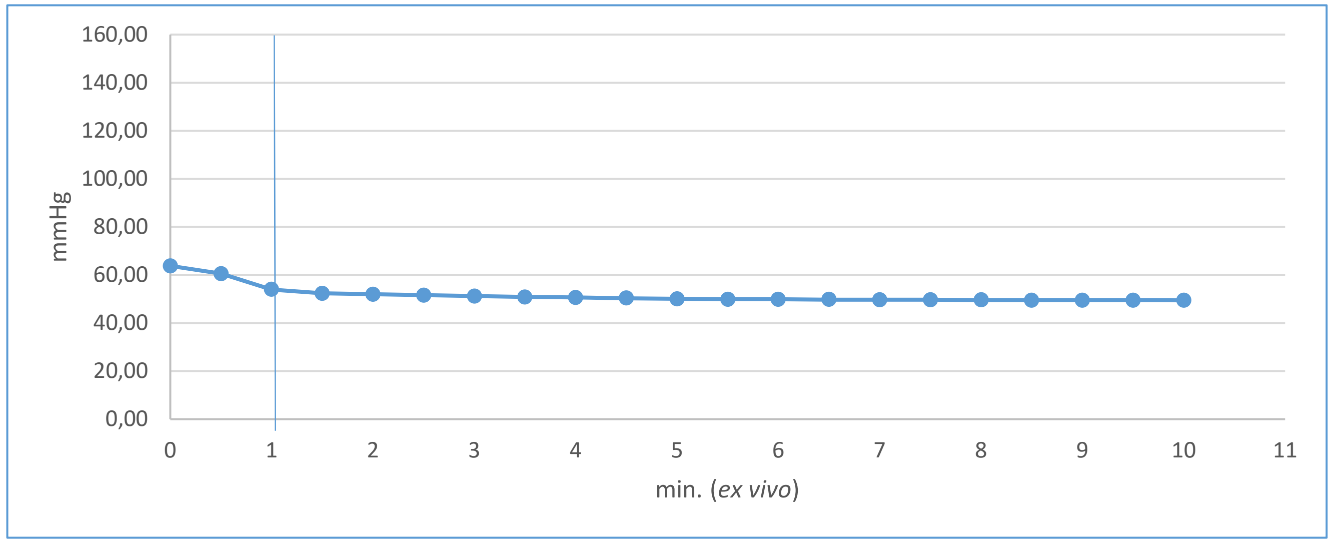

Supplement: Supplementary file 4 — Additional file 4. Table S2. Raw data of Fig. 3a. [file 12868_2021_648_MOESM4_ESM.pdf]
