# Supplementary material for: In vivo oxygen measurement in cerebrospinal fluid of pigs to determine physiologic and pathophysiologic oxygen values during CNS infections
Source: BMC Neurosci. 2021 Jun 28;22:45. doi: 10.1186/s12868-021-00648-x (PMC8240281; doi:10.1186/s12868-021-00648-x)

Supplemental table 3: Raw data figure 4d and 4e

H1

13 h p.i.

Presens	pO$_2$ [mmHg]	mean	n	slope	pAir =baro [hPa]	pCSF [mmHg]	temp. [°C]	pH
min								
0,0	74,16			1,66				6,6
0,5	74,98	74,57	2	1,95				6,6
1,0	75,96	75,03	3	1,12				6,6
1,5	76,52			1,25				6,6
2,0	77,14	75,75	5	1,13				6,6
2,5	77,70			1,24				6,6
3,0	78,33			1,12				6,6
3,5	78,88			0,90				6,6
4,0	79,33	77,00	9	1,09				6,6
4,5	79,88			1,01				6,6
5,0	80,38			0,81	1001	9	37,1	6,6
5,5	80,79			0,84				6,6
6,0	81,21	78,10	13	0,74				6,6
6,5	81,58			0,71				6,6
7,0	81,94			0,70				6,6
7,5	82,28			0,59				6,6
8,0	82,58			0,53				6,6
8,5	82,85			0,56				6,6
9,0	83,13			0,45				6,6
9,5	83,35			0,51				6,6
10,0	83,61							6,6

16 h p.i.

Presens	pO$_2$ [mmHg]	mean	n	slope	pAir =baro [hPa]	pCSF [mmHg]	temp. [°C]	pH
min								
0,0	55,65			0,94				6,5
0,5	56,12	55,89	2	0,85				6,5
1,0	56,55	56,11	3	1,04				6,5
1,5	57,07			0,86				6,5
2,0	57,50	56,58	5	0,73				6,5
2,5	57,86			1,13				6,5
3,0	58,43			0,67				6,5
3,5	58,76			0,90				6,5
4,0	59,21	57,46	9	0,82				6,5
4,5	59,62			0,60				6,5
5,0	59,92			0,92	1001	10	37,9	6,5
5,5	60,38			0,69				6,5
6,0	60,73	58,29	13	0,61				6,5
6,5	61,03			0,58				6,5
7,0	61,32			0,52				6,5
7,5	61,58			0,49				6,5
8,0	61,82			0,49				6,5
8,5	62,07			0,53				6,5
9,0	62,33			0,51				6,5
9,5	62,59			0,55				6,5
10,0	62,87							6,5

19 h p.i.

Presens	pO$_2$ [mmHg]	mean	n	slope	pAir =baro [hPa]	pCSF [mmHg]	temp. [°C]	pH
min								
0,0	53,26			0,97				6,6
0,5	53,74	53,50	2	1,55				6,6
1,0	54,52	53,84	3	1,55				6,6
1,5	55,29			1,36				6,6
2,0	55,97	54,56	5	1,54				6,6
2,5	56,74			1,16				6,6
3,0	57,32			0,86				6,6
3,5	57,75			0,57				6,6
4,0	58,03	55,85	9	0,47				6,6
4,5	58,26			0,22				6,6
5,0	58,38			0,29	999	10	37,5	6,6
5,5	58,52			0,20				6,6
6,0	58,62	56,65	13	0,05				6,6
6,5	58,64			0,05				6,6
7,0	58,67			0,08				6,6
7,5	58,71			0,12				6,6
8,0	58,77			0,17				6,6
8,5	58,85			0,14				6,6
9,0	58,92			0,19				6,6
9,5	59,02			0,09				6,6
10,0	59,06							6,6

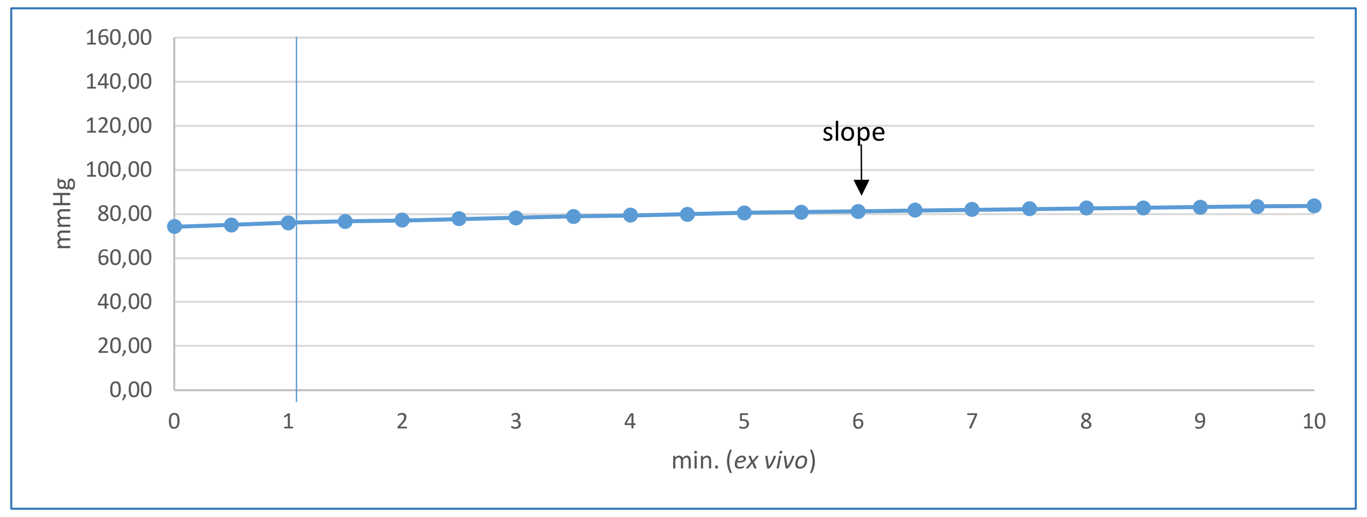

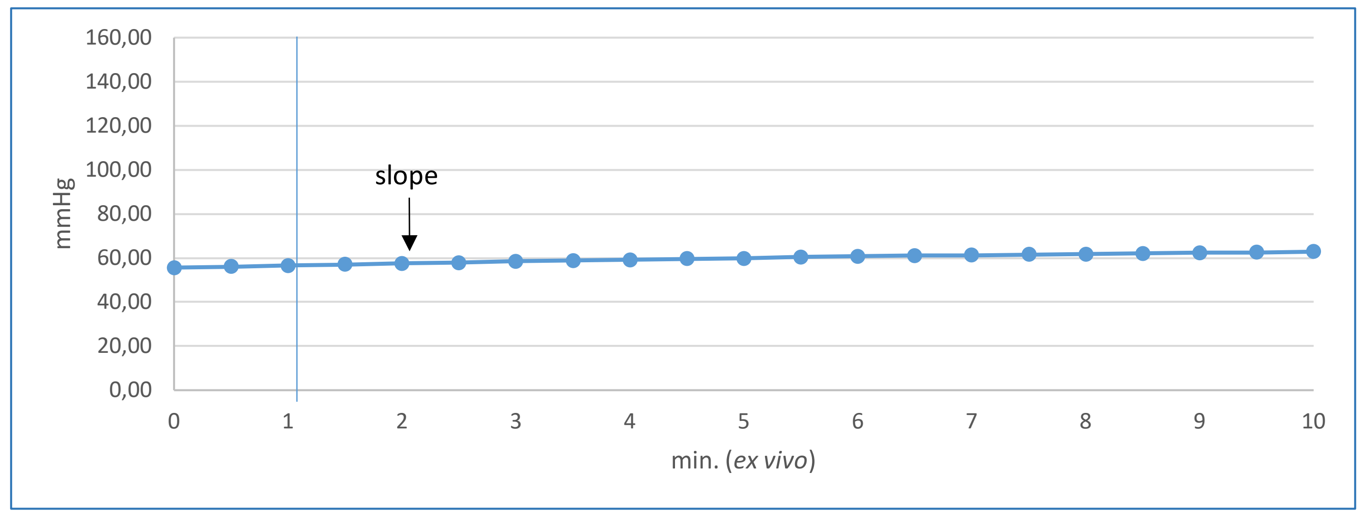

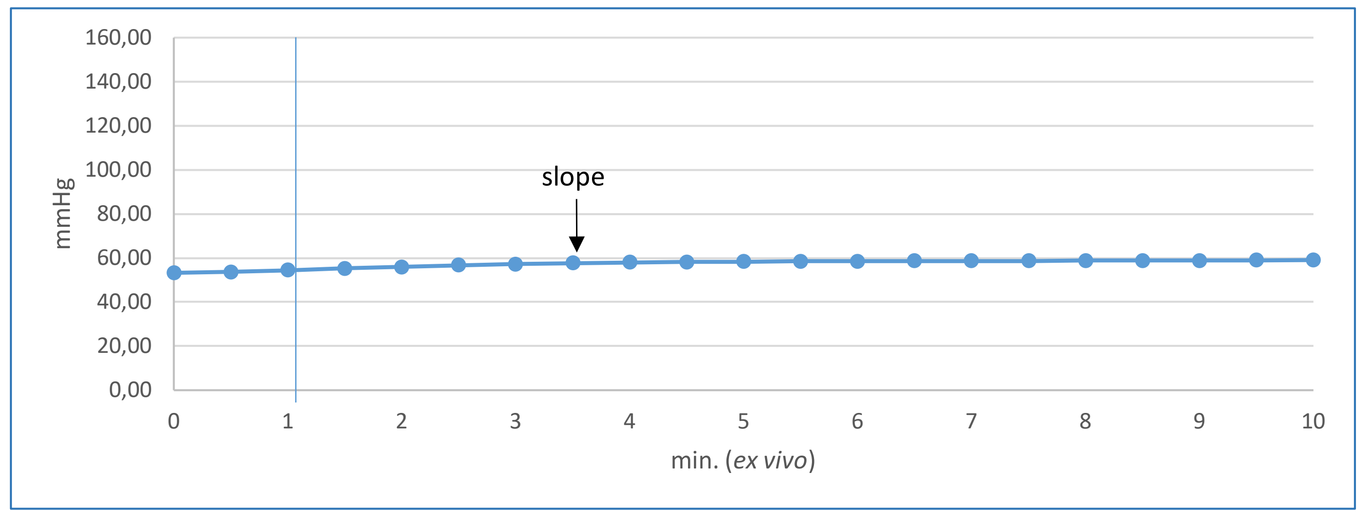

Supplemental table 3: Raw data figure 4d and 4e

H2

13 h p.i.

Presens	pO_2 [mmHg]	mean	n	slope	pAir =baro [hPa]	pCSF [mmHg]	temp. [°C]	pH
min								
0,0								
0,5								
1,0								
1,5								
2,0								
2,5								
3,0								
3,5								
4,0								
4,5	-- failed --							
5,0								
5,5								
6,0								
6,5								
7,0								
7,5								
8,0								
8,5								
9,0								
9,5								
10,0								

16 h p.i.

Presens	pO_2 [mmHg]	mean	n	slope	pAir =baro [hPa]	pCSF [mmHg]	temp. [°C]	pH
min								
0,0	69,31			1,08				6,5
0,5	69,85	69,58	2	2,62				6,5
1,0	71,16	70,11	3	-28,63				6,5
1,5	56,85			0,40				6,5
2,0	57,04	64,84	5	2,58				6,5
2,5	58,33			-0,78				6,5
3,0	57,94			1,95				6,5
3,5	58,92			-0,42				6,5
4,0	58,71	62,01	9	1,21				6,5
4,5	59,31			0,43				6,5
5,0	59,53			0,57	1001	8	39,6	6,5
5,5	59,81			2,16				6,5
6,0	60,89	61,36	13	0,80				6,5
6,5	61,29			0,64				6,5
7,0	61,61			1,02				6,5
7,5	62,12			0,77				6,5
8,0	62,50			0,74				6,5
8,5	62,88			0,75				6,5
9,0	63,25			0,73				6,5
9,5	63,61			0,64				6,5
10,0	63,94							6,5

19 h p.i.

Presens	pO_2 [mmHg]	mean	n	slope	pAir =baro [hPa]	pCSF [mmHg]	temp. [°C]	pH
min								
0,0	53,14			1,20				6,5
0,5	53,74	53,44	2	3,18				6,5
1,0	55,33	54,07	3	3,14				6,5
1,5	56,90			2,17				6,5
2,0	57,99	55,42	5	1,15				6,5
2,5	58,56			0,57				6,5
3,0	58,84			0,48				6,5
3,5	59,08			0,48				6,5
4,0	59,32	56,99	9	0,44				6,5
4,5	59,54			0,39				6,5
5,0	59,73			0,37	1000	8	39,7	6,5
5,5	59,92			0,33				6,5
6,0	60,08	57,86	13	0,33				6,5
6,5	60,25			0,37				6,5
7,0	60,44			0,35				6,5
7,5	60,61			0,43				6,5
8,0	60,83			0,36				6,5
8,5	61,01			0,45				6,5
9,0	61,23			0,48				6,5
9,5	61,48			0,49				6,5
10,0	61,72							6,5

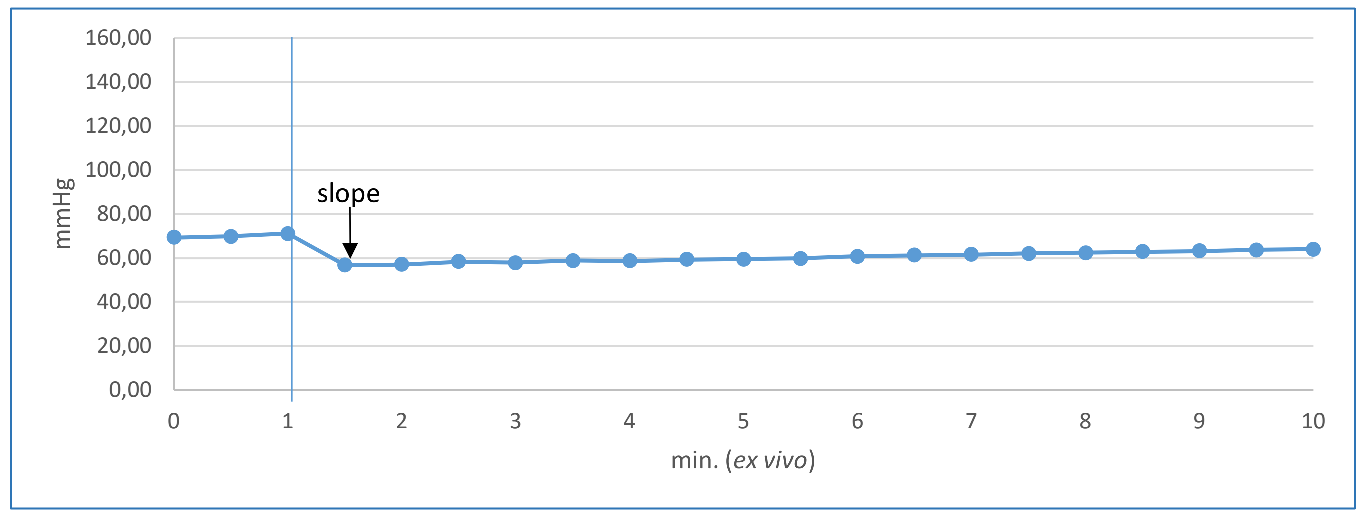

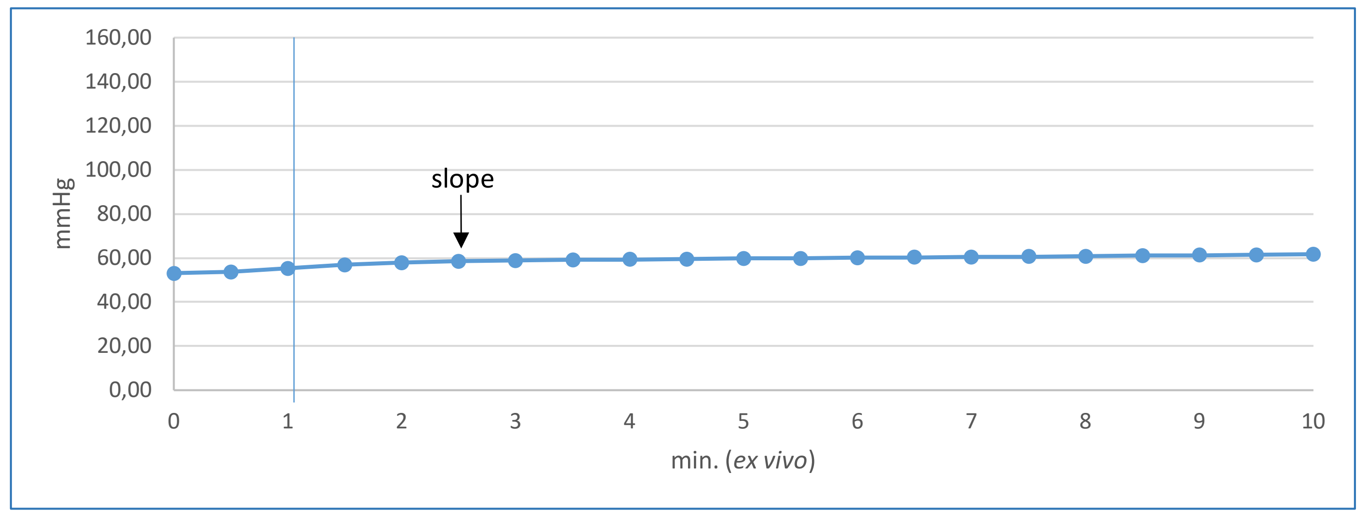

Supplemental table 3: Raw data figure 4d and 4e

H3

13 h p.i.

Presens	pO_2 [mmHg]	mean	n	slope	pAir =baro [hPa]	pCSF [mmHg]	temp. [°C]	pH
min								
0,0								
0,5								
1,0								
1,5								
2,0								
2,5								
3,0								
3,5								
4,0								
4,5	-- failed --							
5,0								
5,5								
6,0								
6,5								
7,0								
7,5								
8,0								
8,5								
9,0								
9,5								
10,0								

16 h p.i.

Presens	pO_2 [mmHg]	mean	n	slope	pAir =baro [hPa]	pCSF [mmHg]	temp. [°C]	pH
min								
0,0	35,79			3,82				6,5
0,5	37,70	36,74	2	3,98				6,5
1,0	39,69	37,73	3	3,38				6,5
1,5	41,38			2,82				6,5
2,0	42,79	39,47	5	2,51				6,5
2,5	44,05			1,90				6,5
3,0	45,00			1,48				6,5
3,5	45,74			1,24				6,5
4,0	46,36	42,05	9	1,04				6,5
4,5	46,87			0,89				6,5
5,0	47,32			0,79	998	11	39,8	6,5
5,5	47,71			0,68				6,5
6,0	48,05	43,73	13	0,71				6,5
6,5	48,41			0,56				6,5
7,0	48,69			0,48				6,5
7,5	48,93			0,60				6,5
8,0	49,23			0,64				6,5
8,5	49,55			0,66				6,5
9,0	49,88			0,59				6,5
9,5	50,18			0,67				6,5
10,0	50,51							6,5

19 h p.i.

Presens	pO_2 [mmHg]	mean	n	slope	pAir =baro [hPa]	pCSF [mmHg]	temp. [°C]	pH
min								
0,0	57,31			-14,89				6,5
0,5	49,87	53,59	2	-2,94				6,5
1,0	48,40	51,86	3	-3,50				6,5
1,5	46,65			-2,60				6,5
2,0	45,35	49,51	5	-1,33				6,5
2,5	44,68			-0,74				6,5
3,0	44,32			-0,49				6,5
3,5	44,07			-0,29				6,5
4,0	43,93	47,17	9	-87,86				6,5
4,5								
5,0					997	11	39,0	
5,5								
6,0								
6,5								
7,0								
7,5								
8,0								
8,5								
9,0								
9,5								
10,0								

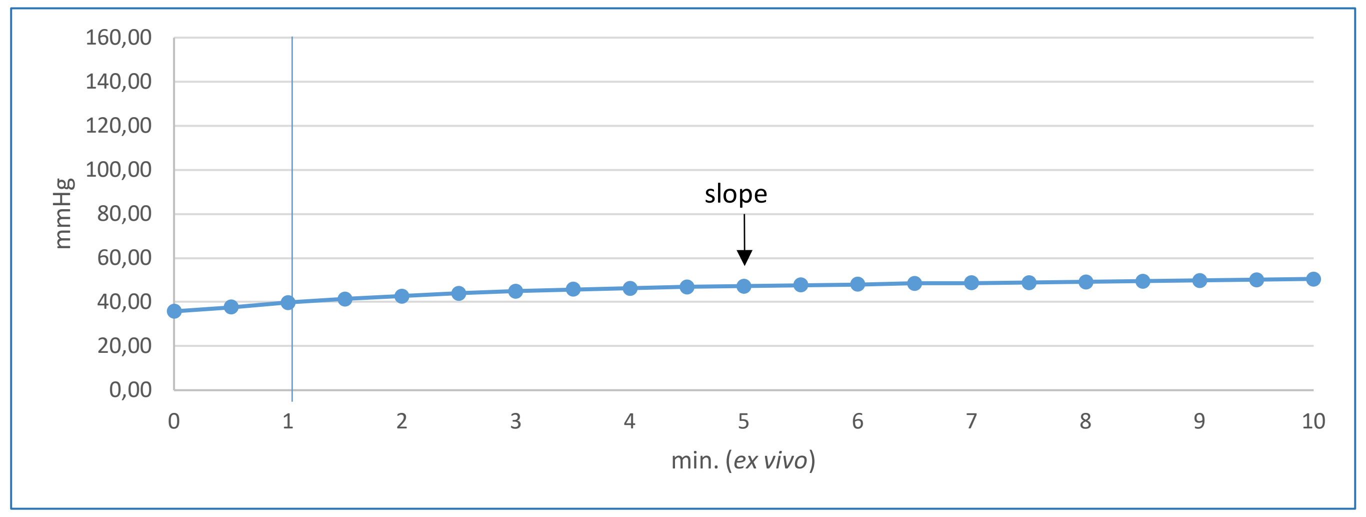

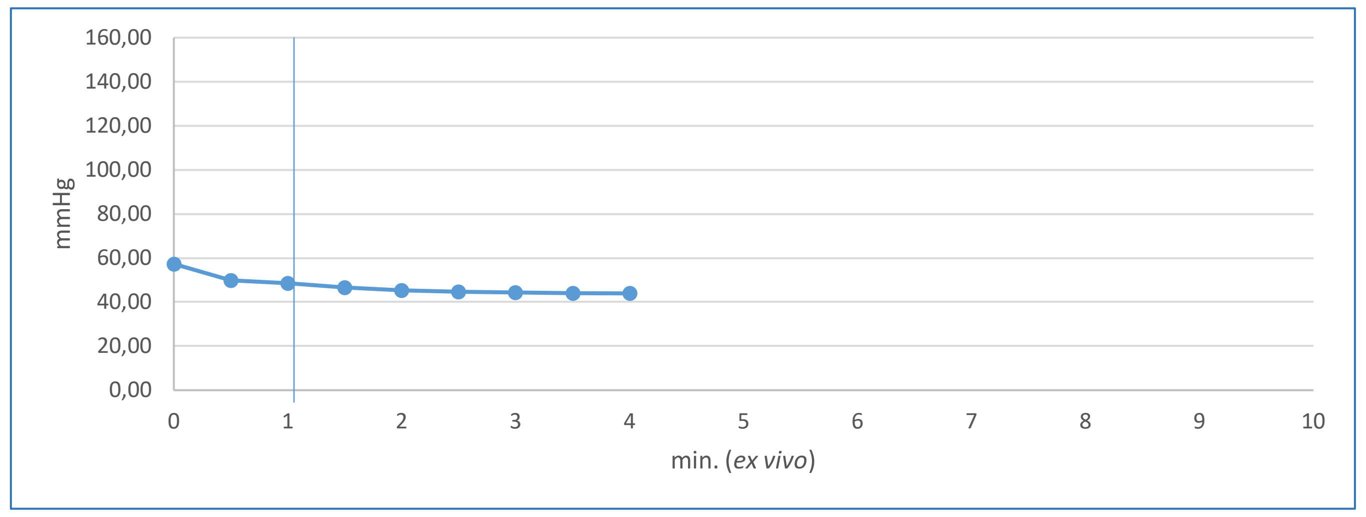

Supplemental table 3: Raw data figure 4d and 4e

H4

13 h p.i.

Presens (min)	pO_2 [mmHg]	mean	n	slope	pAir =baro [hPa]	pCSF [mmHg]	temp. [°C]	pH
0,0	49,70			2,12				7,5
0,5	50,76	50,23	2	1,60				7,5
1,0	51,56	50,67	3	0,54				7,5
1,5	51,83			1,14				7,5
2,0	52,41	51,25	5	1,68				7,5
2,5	53,25			1,60				7,5
3,0	54,05			0,22				7,5
3,5	54,16			2,07				7,5
4,0	55,19	52,55	9	0,96				7,5
4,5	55,67			1,96				7,5
5,0	56,65			0,77	1003	7	37,2	7,5
5,5	57,04			0,72				7,5
6,0	57,40	53,82	13	1,37				7,5
6,5	58,08			1,19				7,5
7,0	58,68			0,73				7,5
7,5	59,04			1,16				7,5
8,0	59,62			1,34				7,5
8,5	60,29			-0,36				7,5
9,0	60,11			2,58				7,5
9,5	61,41			-0,17				7,5
10,0	61,32							7,5

16 h p.i.

Presens (min)	pO_2 [mmHg]	mean	n	slope	pAir =baro [hPa]	pCSF [mmHg]	temp. [°C]	pH
0,0	71,63			-9,05				7,4
0,5	67,10	69,36	2	-4,31				7,4
1,0	64,94	67,89	3	-1,46				7,4
1,5	64,21			-1,61				7,4
2,0	63,41	66,26	5	-0,43				7,4
2,5	63,19			0,29				7,4
3,0	63,34			-0,37				7,4
3,5	63,15			-0,62				7,4
4,0	62,84	64,87	9	0,16				7,4
4,5	62,93			0,34				7,4
5,0	63,10			-0,26	1005	7	38,5	7,4
5,5	62,97			-0,03				7,4
6,0	62,95	64,29	13	-0,33				7,4
6,5	62,79			-1,19				7,4
7,0	62,19			0,45				7,4
7,5	62,42			-0,15				7,4
8,0	62,34			0,40				7,4
8,5	62,54			0,52				7,4
9,0	62,80			-0,18				7,4
9,5	62,71			-0,08				7,4
10,0	62,67							7,4

19 h p.i.

Presens (min)	pO_2 [mmHg]	mean	n	slope	pAir =baro [hPa]	pCSF [mmHg]	temp. [°C]	pH
0,0	61,86			-4,60				7,4
0,5	59,56	60,71	2	-2,85				7,4
1,0	58,13	59,85	3	-1,70				7,4
1,5	57,29			-0,94				7,4
2,0	56,82	58,73	5	-0,55				7,4
2,5	56,54			-0,18				7,4
3,0	56,45			-0,04				7,4
3,5	56,43			0,06				7,4
4,0	56,46	57,73	9	0,20				7,4
4,5	56,56			0,19				7,4
5,0	56,65			0,26	1005	8	38,3	7,4
5,5	56,78			0,24				7,4
6,0	56,91	57,42	13	0,29				7,4
6,5	57,05			0,28				7,4
7,0	57,19			0,34				7,4
7,5	57,36			0,27				7,4
8,0	57,50			0,36				7,4
8,5	57,68			0,28				7,4
9,0	57,82			0,24				7,4
9,5	57,94			0,26				7,4
10,0	58,07							7,4

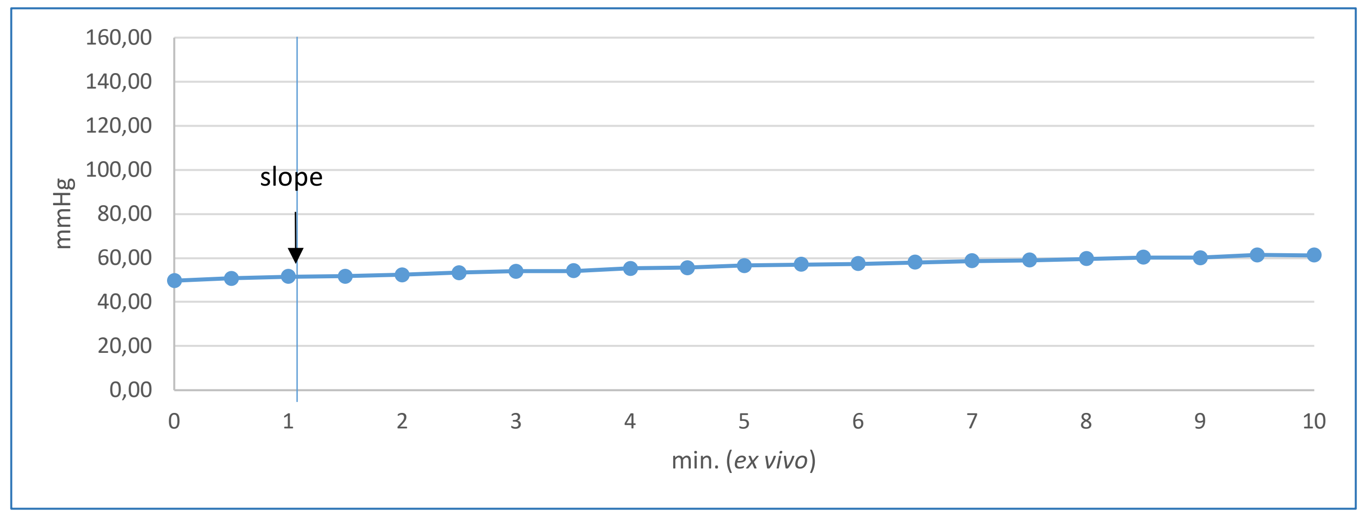

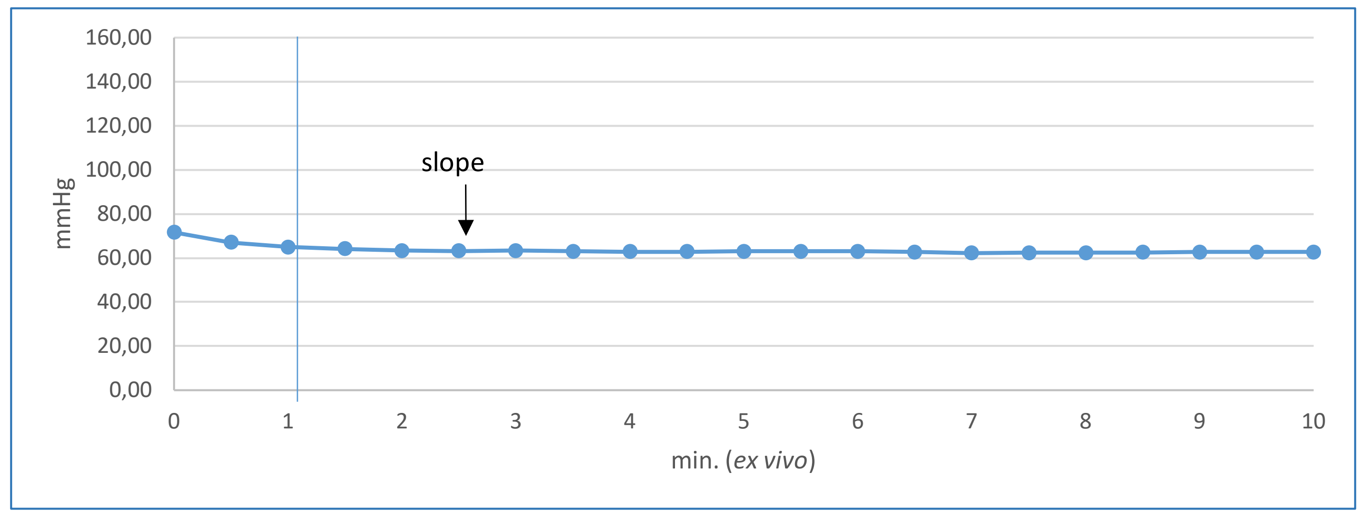

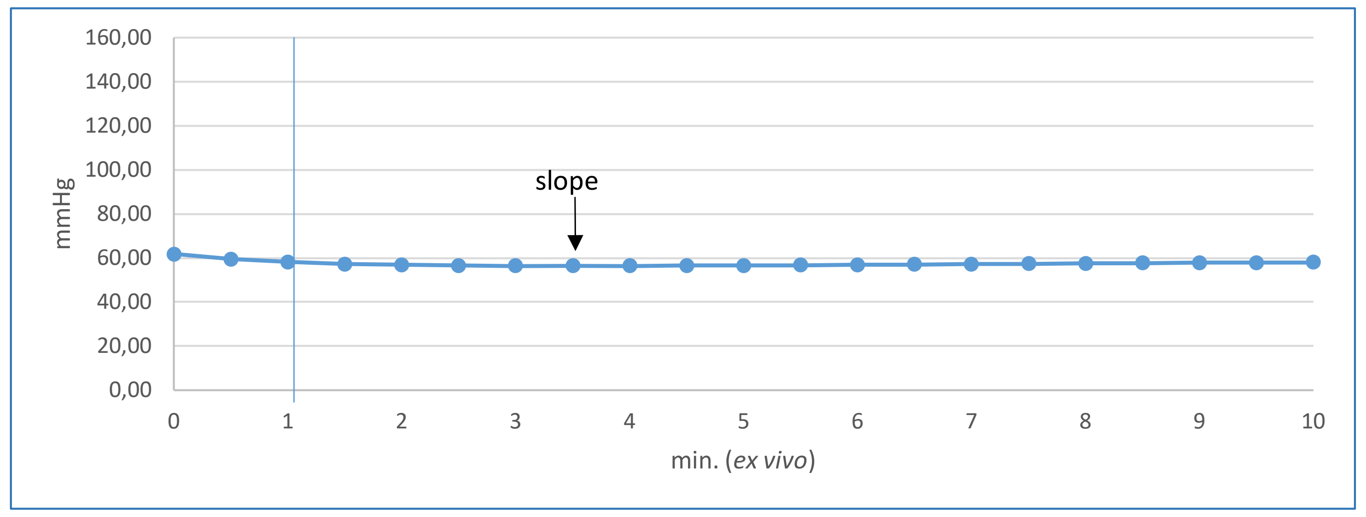

Supplemental table 3: Raw data figure 4d and 4e

H5

13 h p.i.

Presens min	pO_2 [mmHg]	mean	n	slope	pAir =baro [hPa]	pCSF [mmHg]	temp. [°C]	pH
0,0	-- failed --							
0,5								
1,0								
1,5								
2,0								
2,5								
3,0								
3,5								
4,0								
4,5								
5,0								
5,5								
6,0								
6,5								
7,0								
7,5								
8,0								
8,5								
9,0								
9,5								
10,0								

16 h p.i.

Presens min	pO_2 [mmHg]	mean	n	slope	pAir =baro [hPa]	pCSF [mmHg]	temp. [°C]	pH
0,0	53,72			0,05				7,5
0,5	53,75	53,73	2	0,90				7,5
1,0	54,20	53,89	3	1,72				7,5
1,5	55,06			1,95				7,5
2,0	56,04	54,55	5	1,94				7,5
2,5	57,01			2,05				7,5
3,0	58,03			1,86				7,5
3,5	58,96			3,12				7,5
4,0	60,52	56,36	9	0,15				7,5
4,5	60,59			1,31				7,5
5,0	61,25			0,97	1005	4	38,5	7,5
5,5	61,73			0,82				7,5
6,0	62,14	57,92	13	1,42				7,5
6,5	62,85							7,5
7,0								
7,5								
8,0								
8,5								
9,0								
9,5								
10,0								

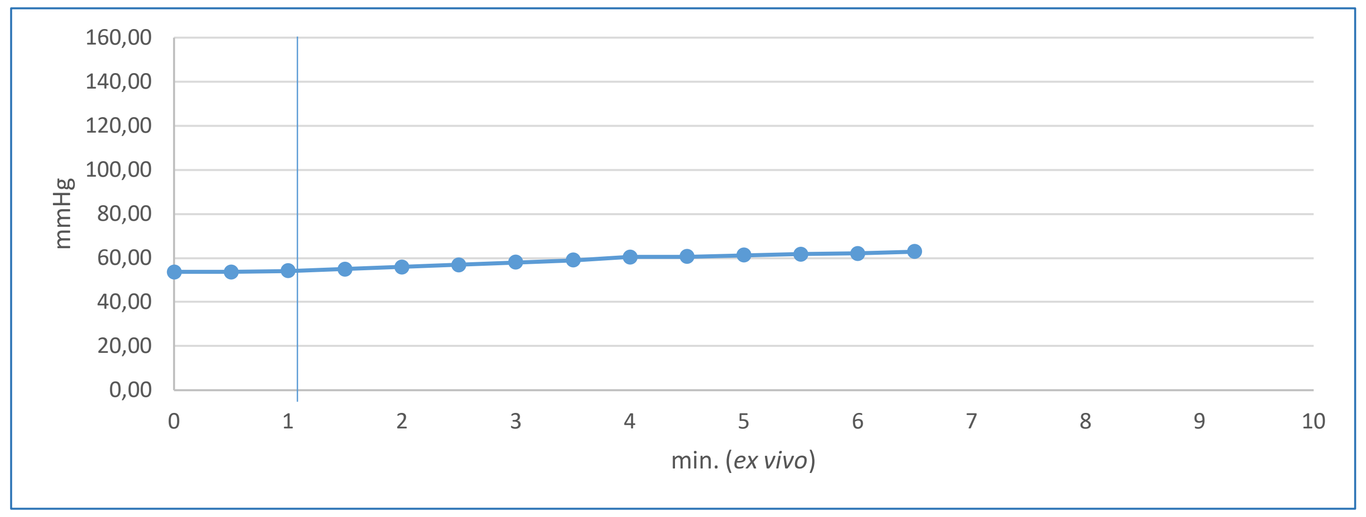

19 h p.i.

Presens min	pO_2 [mmHg]	mean	n	slope	pAir =baro [hPa]	pCSF [mmHg]	temp. [°C]	pH
0,0	61,02			-0,60				7,5
0,5	60,72	60,87	2	-0,21				7,5
1,0	60,62	60,79	3	0,74				7,5
1,5	60,99			1,22				7,5
2,0	61,60	60,99	5	1,01				7,5
2,5	62,10			0,78				7,5
3,0	62,49			0,65				7,5
3,5	62,82			0,54				7,5
4,0	63,08	61,72	9	0,49				7,5
4,5	63,33			0,44				7,5
5,0	63,55			0,46	1001	5	37,9	7,5
5,5	63,78			0,29				7,5
6,0	63,93	62,31	13	0,37				7,5
6,5	64,11			0,40				7,5
7,0	64,31			0,38				7,5
7,5	64,50			0,44				7,5
8,0	64,72							7,5
8,5								
9,0								
9,5								
10,0								

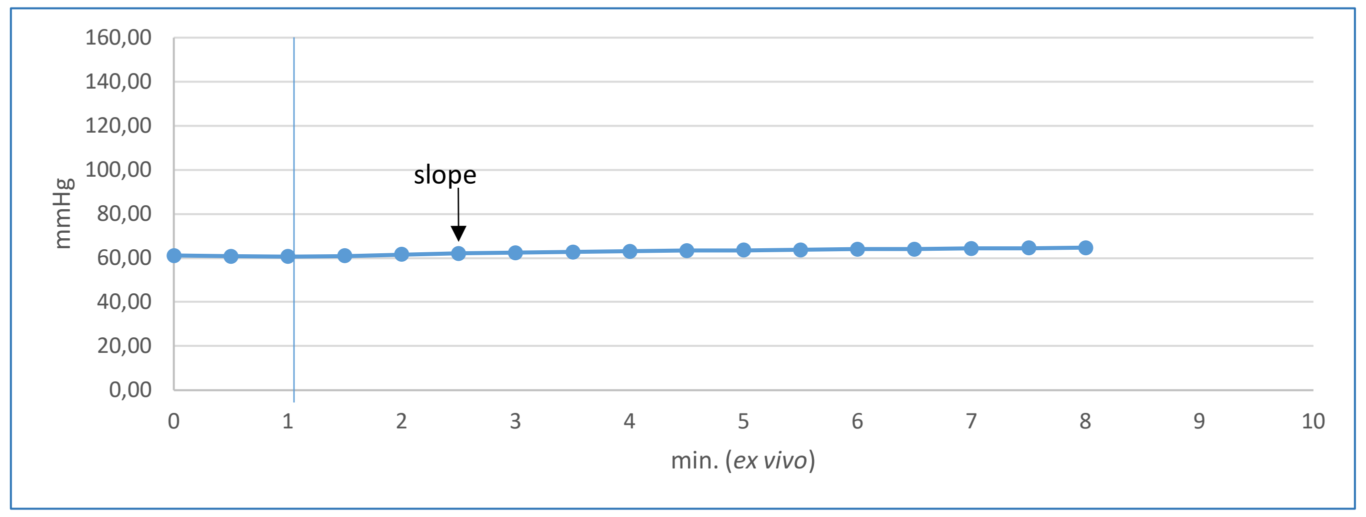

Supplemental table 3: Raw data figure 4d and 4e

H6

13 h p.i.

Presens	pO_2 [mmHg]	mean	n	slope	pAir =baro [hPa]	pCSF [mmHg]	temp. [°C]	pH
min								
0,0	52,65			-3,96				7,5
0,5	50,67	51,66	2	-1,28				7,5
1,0	50,03	51,11	3	-0,07				7,5
1,5	49,99			0,54				7,5
2,0	50,26	50,72	5	0,89				7,5
2,5	50,71			0,98				7,5
3,0	51,20			0,95				7,5
3,5	51,67			0,77				7,5
4,0	52,05	51,03	9	0,87				7,5
4,5	52,49			0,65				7,5
5,0	52,81			0,61	992	4	37,7	7,5
5,5	53,12			0,58				7,5
6,0	53,40	51,62	13	0,44				7,5
6,5	53,63							7,5
7,0								
7,5								
8,0								
8,5								
9,0								
9,5								
10,0								

16 h p.i.

Presens	pO_2 [mmHg]	mean	n	slope	pAir =baro [hPa]	pCSF [mmHg]	temp. [°C]	pH
min								
0,0	72,59			-2,53				7,4
0,5	71,33	71,96	2	-0,74				7,4
1,0	70,95	71,62	3	-0,07				7,4
1,5	70,92			0,02				7,4
2,0	70,93	71,34	5	0,14				7,4
2,5	71,00			0,53				7,4
3,0	71,26			0,51				7,4
3,5	71,52			0,61				7,4
4,0	71,83	71,37	9	0,77				7,4
4,5	72,21			0,81				7,4
5,0	72,61			0,93	986	10	38,9	7,4
5,5	73,07							7,4
6,0								
6,5								
7,0								
7,5								
8,0								
8,5								
9,0								
9,5								
10,0								

19 h p.i.

Presens	pO_2 [mmHg]	mean	n	slope	pAir =baro [hPa]	pCSF [mmHg]	temp. [°C]	pH
min								
0,0	57,31			-5,91				7,4
0,5	54,35	55,83	2	-2,69				7,4
1,0	53,00	54,89	3	-1,08				7,4
1,5	52,46			-0,01				7,4
2,0	52,46	53,92	5	0,29				7,4
2,5	52,61			0,62				7,4
3,0	52,92			0,67				7,4
3,5	53,25			0,74				7,4
4,0	53,62	53,55	9	0,56				7,4
4,5	53,90			0,78				7,4
5,0	54,29			0,77	982	8	39,9	7,4
5,5	54,68			0,83				7,4
6,0	55,09	53,84	13	0,74				7,4
6,5	55,46			0,85				7,4
7,0	55,89			0,91				7,4
7,5	56,34			0,98				7,4
8,0	56,83			0,89				7,4
8,5	57,28			1,01				7,4
9,0	57,78			0,96				7,4
9,5	58,27			0,83				7,4
10,0	58,68							7,4

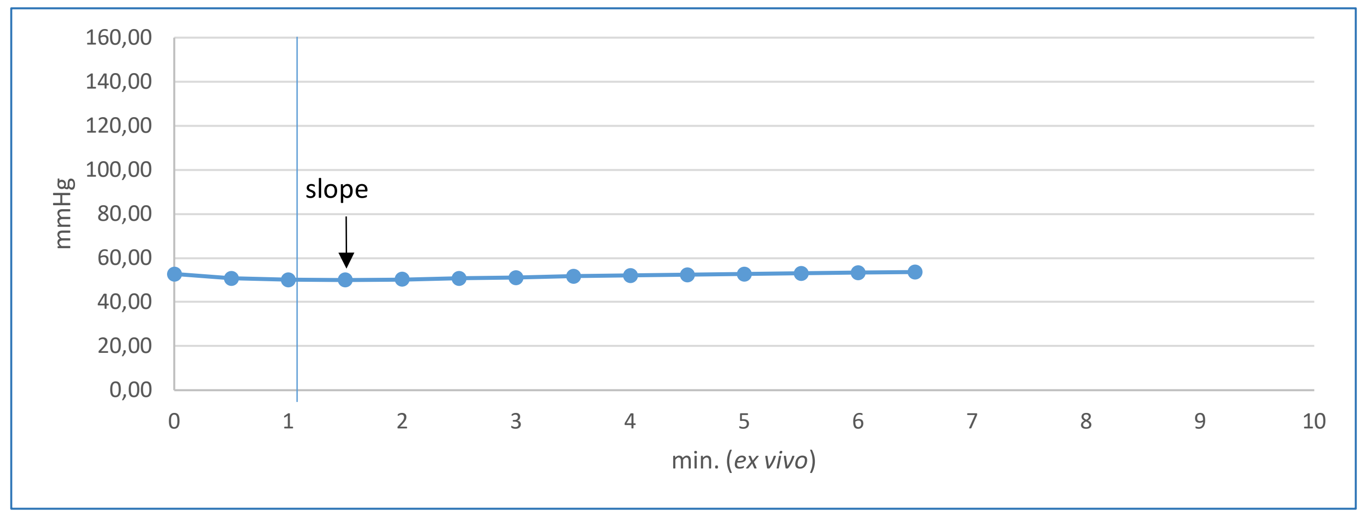

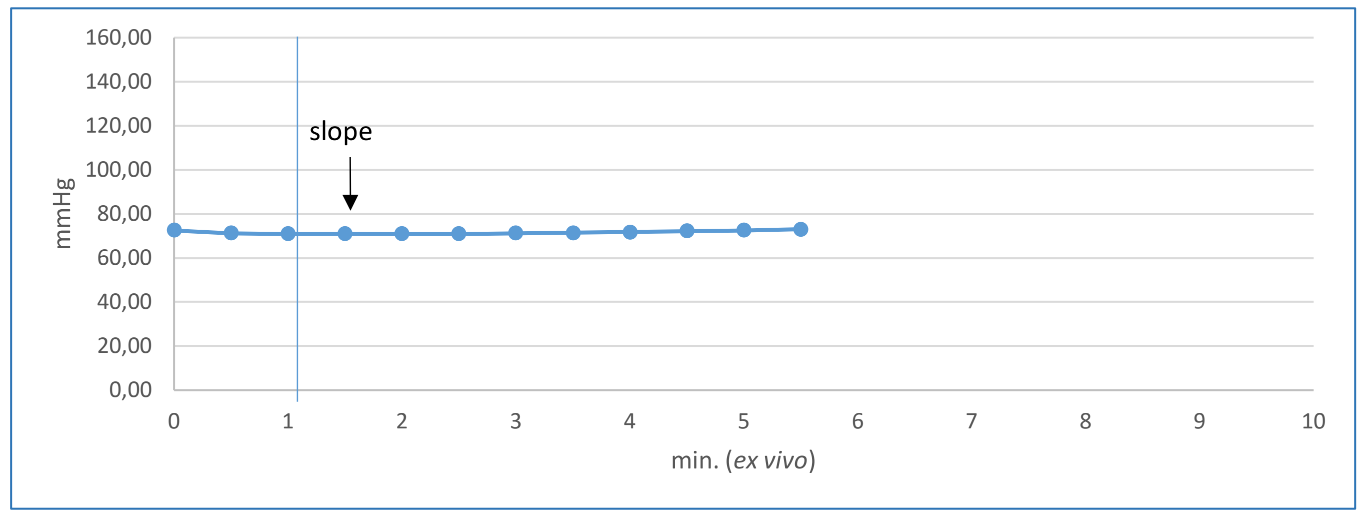

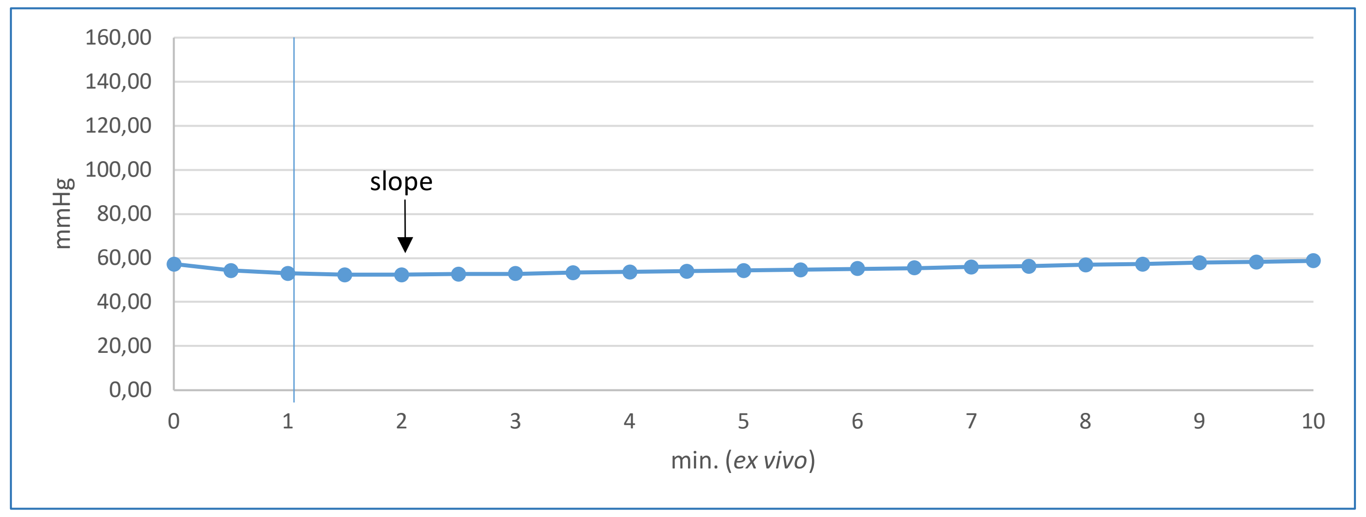

Supplemental table 3: Raw data figure 4d and 4e

H7

13 h p.i.

Presens min	pO_2 [mmHg]	mean	n	slope	pAir =baro [hPa]	pCSF [mmHg]	temp. [°C]	pH
0,0	55,76			0,80				7,3
0,5	56,16	55,96	2	1,56				7,3
1,0	56,94	56,28	3	1,67				7,3
1,5	57,77			3,02				7,3
2,0	59,28	57,18	5	0,19				7,3
2,5	59,38			1,30				7,3
3,0	60,03			1,28				7,3
3,5	60,67			0,95				7,3
4,0	61,14	58,57	9	0,98				7,3
4,5	61,63			-0,14				7,3
5,0	61,56			1,81	1004	4	39,4	7,3
5,5	62,47			0,86				7,3
6,0	62,90	59,67	13	0,86				7,3
6,5	63,33			0,80				7,3
7,0	63,73			0,79				7,3
7,5	64,12			0,77				7,3
8,0	64,51			0,73				7,3
8,5	64,87			0,83				7,3
9,0	65,29			0,65				7,3
9,5	65,61			0,71				7,3
10,0	65,97							7,3

16 h p.i.

Presens min	pO_2 [mmHg]	mean	n	slope	pAir =baro [hPa]	pCSF [mmHg]	temp. [°C]	pH
0,0	60,82			-4,56				7,3
0,5	58,54	59,68	2	-0,66				7,3
1,0	58,21	59,19	3	0,18				7,3
1,5	58,30			0,42				7,3
2,0	58,51	58,88	5	0,53				7,3
2,5	58,78			0,69				7,3
3,0	59,12			0,75				7,3
3,5	59,50			0,81				7,3
4,0	59,90	59,07	9	0,77				7,3
4,5	60,29							7,3
5,0					1006	9	40,7	
5,5								
6,0								
6,5								
7,0								
7,5								
8,0								
8,5								
9,0								
9,5								
10,0								

19 h p.i.

Presens min	pO_2 [mmHg]	mean	n	slope	pAir =baro [hPa]	pCSF [mmHg]	temp. [°C]	pH
0,0	55,21			-9,00				7,4
0,5	50,70	52,95	2	-2,61				7,4
1,0	49,40	51,77	3	0,25				7,4
1,5	49,53			1,13				7,4
2,0	50,09	50,98	5	1,37				7,4
2,5	50,77			1,26				7,4
3,0	51,40			1,09				7,4
3,5	51,95			1,00				7,4
4,0	52,45	51,28	9	0,85				7,4
4,5	52,87			0,82				7,4
5,0	53,28			0,82	1008	14	40,8	7,4
5,5	53,69			0,86				7,4
6,0	54,12	51,96	13	0,85				7,4
6,5	54,55							7,4
7,0								
7,5								
8,0								
8,5								
9,0								
9,5								
10,0								

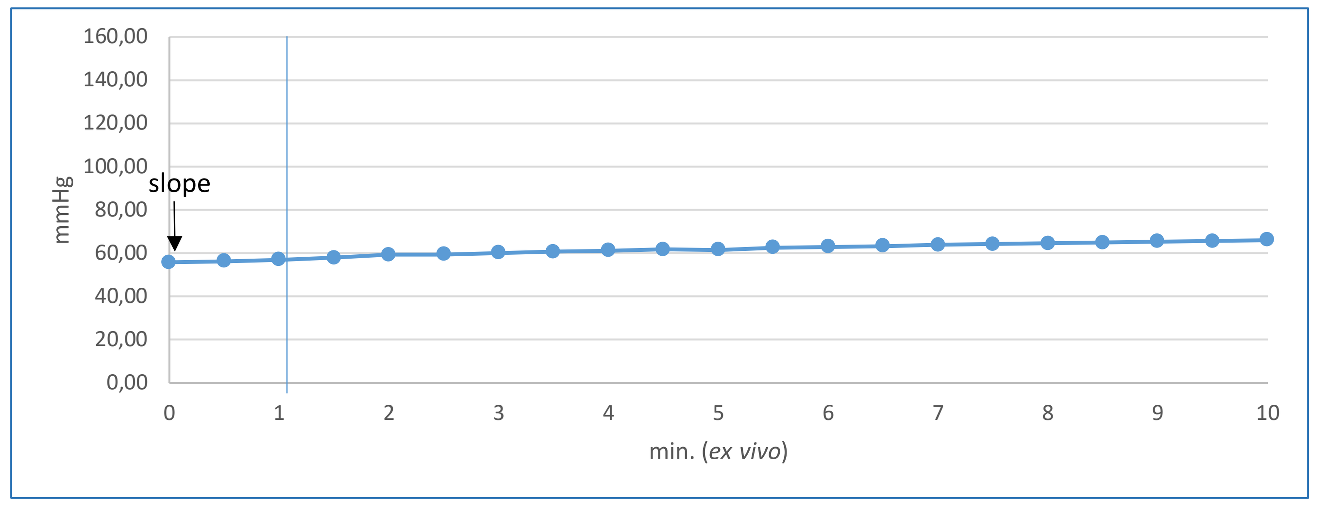

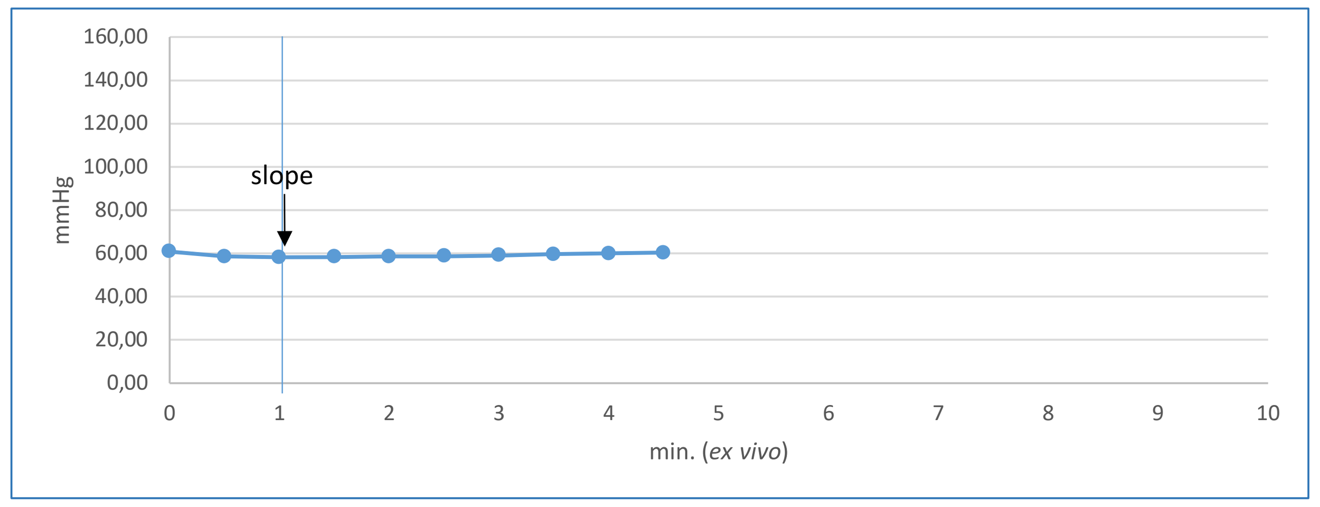

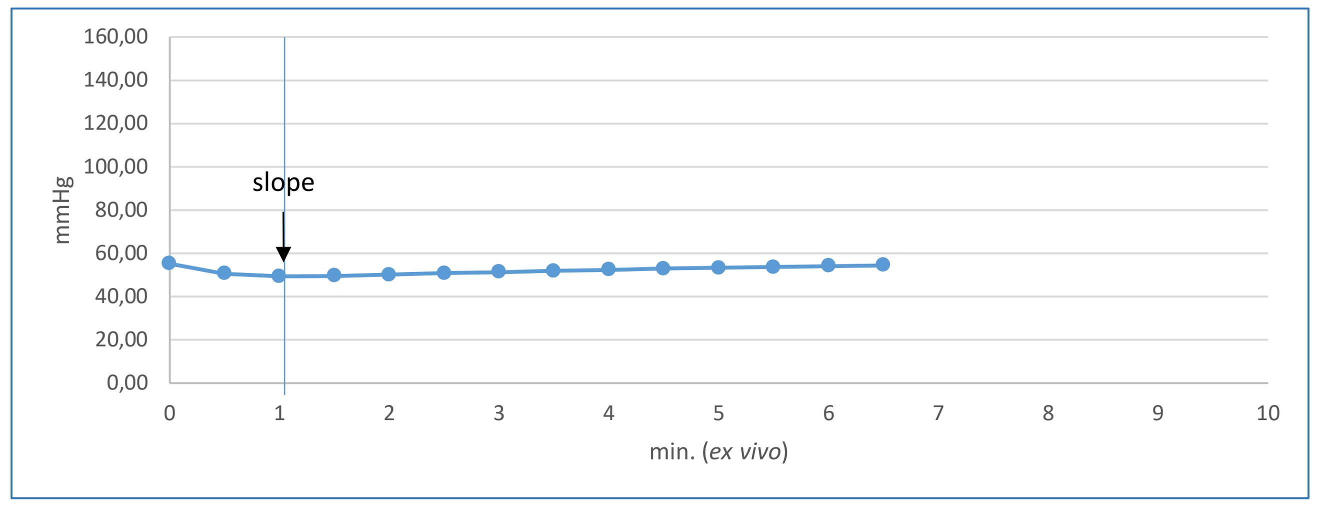

Supplemental table 3: Raw data figure 4d and 4e

H8

13 h p.i.

Presens	pO$_2$ [mmHg]	mean	n	slope	pAir =baro [hPa]	pCSF [mmHg]	temp. [°C]	pH
min								
0,0	60,96			-7,12				7,5
0,5	57,40	59,18	2	-1,96				7,5
1,0	56,42	58,26	3	0,09				7,5
1,5	56,47			0,77				7,5
2,0	56,85	57,62	5	0,97				7,5
2,5	57,33			1,10				7,5
3,0	57,88			1,14				7,5
3,5	58,46			1,10				7,5
4,0	59,00	57,86	9	1,11				7,5
4,5	59,56			1,06				7,5
5,0	60,09			1,21	1008	6	37,8	7,5
5,5	60,69			1,03				7,5
6,0	61,21	58,64	13	1,13				7,5
6,5	61,77							7,5
7,0								
7,5								
8,0								
8,5								
9,0								
9,5								
10,0								

16 h p.i.

Presens	pO$_2$ [mmHg]	mean	n	slope	pAir =baro [hPa]	pCSF [mmHg]	temp. [°C]	pH
min								
0,0	46,03			-2,12				7,4
0,5	44,97	45,50	2	-0,15				7,4
1,0	44,90	45,30	3	0,70				7,4
1,5	45,25			1,08				7,4
2,0	45,79	45,38	5	1,35				7,4
2,5	46,46			1,43				7,4
3,0	47,17			1,47				7,4
3,5	47,91			1,45				7,4
4,0	48,63	46,34	9	1,40				7,4
4,5	49,33			1,41				7,4
5,0	50,04			1,40	1009	10	38,3	7,4
5,5	50,74							7,4
6,0								
6,5								
7,0								
7,5								
8,0								
8,5								
9,0								
9,5								
10,0								

19 h p.i.

Presens	pO$_2$ [mmHg]	mean	n	slope	pAir =baro [hPa]	pCSF [mmHg]	temp. [°C]	pH
min								
0,0	43,67			-2,63				7,4
0,5	42,36	43,01	2	0,10				7,4
1,0	42,41	42,81	3	1,32				7,4
1,5	43,07			1,61				7,4
2,0	43,87	43,08	5	1,64				7,4
2,5	44,69			1,73				7,4
3,0	45,56			1,63				7,4
3,5	46,37			1,60				7,4
4,0	47,17	44,35	9	1,48				7,4
4,5	47,91			1,45				7,4
5,0	48,64			1,33	1012	13	38,7	7,4
5,5	49,31			1,31				7,4
6,0	49,96	45,77	13	1,28				7,4
6,5	50,60			1,23				7,4
7,0	51,22			1,16				7,4
7,5	51,79							7,4
8,0								
8,5								
9,0								
9,5								
10,0								

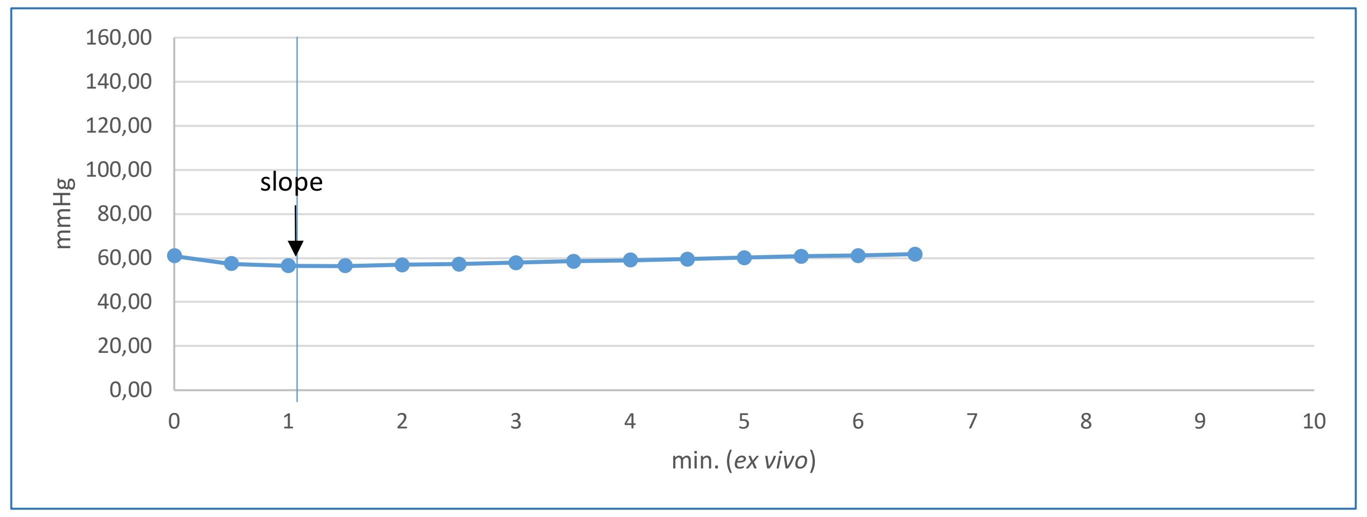

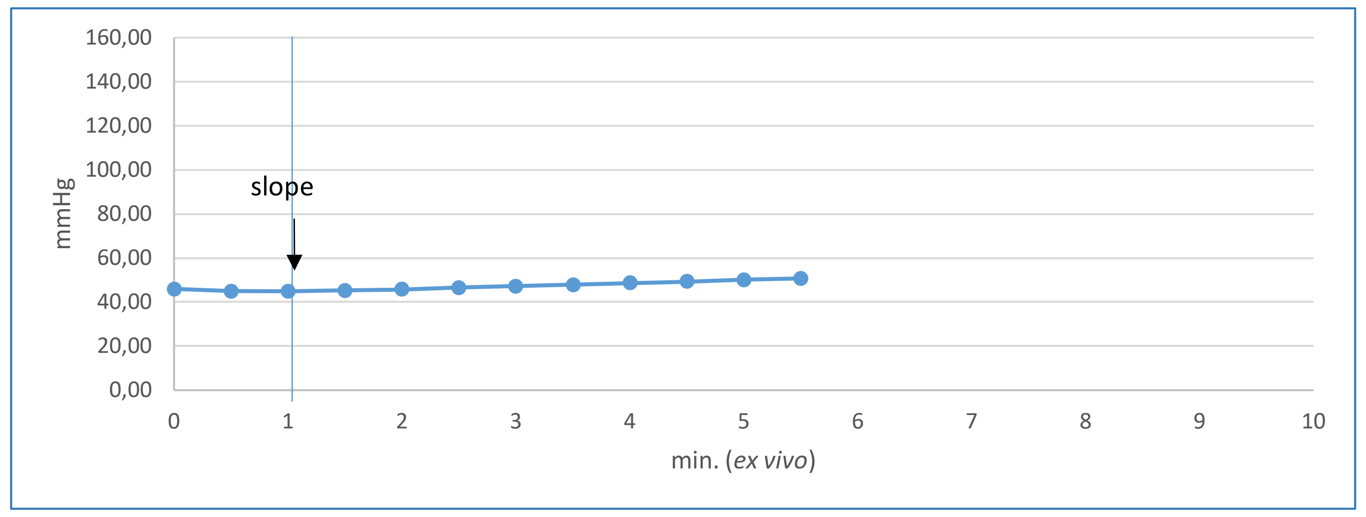

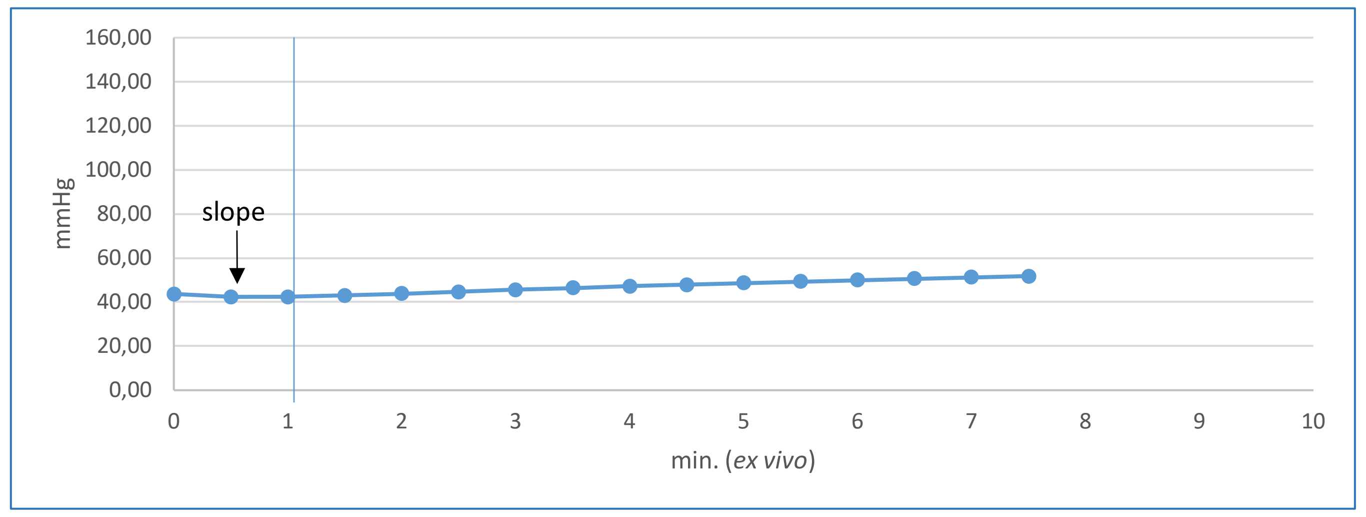

Supplemental table 3: Raw data figure 4d and 4e

H9

13 h p.i.

Presens min	pO_2 [mmHg]	mean	n	slope	pAir =baro [hPa]	pCSF [mmHg]	temp. [°C]	pH
0,0	62,07			-5,29				7,4
0,5	59,42	60,74	2	-0,67				7,4
1,0	59,08	60,19	3	0,62				7,4
1,5	59,39			1,30				7,4
2,0	60,05	60,00	5	1,00				7,4
2,5	60,55			0,75				7,4
3,0	60,92			0,52				7,4
3,5	61,18			0,27				7,4
4,0	61,32	60,44	9	0,29				7,4
4,5	61,46			0,21				7,4
5,0	61,57			0,24	1020	4	38,4	7,4
5,5	61,69			0,19				7,4
6,0	61,78	60,81	13					7,4
6,5								
7,0								
7,5								
8,0								
8,5								
9,0								
9,5								
10,0								

16 h p.i.

Presens min	pO_2 [mmHg]	mean	n	slope	pAir =baro [hPa]	pCSF [mmHg]	temp. [°C]	pH
0,0	59,74			-4,92				7,4
0,5	57,28	58,51	2	-1,88				7,4
1,0	56,34	57,79	3	-0,65				7,4
1,5	56,01			0,05				7,4
2,0	56,04	57,08	5	0,26				7,4
2,5	56,17			0,37				7,4
3,0	56,36			0,44				7,4
3,5	56,58			0,49				7,4
4,0	56,82	56,82	9	0,53				7,4
4,5	57,09			0,50				7,4
5,0	57,34			0,51	1019	7	38,8	7,4
5,5	57,59			0,58				7,4
6,0	57,88	57,02	13					7,4
6,5								
7,0								
7,5								
8,0								
8,5								
9,0								
9,5								
10,0								

19 h p.i.

Presens min	pO_2 [mmHg]	mean	n	slope	pAir =baro [hPa]	pCSF [mmHg]	temp. [°C]	pH
0,0	47,43			-3,15				7,4
0,5	45,86	46,65	2	-0,98				7,4
1,0	45,37	46,22	3	0,64				7,4
1,5	45,69			0,72				7,4
2,0	46,05	46,08	5	1,22				7,4
2,5	46,66			0,93				7,4
3,0	47,12			0,95				7,4
3,5	47,60			0,92				7,4
4,0	48,06	46,65	9	0,89				7,4
4,5	48,50			0,84				7,4
5,0	48,92			0,79	1020	9	38,0	7,4
5,5	49,32			0,72				7,4
6,0	49,68	47,40	13	0,67				7,4
6,5	50,01							7,4
7,0								
7,5								
8,0								
8,5								
9,0								
9,5								
10,0								

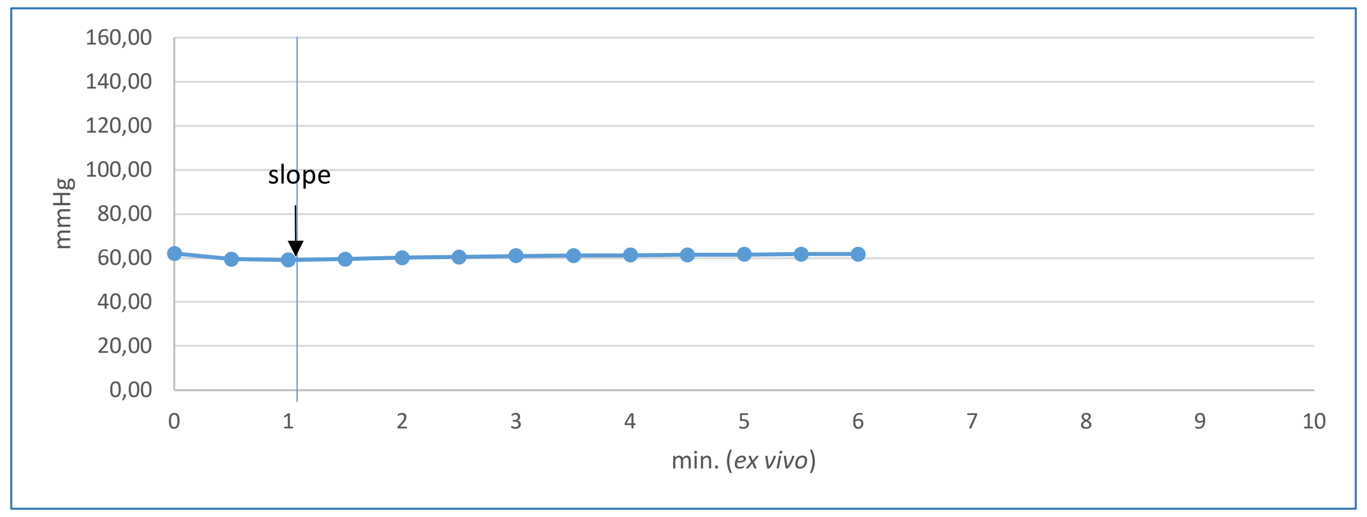

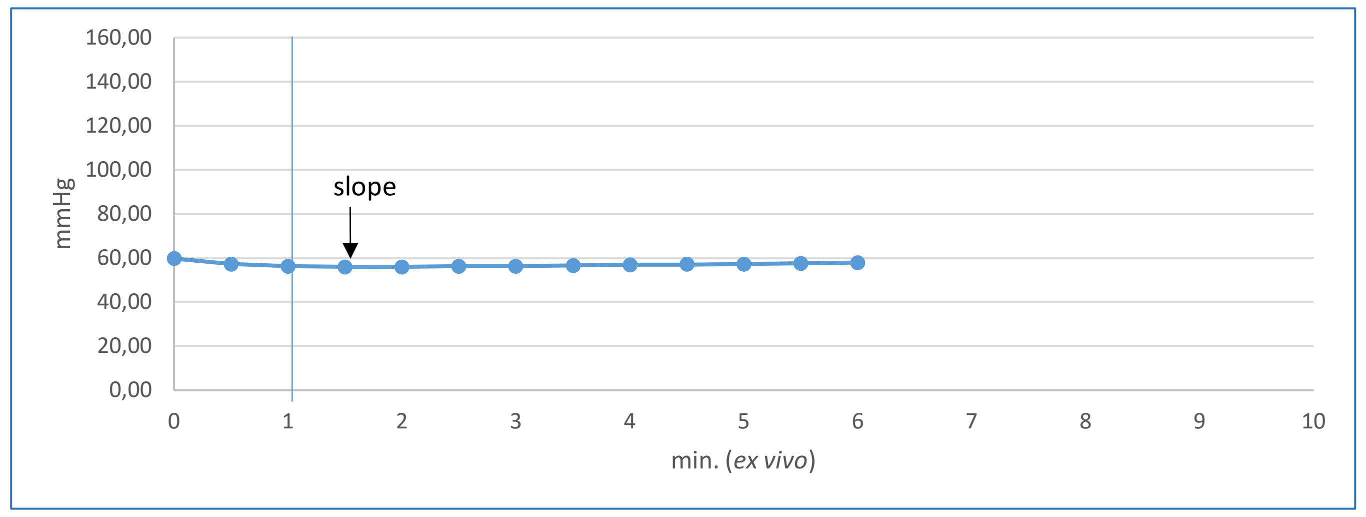

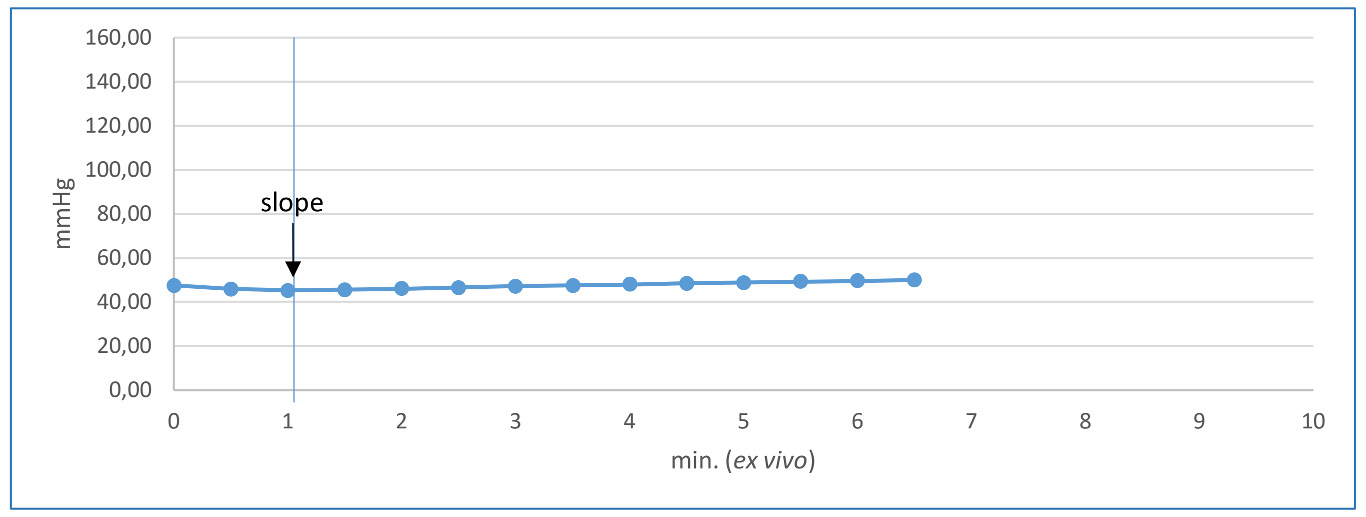

Supplemental table 3: Raw data figure 4d and 4e

H10

13 h p.i.

Presens min	pO_2 [mmHg]	mean	n	slope	pAir =baro [hPa]	pCSF [mmHg]	temp. [°C]	pH
0,0	68,66			-1,99				7,5
0,5	67,66	68,16	2	-0,01				7,5
1,0	67,65	67,99	3	0,77				7,5
1,5	68,04			0,99				7,5
2,0	68,54	68,11	5	0,95				7,5
2,5	69,01			0,94				7,5
3,0	69,48							7,5
3,5								
4,0								
4,5								
5,0					1004	5	38,2	
5,5								
6,0								
6,5								
7,0								
7,5								
8,0								
8,5								
9,0								
9,5								
10,0								

16 h p.i.

Presens min	pO_2 [mmHg]	mean	n	slope	pAir =baro [hPa]	pCSF [mmHg]	temp. [°C]	pH
0,0	58,30			-0,39				7,5
0,5	58,11	58,21	2	2,21				7,5
1,0	59,21	58,54	3	2,45				7,5
1,5	60,44							7,5
2,0								
2,5								
3,0								
3,5								
4,0								
4,5								
5,0					1001	9	38,6	
5,5								
6,0								
6,5								
7,0								
7,5								
8,0								
8,5								
9,0								
9,5								
10,0								

19 h p.i.

Presens min	pO_2 [mmHg]	mean	n	slope	pAir =baro [hPa]	pCSF [mmHg]	temp. [°C]	pH
0,0	51,19			1,72				7,5
0,5	52,05	51,62	2	2,94				7,5
1,0	53,51	52,25	3	0,59				7,5
1,5	53,81			1,03				7,5
2,0	54,33	52,98	5	1,79				7,5
2,5	55,22			1,51				7,5
3,0	55,98			1,34				7,5
3,5	56,65			1,14				7,5
4,0	57,22							7,5
4,5								
5,0					998	10	38,4	
5,5								
6,0								
6,5								
7,0								
7,5								
8,0								
8,5								
9,0								
9,5								
10,0								

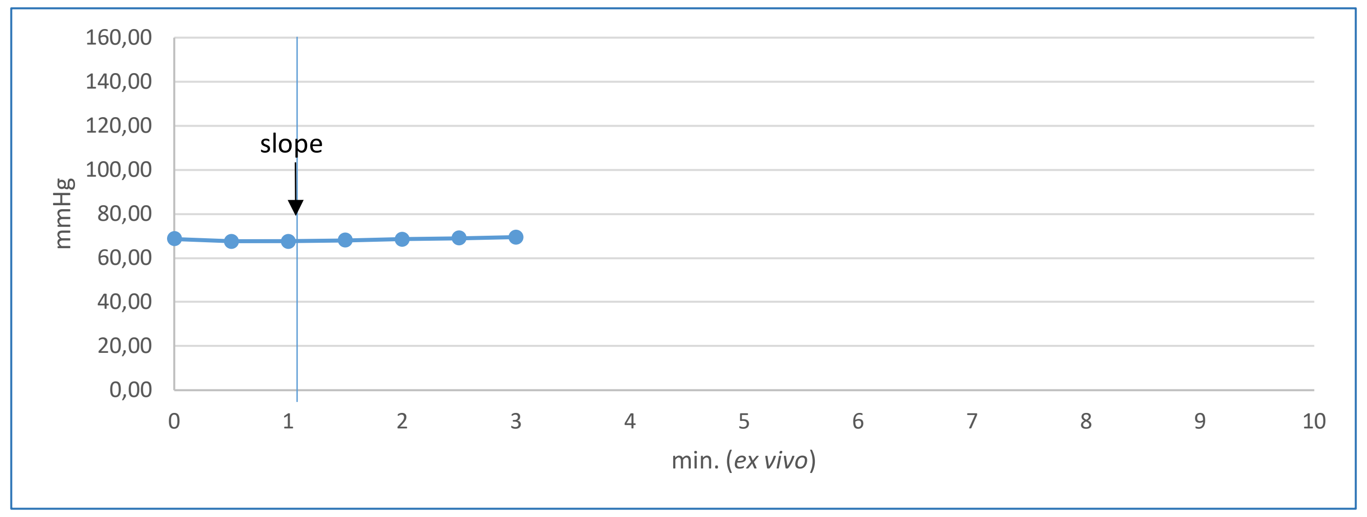

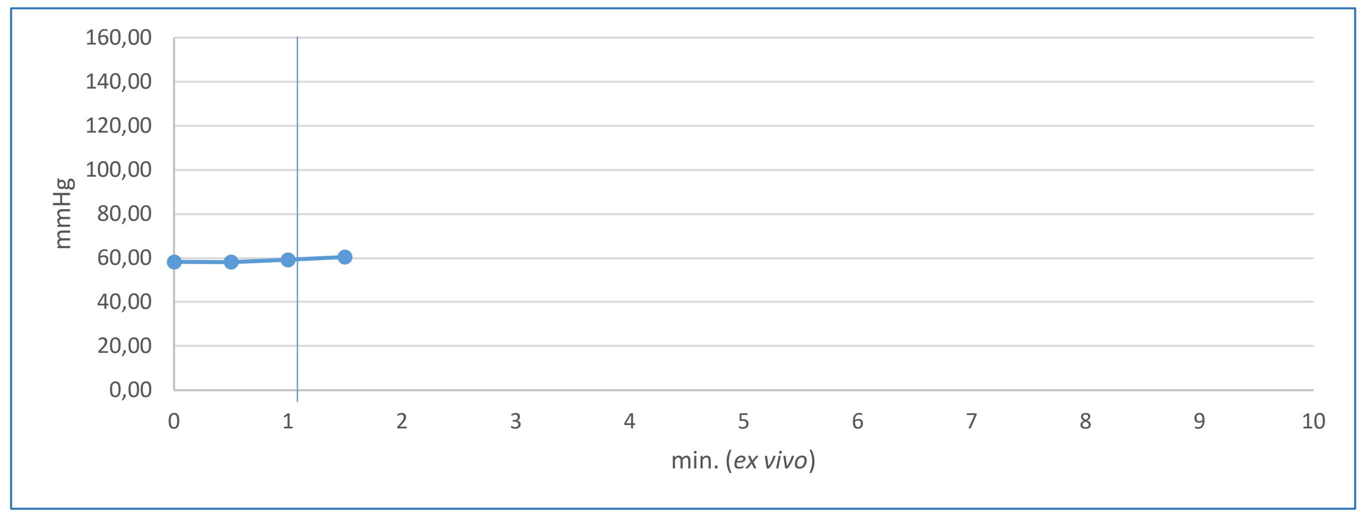

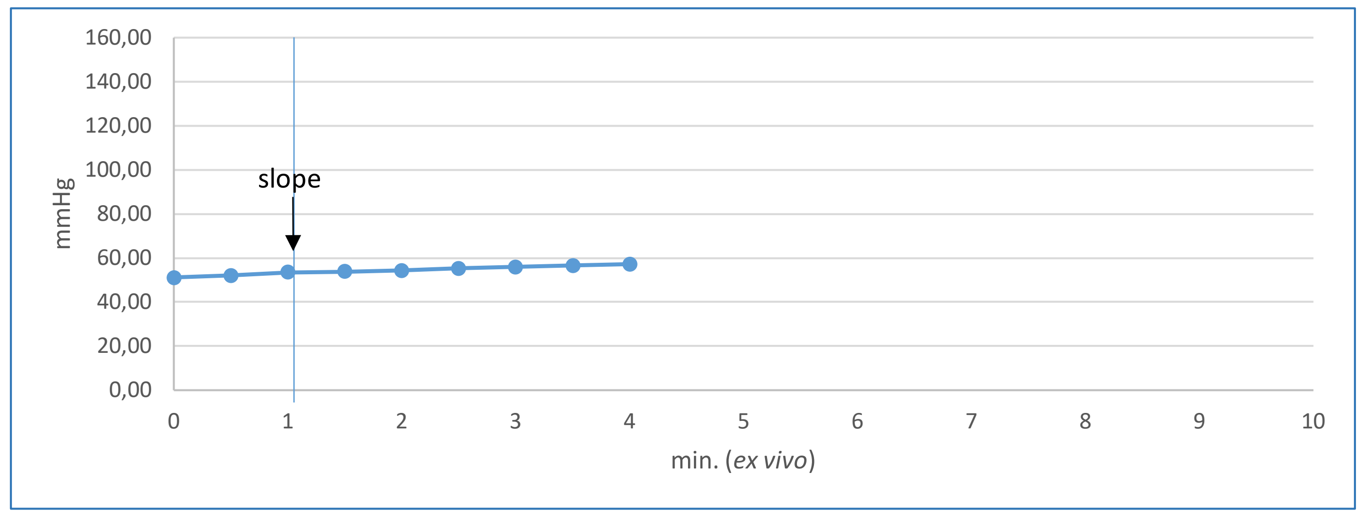

Supplemental table 3: Raw data figure 4d and 4e

H11

13 h p.i.

Presens	pO_2 [mmHg]	mean	n	slope	pAir =baro [hPa]	pCSF [mmHg]	temp. [°C]	pH
min								
0,0	63,52			-0,57				7,5
0,5	63,23	63,37	2	-0,43				7,5
1,0	63,01	63,25	3	0,27				7,5
1,5	63,15			0,33				7,5
2,0	63,31	63,24	5	0,34				7,5
2,5	63,48			0,31				7,5
3,0	63,64			0,30				7,5
3,5	63,79			0,37				7,5
4,0	63,97	63,46	9	0,39				7,5
4,5	64,17			0,45				7,5
5,0	64,40			0,44	997	7	38,8	7,5
5,5	64,61			0,38				7,5
6,0	64,80	63,78	13	0,42				7,5
6,5	65,01			0,49				7,5
7,0	65,26			0,54				7,5
7,5	65,53			0,46				7,5
8,0	65,76			0,52				7,5
8,5	66,02			0,47				7,5
9,0	66,25			0,50				7,5
9,5	66,50							7,5
10,0								

16 h p.i.

Presens	pO_2 [mmHg]	mean	n	slope	pAir =baro [hPa]	pCSF [mmHg]	temp. [°C]	pH
min								
0,0								
0,5								
1,0								
1,5								
2,0								
2,5								
3,0								
3,5								
4,0								
4,5								
5,0					**-- failed --**			
5,5								
6,0								
6,5								
7,0								
7,5								
8,0								
8,5								
9,0								
9,5								
10,0								

19 h p.i.

Presens	pO_2 [mmHg]	mean	n	slope	pAir =baro [hPa]	pCSF [mmHg]	temp. [°C]	pH
min								
0,0								
0,5								
1,0								
1,5								
2,0								
2,5								
3,0								
3,5								
4,0								
4,5								
5,0					**-- failed --**			
5,5								
6,0								
6,5								
7,0								
7,5								
8,0								
8,5								
9,0								
9,5								
10,0								

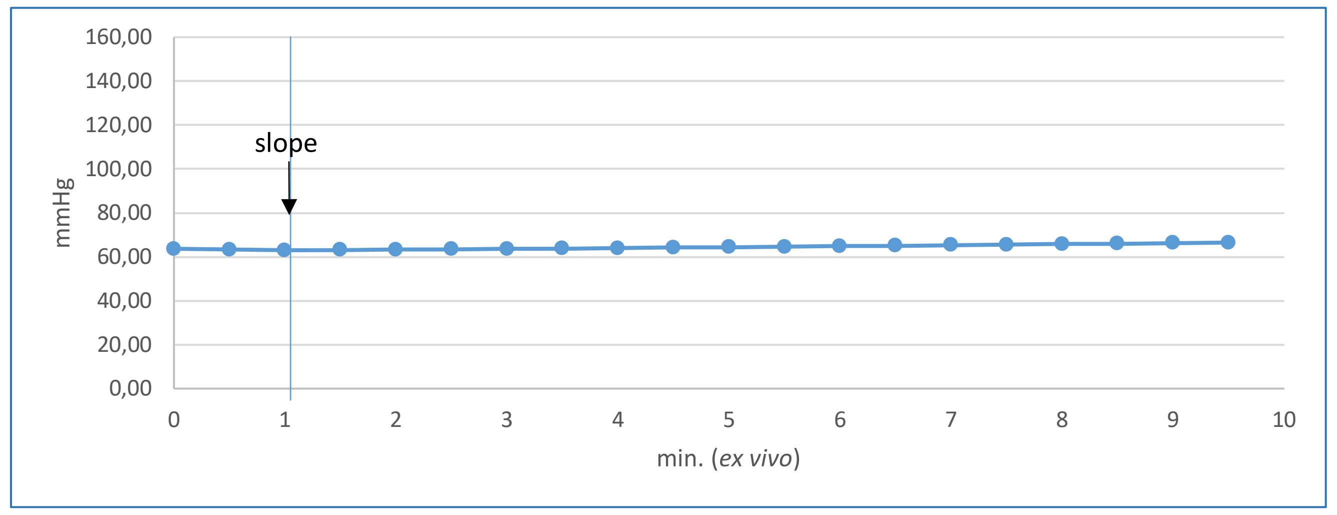

Supplemental table 3: Raw data figure 4d and 4e

H12

13 h p.i.

Presens min	pO_2 [mmHg]	mean	n	slope	pAir =baro [hPa]	pCSF [mmHg]	temp. [°C]	pH
0,0	65,56			-9,68				7,5
0,5	60,72	63,14	2	-4,63				7,5
1,0	58,41	61,56	3	-2,39				7,5
1,5	57,21			-1,28				7,5
2,0	56,57	59,69	5	-0,54				7,5
2,5	56,30			-0,18				7,5
3,0	56,21			0,05				7,5
3,5	56,24			0,19				7,5
4,0	56,33	58,17	9	0,25				7,5
4,5	56,46			0,37				7,5
5,0	56,64			0,48	992	7	38,4	7,5
5,5	56,88			0,49				7,5
6,0	57,12	57,74	13	0,61				7,5
6,5	57,43			0,61				7,5
7,0	57,73			0,68				7,5
7,5	58,07							7,5
8,0								
8,5								
9,0								
9,5								
10,0								

16 h p.i.

Presens min	pO_2 [mmHg]	mean	n	slope	pAir =baro [hPa]	pCSF [mmHg]	temp. [°C]	pH
0,0	45,41			-3,57				7,5
0,5	43,63	44,52	2	-1,12				7,5
1,0	43,07	44,03	3	0,09				7,5
1,5	43,11			0,87				7,5
2,0	43,55	43,75	5	1,21				7,5
2,5	44,15			1,37				7,5
3,0	44,84			1,46				7,5
3,5	45,57			1,40				7,5
4,0	46,27	44,40	9	1,31				7,5
4,5	46,92			1,26				7,5
5,0	47,55			1,17	992	13	39,1	7,5
5,5	48,14			1,15				7,5
6,0	48,71	45,45	13	1,04				7,5
6,5	49,23			1,03				7,5
7,0	49,75			1,06				7,5
7,5	50,28							7,5
8,0								
8,5								
9,0								
9,5								
10,0								

19 h p.i.

Presens min	pO_2 [mmHg]	mean	n	slope	pAir =baro [hPa]	pCSF [mmHg]	temp. [°C]	pH
0,0	46,50			-5,09				7,5
0,5	43,96	45,23	2	-1,98				7,5
1,0	42,97	44,47	3	-0,33				7,5
1,5	42,80			0,69				7,5
2,0	43,14	43,87	5	1,19				7,5
2,5	43,74			1,31				7,5
3,0	44,40							7,5
3,5								
4,0								
4,5								
5,0					992	11	38,7	
5,5								
6,0								
6,5								
7,0								
7,5								
8,0								
8,5								
9,0								
9,5								
10,0								

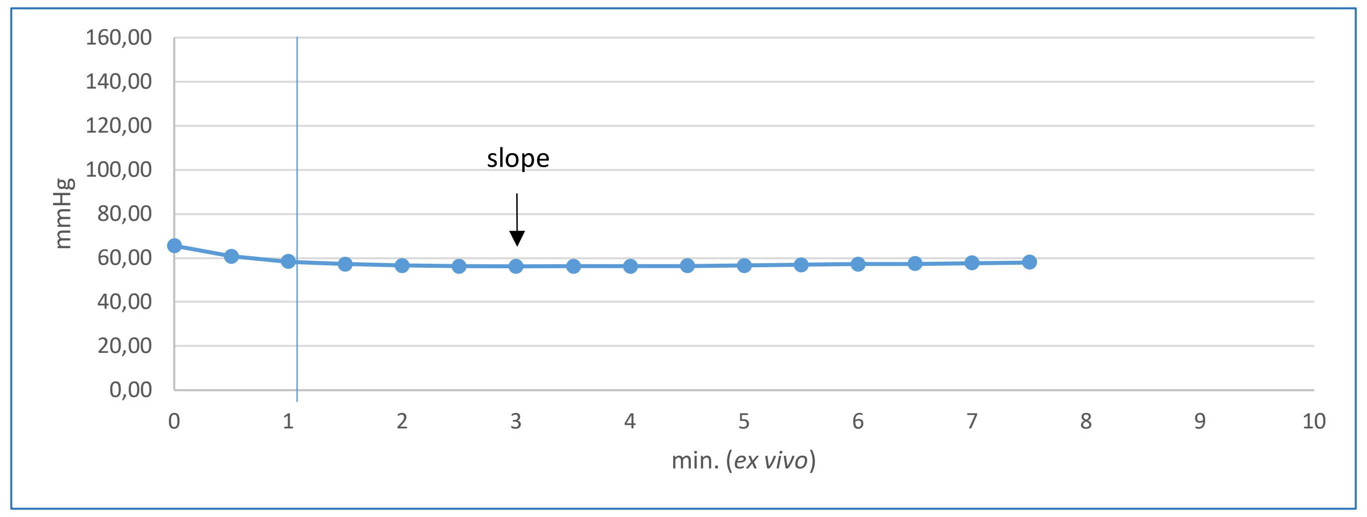

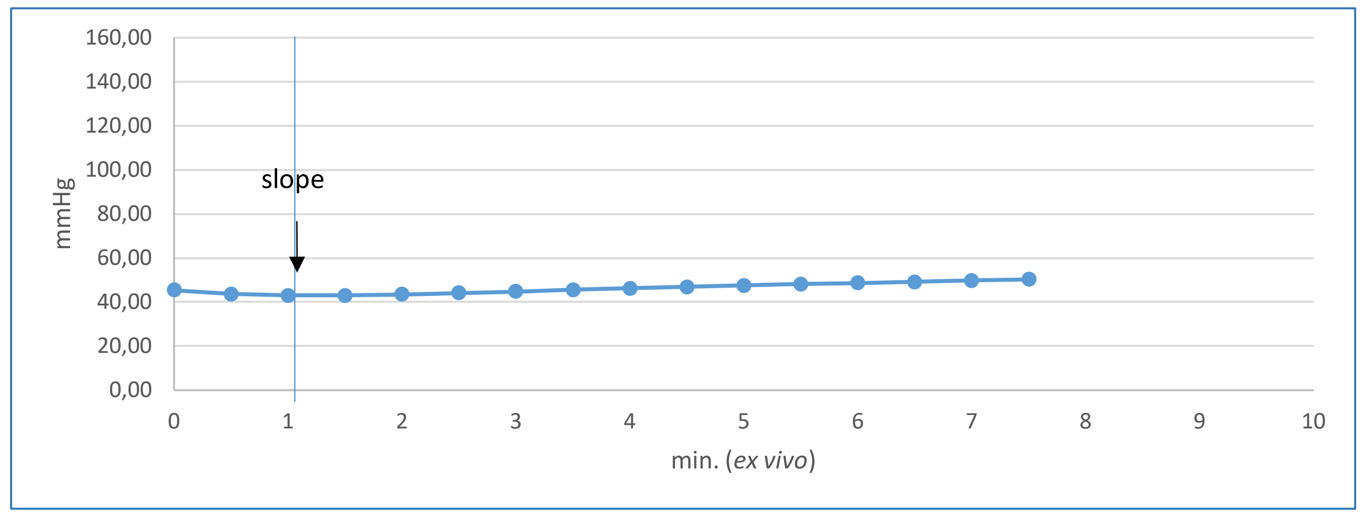

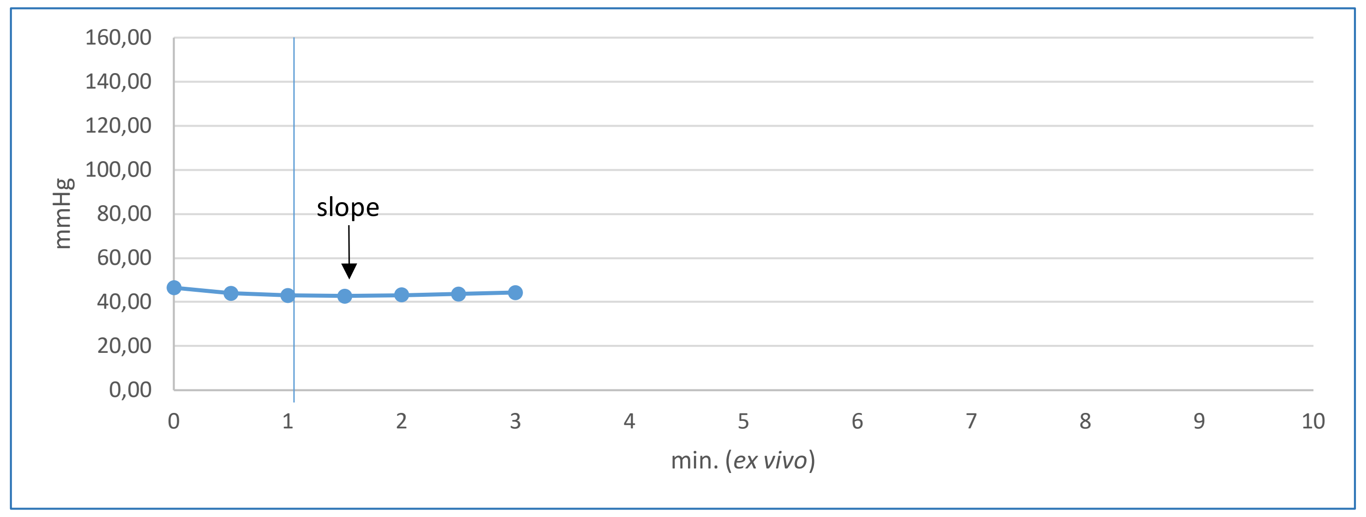

Supplement: Supplementary file 5 — Additional file 5. Table S3. Raw data Fig. 4d, e. [file 12868_2021_648_MOESM5_ESM.pdf]
