# Supplementary material for: In vivo oxygen measurement in cerebrospinal fluid of pigs to determine physiologic and pathophysiologic oxygen values during CNS infections
Source: BMC Neurosci. 2021 Jun 28;22:45. doi: 10.1186/s12868-021-00648-x (PMC8240281; doi:10.1186/s12868-021-00648-x)
Supplement: Supplementary file 6 — Additional file 6. Table S4. Analysis of various measurement cut-off's from all data in Fig 4d. [file 12868_2021_648_MOESM6_ESM.pdf]

Supplemental table 4: Analysis of various measurement cut-off's from all data in figure 4d

13 h p.i.	**1 min**	0 min	Ø 0,5 min	Ø 1 min	Ø 2 min	Ø 4 min	Ø 6 min	slope
H1	75,96	74,16	74,57	75,03	75,75	77,00	78,10	81,21
H4	51,56	49,70	50,23	50,67	51,25	52,55	53,82	51,56
H5								
H10	67,65	68,66	68,16	67,99	68,11	68,43	68,43	67,65
H11	63,01	63,52	63,37	63,25	63,24	63,46	63,78	63,01
H12	58,41	65,56	63,14	61,56	59,69	58,17	57,74	56,21
n	5	5	5	5	5	5	5	5
min	51,56	49,70	50,23	50,67	51,25	52,55	53,82	51,56
max	75,96	74,16	74,57	75,03	75,75	77,00	78,10	81,21
range	24,39	24,46	24,34	24,36	24,50	24,45	24,28	29,65
Ø	63,32	64,32	63,89	63,70	63,61	63,92	64,37	63,93
SD	9,23	9,10	8,94	8,96	9,16	9,41	9,49	11,47

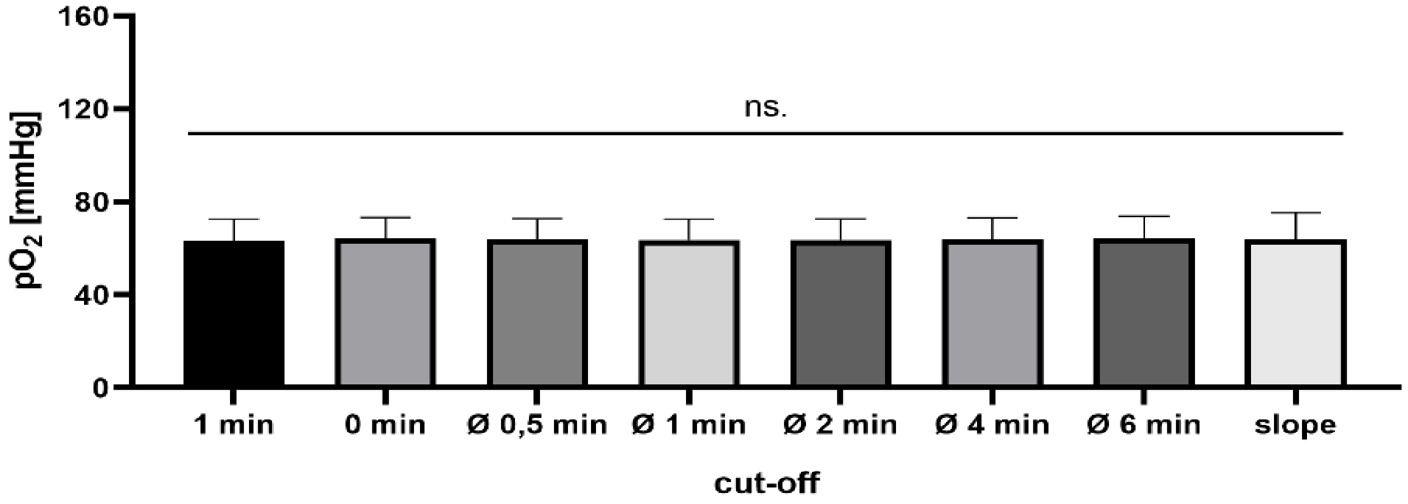

statistic: one-way ANOVA; ns. = not significant

Supplemental table 4: Analysis of various measurement cut-off's from all data in figure 4d

13 h p.i.								
	**1 min**	0 min	Ø 0,5 Min	Ø 1 Min	Ø 2 Min	Ø 4 Min	Ø 6 Min	slope
H6	50,03	52,65	51,66	51,11	50,72	51,03	51,62	49,99
H8	56,42	60,96	59,18	58,26	57,62	57,86	58,64	56,42
H9	59,08	62,07	60,74	60,19	60,00	60,44	60,81	59,08
n	3	3	3	3	3	3	3	3
min	50,03	52,65	51,66	51,11	50,72	51,03	51,62	49,99
max	59,08	62,07	60,74	60,19	60,00	60,44	60,81	59,08
range	9,05	9,42	9,09	9,08	9,28	9,42	9,19	9,09
Ø	55,18	58,56	57,19	56,52	56,11	56,44	57,02	55,16
SD	4,65	5,15	4,86	4,78	4,82	4,87	4,80	4,67

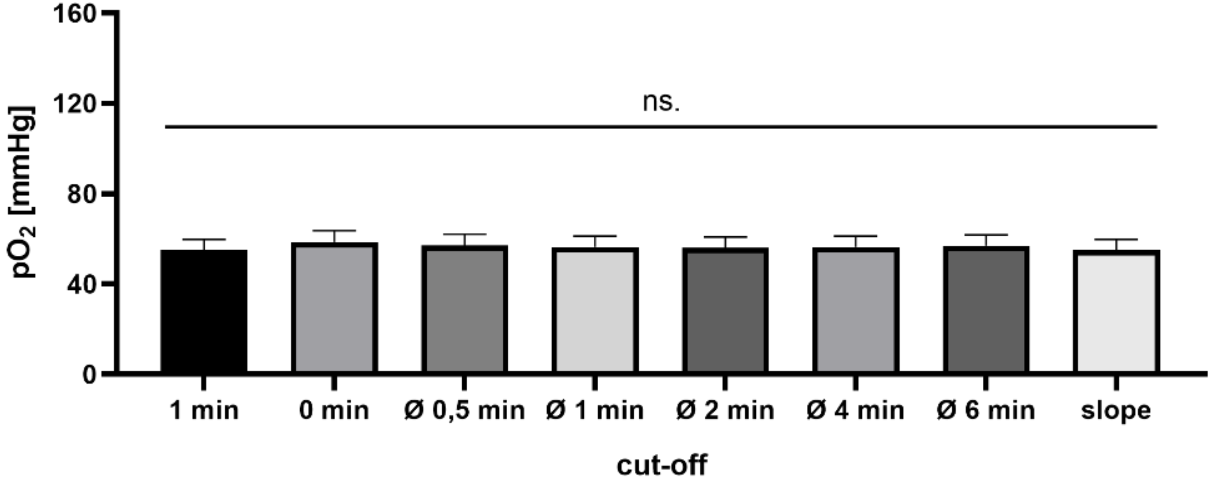

statistic: one-way ANOVA; ns. = not significant

Supplemental table 4: Analysis of various measurement cut-off's from all data in figure 4d

13 h p.i.	**1 min**	0 min	Ø 0,5 Min	Ø 1 Min	Ø 2 Min	Ø 4 Min	Ø 6 Min	slope
H2								
H3								
H7	56,94	55,76	55,96	56,28	57,18	58,57	59,67	55,76
n	1	1	1	1	1	1	1	1
min	56,94	55,76	55,96	56,28	57,18	58,57	59,67	55,76
max	56,94	55,76	55,96	56,28	57,18	58,57	59,67	55,76
range	0,00	0,00	0,00	0,00	0,00	0,00	0,00	0,00
Ø	56,94	55,76	55,96	56,28	57,18	58,57	59,67	55,76
SD	#DIV/0!	#DIV/0!	#DIV/0!	#DIV/0!	#DIV/0!	#DIV/0!	#DIV/0!	#DIV/0!

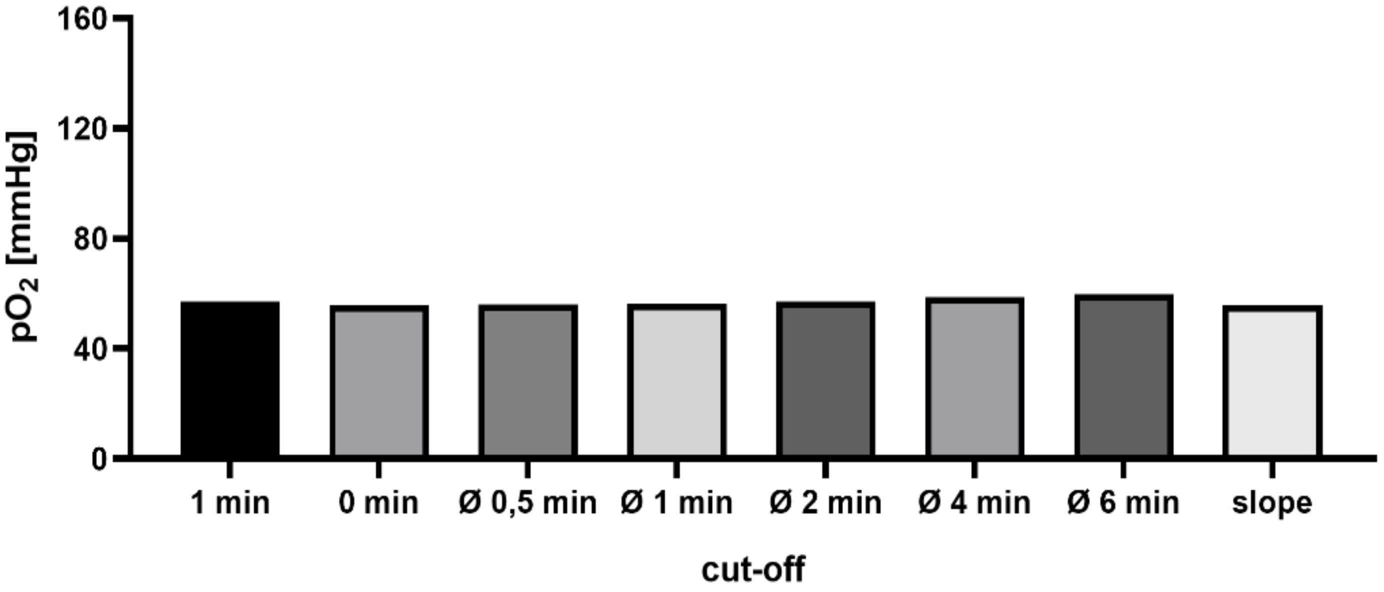

Supplemental table 4: Analysis of various measurement cut-off's from all data in figure 4d

16 h p.i.								
	**1 min**	0 min	Ø 0,5 min	Ø 1 min	Ø 2 min	Ø 4 min	Ø 6 min	slope
H1	56,55	55,65	55,89	56,11	56,58	57,46	58,29	57,50
H4	64,94	71,63	69,36	67,89	66,26	64,87	64,29	63,19
H5	54,20	53,72	53,73	53,89	54,55	56,36	57,92	53,72
H10	59,21	58,30	58,21	58,54				
H11								
H12	43,07	45,41	44,52	44,03	43,75	44,40	45,45	43,07
n	5	5	5	5	4	4	4	4
min	43,07	45,41	44,52	44,03	43,75	44,40	45,45	43,07
max	64,94	71,63	69,36	67,89	66,26	64,87	64,29	63,19
range	21,88	26,22	24,84	23,86	22,51	20,47	18,84	20,13
Ø	55,59	56,94	56,34	56,09	55,28	55,77	56,49	54,37
SD	8,07	9,52	8,94	8,59	9,23	8,47	7,91	8,48

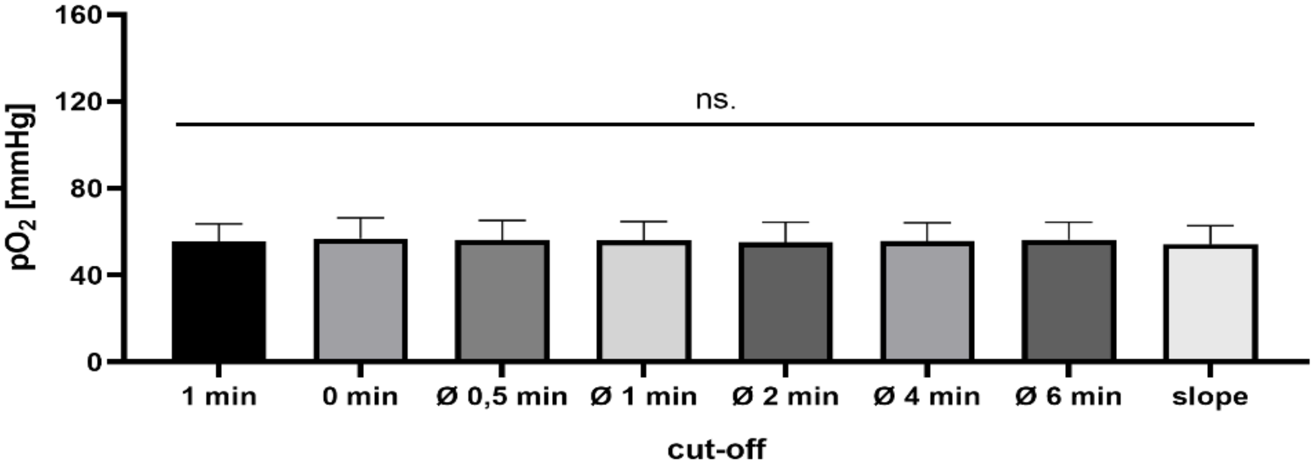

statistic: one-way ANOVA; ns. = not significant

Supplemental table 4: Analysis of various measurement cut-off's from all data in figure 4d

16 h p.i.								
	**1 min**	0 min	Ø 0,5 Min	Ø 1 Min	Ø 2 Min	Ø 4 Min	Ø 6 Min	slope
H6	70,95	72,59	71,96	71,62	71,34	71,37		70,92
H8	44,90	46,03	45,50	45,30	45,38	46,34	47,27	44,90
H9	56,34	59,74	58,51	57,79	57,08	56,82	57,02	56,01
n	3	3	3	3	3	3	2	3
min	44,90	46,03	45,50	45,30	45,38	46,34	47,27	44,90
max	70,95	72,59	71,96	71,62	71,34	71,37	57,02	70,92
range	26,06	26,56	26,46	26,32	25,96	25,02	9,75	26,02
Ø	57,40	59,45	58,65	58,24	57,94	58,18	52,14	57,28
SD	13,06	13,28	13,23	13,17	13,00	12,57	6,89	13,06

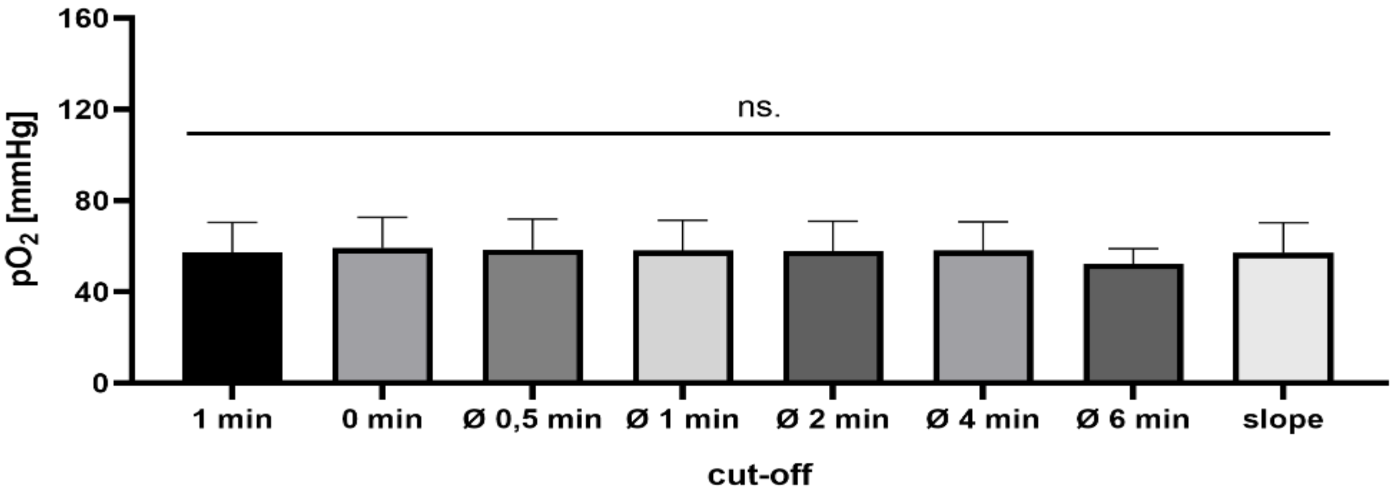

statistic: one-way ANOVA; ns. = not significant

Supplemental table 4: Analysis of various measurement cut-off's from all data in figure 4d

16 h p.i.	**1 min**	0 min	Ø 0,5 Min	Ø 1 Min	Ø 2 Min	Ø 4 Min	Ø 6 Min	slope
H2	71,16	69,31	69,58	70,11	64,84	62,01	61,36	56,85
H3	39,69	35,79	36,74	37,73	39,47	42,05	43,73	47,32
H7	58,21	60,82	59,68	59,19	58,88	59,07		58,21
n	3	3	3	3	3	3	2	3
min	39,69	35,79	36,74	37,73	39,47	42,05	43,73	47,32
max	71,16	69,31	69,58	70,11	64,84	62,01	61,36	58,21
range	31,47	33,52	32,83	32,38	25,37	19,96	17,63	10,89
Ø	56,35	55,30	55,33	55,67	54,40	54,38	52,54	54,13
SD	15,82	17,43	16,84	16,47	13,27	10,78	12,47	5,93

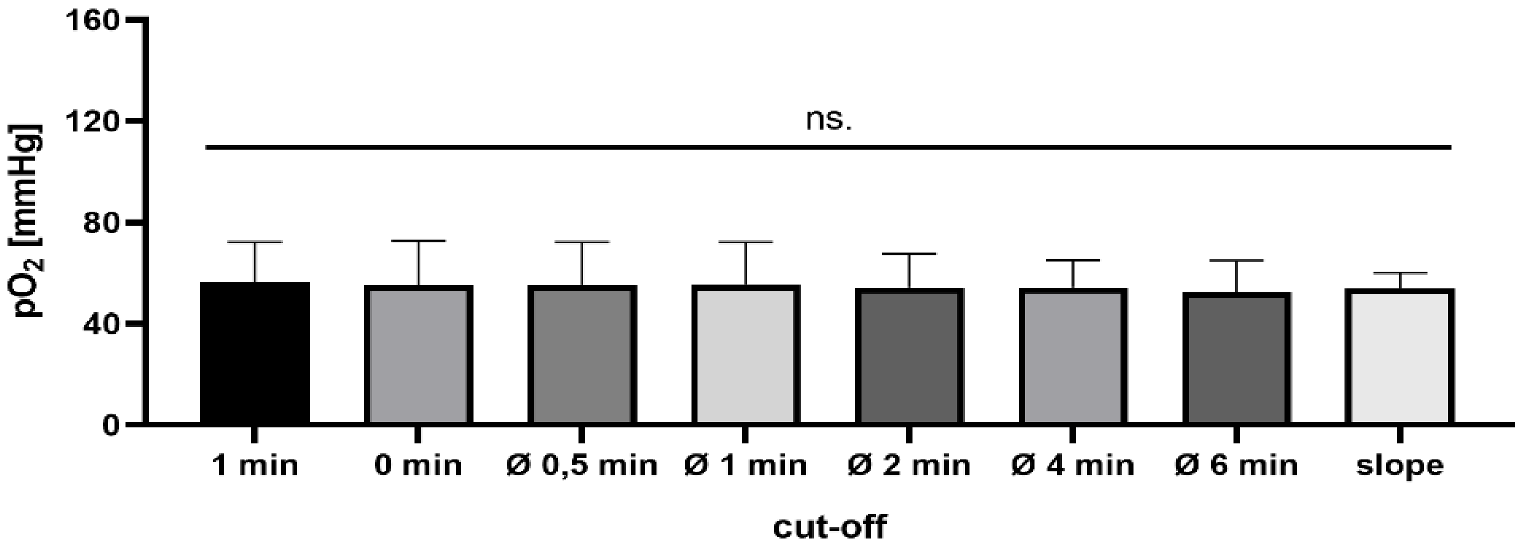

statistic: one-way ANOVA; ns. = not significant

Supplemental table 4: Analysis of various measurement cut-off's from all data in figure 4d

19 h p.i.								
	**1 min**	0 min	Ø 0,5 min	Ø 1 min	Ø 2 min	Ø 4 min	Ø 6 min	slope
H1	54,52	53,26	53,50	53,84	54,56	55,85	56,65	57,75
H4	58,13	61,86	60,71	59,85	58,73	57,73	57,42	56,43
H5	60,62	61,02	60,87	60,79	60,99	61,72	62,31	62,10
H10	53,51	51,19	51,62	52,25	52,98	54,44	54,44	53,51
H11								
H12	42,97	46,50	45,23	44,47	43,87			42,80
n	5	5	5	5	5	4	4	5
min	42,97	46,50	45,23	44,47	43,87	54,44	54,44	42,80
max	60,62	61,86	60,87	60,79	60,99	61,72	62,31	62,10
range	17,65	15,36	15,64	16,31	17,12	7,28	7,87	19,30
Ø	53,95	54,77	54,39	54,24	54,23	57,43	57,70	54,52
SD	6,77	6,57	6,60	6,59	6,61	3,16	3,32	7,24

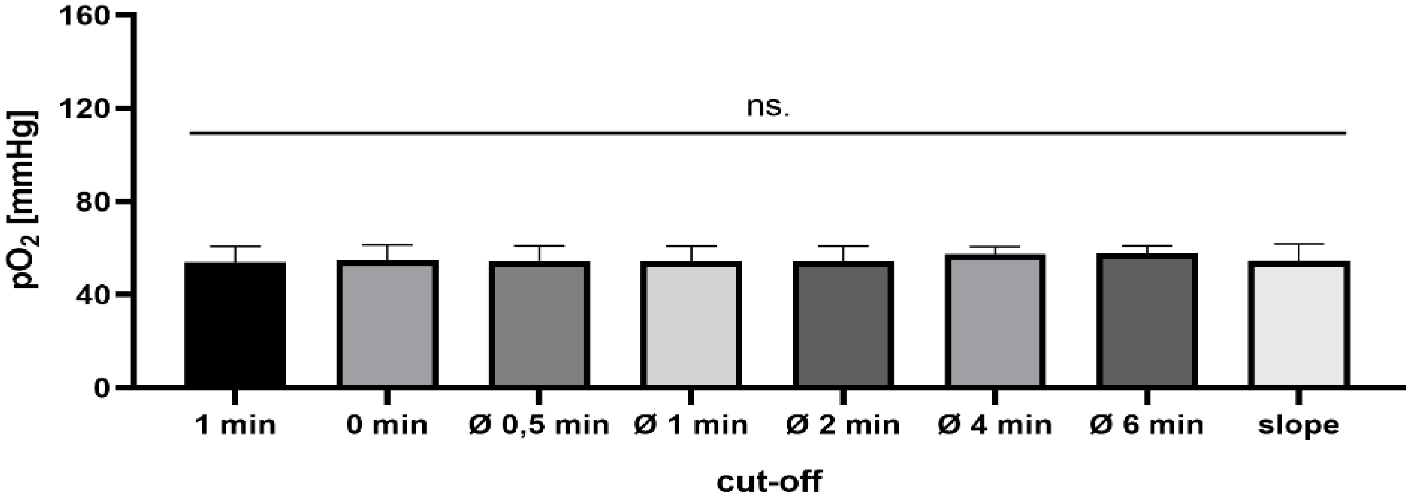

statistic: one-way ANOVA; ns. = not significant

Supplemental table 4: Analysis of various measurement cut-off's from all data in figure 4d

19 h p.i.	**1 min**	0 min	Ø 0,5 Min	Ø 1 Min	Ø 2 Min	Ø 4 Min	Ø 6 Min	slope
H6	53,00	57,31	55,83	54,89	53,92	53,55	53,84	52,46
H8	42,41	43,67	43,01	42,81	43,08	44,35	45,77	42,36
H9	45,37	47,43	46,65	46,22	46,08	46,65	47,40	45,37
n	3	3	3	3	3	3	3	3
min	42,41	43,67	43,01	42,81	43,08	44,35	45,77	42,36
max	53,00	57,31	55,83	54,89	53,92	53,55	53,84	52,46
range	10,60	13,63	12,81	12,07	10,84	9,20	8,07	10,10
Ø	46,93	49,47	48,50	47,97	47,69	48,19	49,01	46,73
SD	5,47	7,04	6,60	6,22	5,60	4,79	4,27	5,19

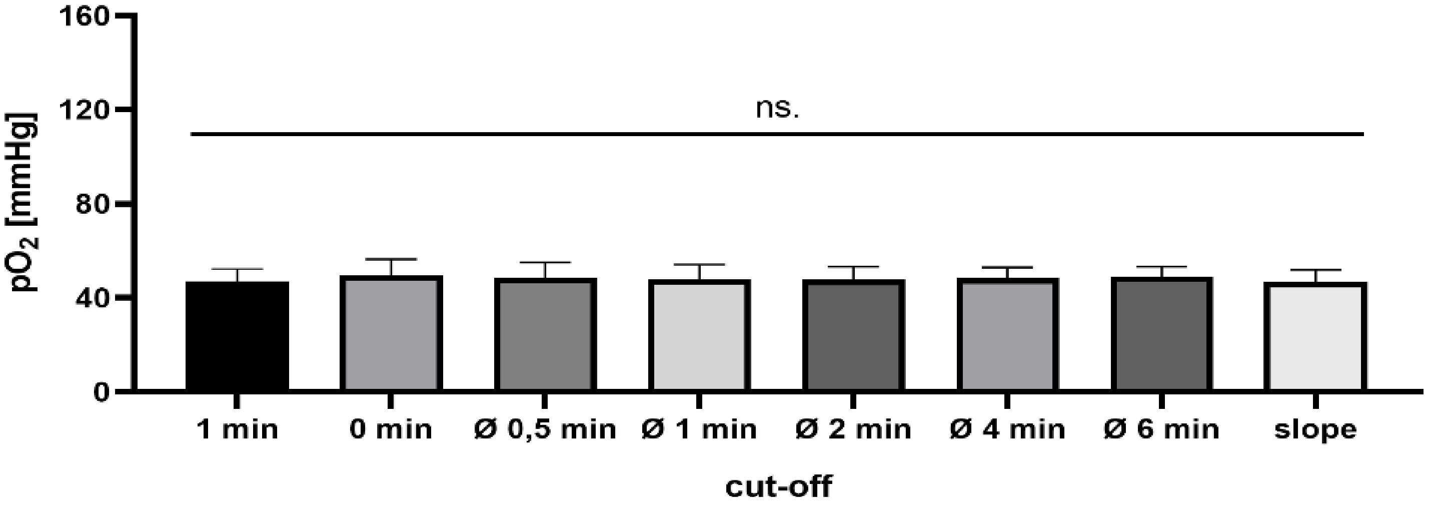

statistic: one-way ANOVA; ns. = not significant

Supplemental table 4: Analysis of various measurement cut-off's from all data in figure 4d

19 h p.i.	**1 min**	0 min	Ø 0,5 Min	Ø 1 Min	Ø 2 Min	Ø 4 Min	Ø 6 Min	slope
H2	55,33	53,14	53,44	54,07	55,42	56,99	57,86	58,56
H3	48,40	57,31	52,08	51,86	49,51	47,17		
H7	49,40	55,21	52,95	51,77	50,98	51,28	51,96	49,40
n	3	3	3	3	3	3	2	2
min	48,40	53,14	52,08	51,77	49,51	47,17	51,96	49,40
max	55,33	57,31	53,44	54,07	55,42	56,99	57,86	58,56
range	6,93	4,17	1,36	2,30	5,90	9,82	5,90	9,16
Ø	51,04	55,22	52,83	52,57	51,97	51,81	54,91	53,98
SD	3,75	2,09	0,69	1,30	3,07	4,93	4,17	6,48

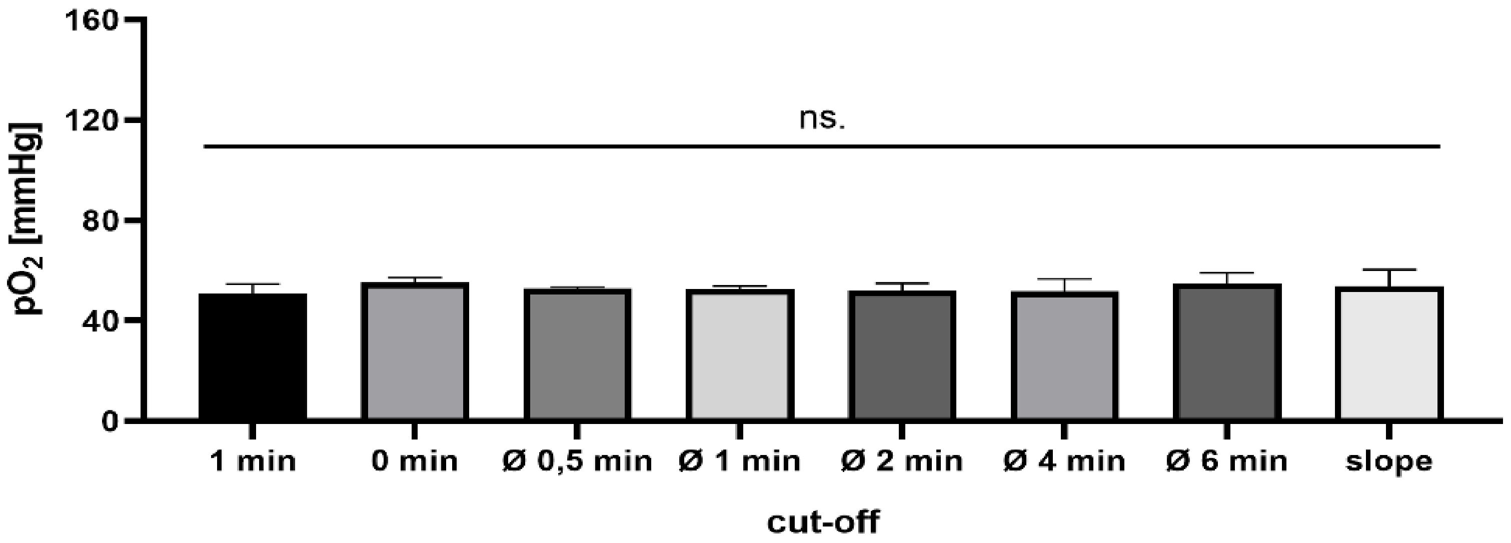

statistic: one-way ANOVA; ns. = not significant
