# Supplementary material for: In vivo oxygen measurement in cerebrospinal fluid of pigs to determine physiologic and pathophysiologic oxygen values during CNS infections
Source: BMC Neurosci. 2021 Jun 28;22:45. doi: 10.1186/s12868-021-00648-x (PMC8240281; doi:10.1186/s12868-021-00648-x)
Supplement: Supplementary file 7 — Additional file 7. Table S5. Individual pO2 values in CSF and blood and mean values per group. [file 12868_2021_648_MOESM7_ESM.pdf]

Supplemental table 5: Individual pO_2 values in CSF and blood and mean values per group

animal	infection	13 hours p.i.		16 hours p.i.		19 hours p.i.	
		pO_2 [mmHg]		pO_2 [mmHg]		pO_2 [mmHg]	
		CSF	blood	CSF	blood	CSF	blood
**H1**	uninfected (healthy)	81,21	94,50	60,73	104,20	58,62	92,60
**H4**		55,19	89,10	62,84	103,80	56,45	94,60
**H5**		60,22	91,90	56,04	96,80	61,60	102,10
**H10**		67,66	84,20	57,81	103,20	52,88	105,90
**H11**		63,02	92,00	-	-	-	-
**H12**		56,21	89,70	43,03	98,30	42,79	98,60
**Ø**		**63,92**	**90,23**	**56,09**	**101,26**	**54,47**	**98,76**
**H2**	infected (+*S. suis* in CSF)	-	102,10	60,89	98,60	60,09	102,50
**H3**		-	95,00	48,05	98,80	44,32	100,00
**H7**		58,61	103,60	58,51	90,10	50,09	92,20
**Ø**		**58,61**	**100,23**	**55,82**	**95,83**	**51,50**	**98,23**
**H6**	infected (-*S. suis* in CSF)	50,26	105,00	70,93	93,80	52,46	94,00
**H8**		56,42	104,30	44,90	98,50	42,51	98,00
**H9**		59,08	94,90	56,02	103,00	47,12	103,00
**Ø**		**55,26**	**101,40**	**57,28**	**98,43**	**47,36**	**98,33**
