# Supplementary material for: In vivo oxygen measurement in cerebrospinal fluid of pigs to determine physiologic and pathophysiologic oxygen values during CNS infections
Source: BMC Neurosci. 2021 Jun 28;22:45. doi: 10.1186/s12868-021-00648-x (PMC8240281; doi:10.1186/s12868-021-00648-x)
Supplement: Supplementary file 8 — Additional file 8. Table S6. Reisolation of the challenge strain from piglets after intravenous challenge with S. suis serotype 2. [file 12868_2021_648_MOESM8_ESM.pdf]

Supplemental table 6: Reisolation of the challenge strain from piglets after intravenous challenge with *S. suis* serotype 2.

S. suis strain	Number of piglets positive for the isolation of the challenge strain in an inner organ[b] or in serosa or in joint fluid	Number of piglets in which the *S. suis* challenge strain[a] was isolated from								
		Tonsils	Lung [c]	Serosa [d]	Spleen	Liver	Brain, CSF [e]	Joint fluid [f]	Endocard	Blood
10	4/6	1/6	2/6	1/6	3/6	3/6	3/6	4/6	2/6	5/6
control	0/6	0/6	0/6	0/6	0/6	0/6	0/6	0/6	0/6	0/6

[a] The challenge strain was identified by PCR.

[b] Inner organ refers to lung, spleen, liver, brain, CSF or endocard but not the tonsils.

[c] One cranial lobe was investigated.

[d] Pleural, peritoneal or pericardial cavity.

[e] Cerebrospinal fluid.

[f] Punctures of both tarsal and carpal joints were investigated in each animal.
In case of lameness additional joint punctures of the respective limb were screened.
